# Investigation of the cause of reduced sugar content in *Kiyomi tangor* fruit of Ziyang xiangcheng (*Citrus junos* Sieb. ex Tanaka) rootstock

**DOI:** 10.1038/s41598-019-55957-3

**Published:** 2019-12-17

**Authors:** Tiantian Dong, Bo Xiong, Shengjia Huang, Ling Liao, Xia Qiu, Guochao Sun, Yunzhenzi He, Changwen Duan, Xiaojia Wang, Xu Zhang, Sichen Li, Jin Zhu, Zhihui Wang

**Affiliations:** 1 College of Horticulture, Sichuan Agricultural University, Chengdu, 611130 Sichuan China; 2 Institute of Pomology and Olericulture, Sichuan Agricultural University, Chengdu, 611130 Sichuan China; 3 Sichuan Horticultural Crop Extension Station, 610041 Sichuan, China

**Keywords:** Plant development, Plant physiology

## Abstract

Ziyang xiangcheng (*Citrus junos* Sieb. ex Tanaka) (Cj) rootstock is effective in *Citrus* production; however, when Cj rootstock was used, sugar content in *Kiyomi tangor* fruit was significantly lower than that in the fruit produced using *Poncirus trifoliata* (L.) Raf. rootstock (Pt). Therefore, using *K*. *tangor*, we explored the cause of this difference, determining sugar accumulation, sucrose-metabolism enzyme activities, and gene expression. Before ripening, sugar content in fruits with Cj rootstock was significantly lower than that in fruits with Pt rootstock, due to low fructose and sucrose content. Sucrose phosphate synthase (SPS) activity of Pt was higher than that of Cj in the early growth stage (at 90–210 days after anthesis), however it was opposite at 240–300 days after anthesis. Additionally, neutral invertase (NI) activity of Pt was higher than that of Cj. Gene expression in Pt was higher than that in Cj, but is was essentially the same at maturity. SPS and NI activities and *CitSPS1* expression were positively correlated with sucrose, fructose, and glucose content, but *CSCW1* expression was negatively correlated with the sugars. Overall, the weak flavour of *K*. *tangor* fruit with Cj rootstock was regulated by the sucrose metabolism-related enzymes and gene expression.

## Introduction

*Citrus* is a commercially important genus of the Rutaceae family and comprises fruit species that are cultivated globally^1,2^. *Kiyomi tangor* (*Citrus unshiu* Marcov. forma *miyagawawase* × *Citrus sinensis* Osbeck) is the first orange hybrid bred using the Satsuma mandarin (*Citrus unshiu* Marcov. forma miyagawawase) and the Trovita orange (*Citrus sinensis* Osbeck) in Japan^3^. *Kiyomi tangor* (*K*. *tangor*)fruits ripen during the off-season; it is therefore an off-season fruit in the *Citrus* market, and its prospect is very promising^4^. *Trifoliate orange* (*Poncirus trifoliata* (L.) Raf.) (Pt) is the most common rootstock used for *Citrus* production because it is resistant to cold and foot rot^5^. However, when *K*. *tangor* was grafted on Pt rootstock and planted in alkaline soil, the hindered absorption of trace mineral elements led to the element deficiency disease^6^.

*Citrus* production is affected by many environmental factors including drought, salt, alkali and extreme temperature^7^. Grafting is one of the most effective technique for enhancing citrus reproduction and thus production. Rootstock is an important part of the *Citrus* industry as it influences scion characteristics such as fruit quality, canopy size, and resistance^8,9^. Previous studies have demonstrated that *Citrus*-grafted seedlings with Pt as rootstock were prone to nutrient deficiency and growth retardation in calcareous soils^10,11^. However, Ziyang xiangcheng (*Citrus junos* Sieb. ex Tanaka) (Cj) is a local *Citrus* rootstock originating from Southwest China with excellent disease-, alkali-, drought-, and cold-resistant characteristics^10^. Additionally, Cj is more alkali-resistant than Pt. Moreover, It was not until the soil pH increased to 8.3 that the leaf etiolation appeared on *Citrus* with Cj as rootstock^12^. Therefore, in recent years, Cj has been used as a rootstock in more and more scion varieties in China due to its excellent tolerance to alkaline stress and iron deficiency^10^. However, we found that the sugar content of *K*. *tangor* fruit with Cj as rootstock was lower than that of Pt in the field experiment.

The ripening process of citrus fruits is usually accompanied by the accumulation of sugars and the degradation of organic acids^13^. Sugar, which is mainly in the form of sucrose, fructose, and glucose, plays an key role in the quality and flavour, and the differences in sugar content determine the sweetness and colour of fruit^14,15^. Sucrose is the key determinant of yield, accounting for approximately 90% of the carbohydrates in plants^16^. By changing the activity balance of sucrose phosphate synthase (SPS) and sucrose synthase (SS), sucrose metabolism enzymes regulate the decomposition and synthesis of sucrose and realise the redistribution of carbohydrates^17,18^.

Among the enzymes involved in sucrose metabolism, SS and invertase (INVs) are involved in the cleavage of sucrose, while SPS catalyses the synthesis of sucrose^19^. Sucrose phosphate synthase, which is regulated by metabolites and reversible protein phosphorylation in plant tissues and promoted by glucose-6-phosphate, acts on UDP-glucose and fructose-6-phosphate to produce sucrose-phosphate in the sucrose biosynthesis pathway^20,21^. SS is widely regarded as a key enzyme involved in sucrose metabolism, which may play an important role in carbon distribution in sugar synthesis, sucrose conversion, and adenosine preservation of the respiratory pathways^22,23^. SS participates in the process degradation of sucrose to UDP-glucose and fructose^21^. Sucrose synthase has bidirectional function, the direction of sucrose cleavage (SS-I) and the direction of sucrose synthesis (SS-II)^22^. INVs is important for carbohydrate supply to sink tissues; they play a key role in regulating, amplifying, and integrating different signals, which leads to the transportation of the end product of photosynthesis from source to sink^22,24^. INVs act on sucrose to form glucose and fructose^21,25^. There are two main groups of INVs in plants, namely acidic invertase (AI) and neutral invertase (NI)^26^, which play a role under different pH conditions^23,27^. Several studies have reported the correlation between sugar content and metabolism enzyme activity in mature fruit and that sucrose-metabolism enzymes play an important role in sugar metabolism^28,29^.

Sucrose accumulation in fruit is related to gene expression of sucrose-metabolism enzymes^30^. In recent years, sugar metabolism-related genes were cloned in *Citrus* including the sucrose synthesis directional genes (*CitSPS1*, *CitSPS2*, *CitSUS*)^31,32^, sucrose cleavage directional genes (*CSCW1*, *CUAI1*)^33,34^, acid invertase gene (*CitAI*)^35^, and neutral invertase gene (*NIN*)^36^. Although there are many studies regarding sugar metabolism, enzyme activity, and gene expression in *Citrus* fruits, the molecular mechanism underlying the reduction in fruit sugar content in *K*. *tangor* grafted onto Cj rootstock has rarely been elucidated. To investigate the cause of this reduction, we compared *K*. *tangor* fruit of Cj with that of Pt and analysed the effect of Cj rootstock on sugar accumulation in *K*. *tangor* fruit at the physiological and molecular levels in the present study.

## Results

### Investigation of the HPLC method

There was a strong linear relationship between the concentration of each standard solution and the peak area; the correlation coefficient was 0.9996–0.9998, which indicates high accuracy (Table 1). The linearity range of the standard curve was 0.04–5 mg · mL^−1^, which satisfied the detection and analysis of conditions of each sugar component.

**Table 1:** Regression equations, correlation coefficients, and linearity range of sugar components.

Sugar component	Regression equations (mg · mL^−1^)	Linearity range (mg · mL^−1^)	Correlation coefficients
Sucrose	y = 183447x +29368	0.04~5	0.9998
Fructose	y = 173173x +1064.6	0.04~5	0.9996
Glucose	y = 189541x +7625.5	0.04~5	0.9996

The RSD values of each standard solution were 0.72–3.12% (Table 2). The RSD values of the three carbohydrates ranged from 0.9492% to 3.03%. The RSD values of the sugar content in the solution at different times were less than 2.26%, indicating that the solution was stable within 72 h. The average recoveries were 99.65–109.88% and RSD values were less than 2.90% (Table 2). The results showed that the method had high precision, repeatability, stability, and percent recovery.

**Table 2:** Precision, repeatability, stability, and percent recovery RSD of this method. RSD (%) = relative standard deviation.

Sugar component	Precision %	Repeatability %	Stability %	Percent recovery %
Sucrose	0.7155	2.0058	2.2624	102.3663
Fructose	3.1155	0.9492	1.5724	99.6536
Glucose	2.0498	3.0342	1.1107	109.8767

**Figure 1:**
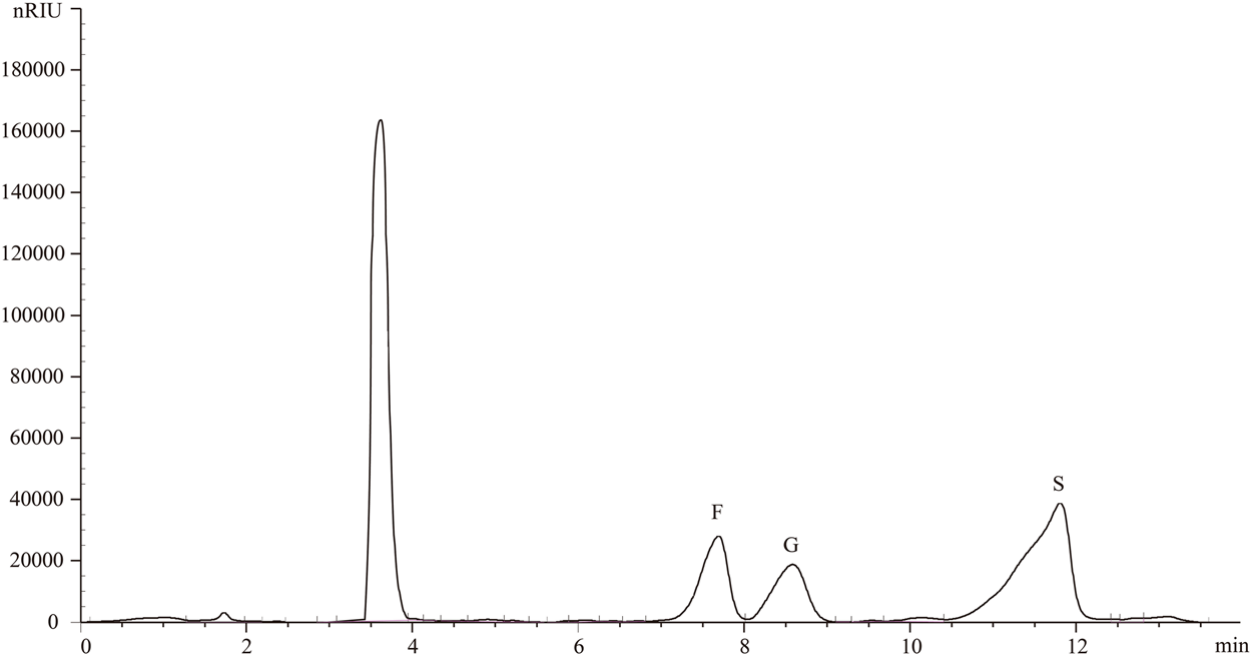
High Performance Liquid Chromatography detection of sugar components in the standard sample. Fructose, glucose, and sucrose followed the peak sequence

In the present study, a HPLC method was developed to analyse the content of the sugar components. The chromatograms of sucrose, fructose, and glucose standards are shown in Fig. 1. Based on the peak type and retention time, the three sugar components were adequately separated within 14 min.

### Effect of different rootstocks on sugar and acid content in *K*. *tangor* fruit

**Figure 2:**
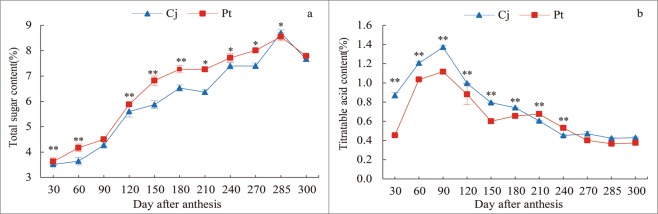
Sugar and acid content in *K*. *tangor* fruits of different rootstocks. (**a**) Total sugar content, (**b**) Titratable acid content. The vertical bars represent standard error. *Shows significant differences (p < 0.05) and **shows extremely significant differences (p < 0.01) between Pt and Cj.

Different rootstocks significantly influenced *K*. *tangor* sugar content. The total sugar(TS) content in *K*. *tangor* fruit showed regular changes during growth and development, increasing continuously at 30–285 days after anthesis and decreasing slightly at 300 days after anthesis. The TS of Cj was always lower than that of Pt prior to 285 days after anthesis. The titratable acid(TA) content increased at 30–90 days after anthesis and decreased slowly at 90 days after anthesis. Additionally, prior to 210 days after anthesis, the TA content of Cj was always higher than that of Pt. However, there was no significant difference in TA content in fruits at the ripening stage (270–300 days after anthesis) (Fig. 2).

### Effect of different rootstocks on the sugar components in *K*. *tangor* fruit

**Figure 3:**
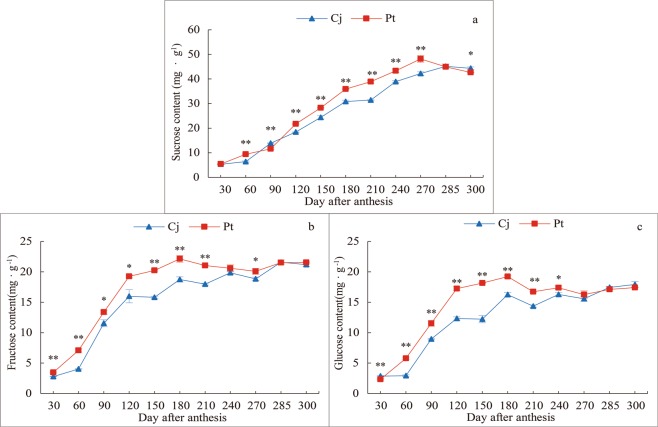
Content of sugar components in *K*. *tangor* fruits of different rootstocks. (**a**) Sucrose content, (**b**) Fructose content, (**c**) Glucose content. The vertical bars represent standard error. *Shows significant differences (p < 0.05) and **shows extremely significant differences (p < 0.01) between Pt and Cj.

The results showed that the sugars in *K*. *tangor* fruit mainly consist of the accumulated sucrose type and that the sucrose content was more than two times higher than the fructose and glucose content in the later stage of fruit development. Additionally, during fruit growth and development, the sugar components showed an increasing trend. The sucrose content in both rootstocks increased steadily before reaching 270 days after-anthesis (Fig. 3a). The sucrose content of Pt was always higher than that of Cj after reaching 120 days after-anthesis (Fig. 3a). Fructose content increased initially, and then remained stable after reaching 180 days after anthesis (Fig. 3b). The glucose content increased rapidly at first, increased slowly from 120 to 180 days after anthesis, decreased slightly from 180 to 210 days after anthesis, and then remained nearly unchanged (Fig. 3c). The fructose and glucose content of Pt was always higher than that of Cj (Fig. 3b,c).

### Effect of different rootstocks on sucrose-metabolism enzyme activities in *K*. *tangor* fruit

**Figure 4:**
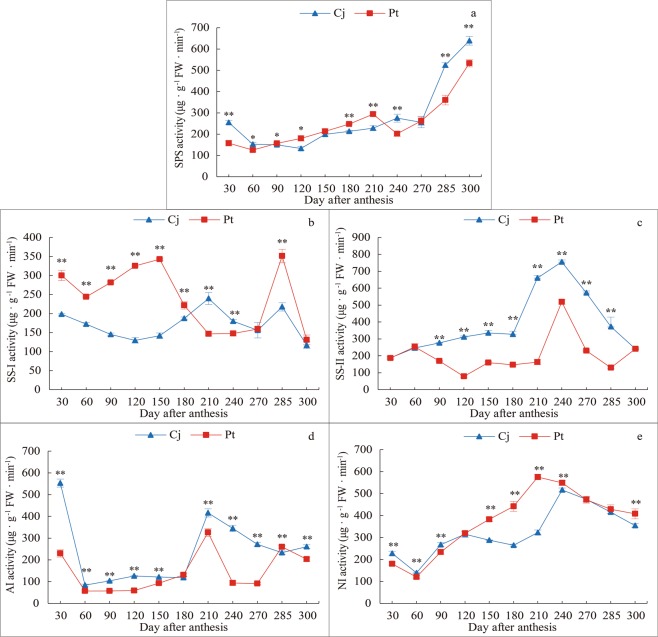
Sucrose metabolism enzyme activities in *K*. *tangor* fruit of different rootstocks. (**a**) SPS activity, (**b**) SS-I activity, (**c**) SS-II activity, (**d**) AI activity, (**e**) NI activity.

The SPS activity remained essentially unchanged before fruit ripening and increased rapidly after reaching 270 days after anthesis. The SPS activity of Pt was initially lower than that of Cj, higher than that of Cj at 90 to 210 days after anthesis, then lower than that of Cj at 240 to 300 days after anthesis (Fig. 4a). The change regularity of SS-I activity in the two rootstocks differed. For example, 30–180 days after anthesis, the SS-I activity of Pt was significantly higher than that of Cj (Fig. 4b). The SS-II activity changed regularly throughout fruit growth and development. The SS-II activity increased slowly and then rapidly, reached its maximum at 240 days after anthesis, and then decreased (Fig. 4c). The SS-II activity of Pt was always lower than that of Cj, except at 30–60 and 300 days after anthesis (Fig. 4c). The AI activity increased rapidly at 180 days after anthesis and then decreased slowly at 210 days (Fig. 4d). The AI activity of Pt was always lower than that of Cj, except at 285 days after anthesis (Fig. 4d). The NI activity of Pt reached its maximum at 210 days after anthesis, whereas that in Cj reached its maximum at 240 days after anthesis. The NI activity then decreased slowly (240–300 days after anthesis), and the NI activity of Pt was higher than that of Cj except 30–120 days after anthesis (Fig. 4e).

### Effect of different rootstocks on the expression of sucrose metabolism-related genes

**Figure 5:**
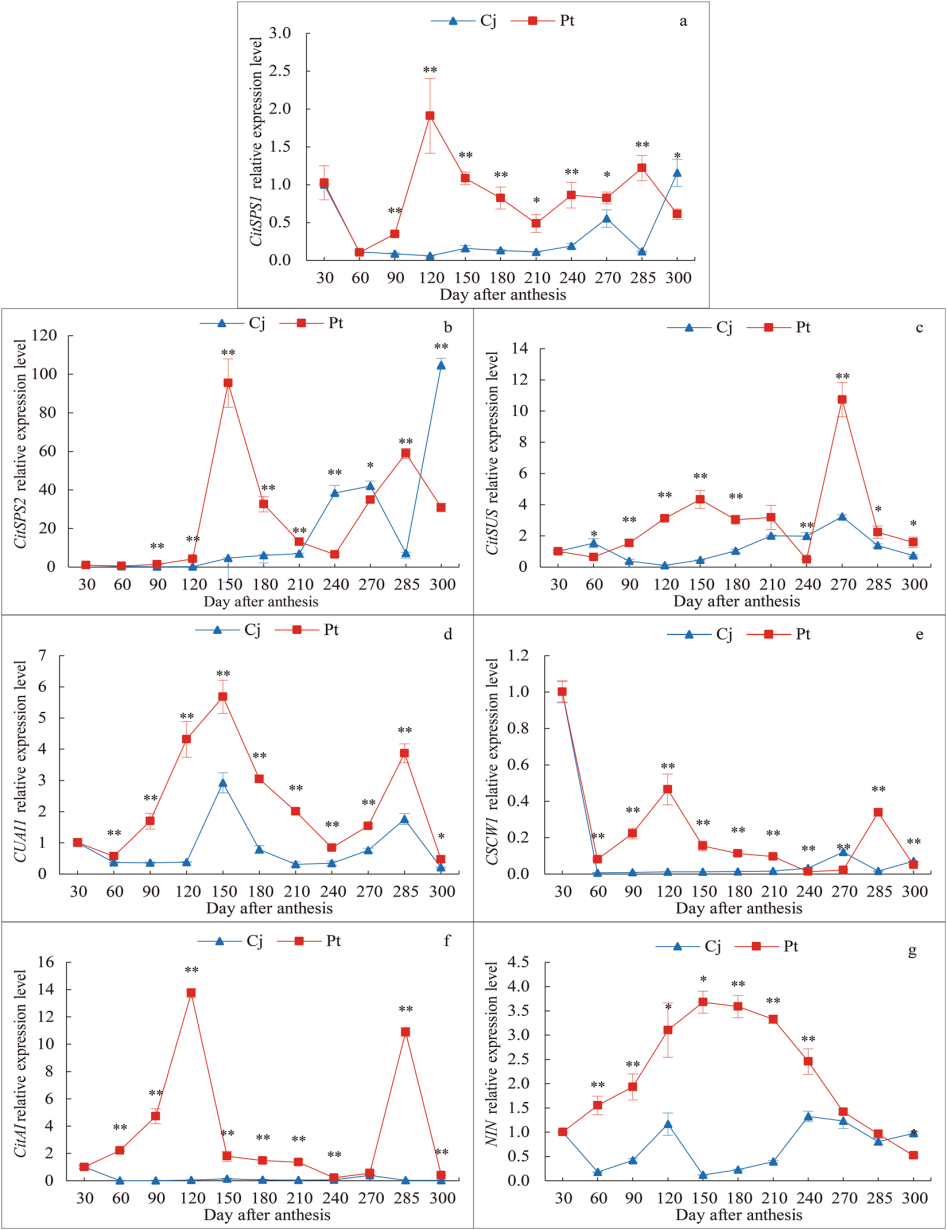
Real-time fluorescence quantitative PCR analysis of the effect of different rootstocks on the relative expression of sucrose metabolism enzyme genes during the growth period of *K*. *tangor*. (**a**) *CitSPS1* relative expression level, (**b**) *CitSPS2* relative expression level, (**c**) *CitSUS* relative expression level, (**d**) *CUAI1* relative expression level, (**e**) *CSCW1* relative expression level, (**f**) *CitAI* relative expression level, (**g**) *NIN* relative expression level.

The expression of the *CitSPS1* gene in Pt decreased to the lowest level at 60 days after anthesis and then increased to the highest level at 120 days after anthesis. The expression of the *CitSPS1* gene in Pt was higher than that in Cj at all times, except at 30–60 and 300 days after anthesis (Fig. 5a). The *CitSPS2* gene of Pt and Cj was almost not expressed until 120 days after anthesis and the maximum expression occurred at 150 days after anthesis in Pt. The *CitSPS2* gene expression in Pt was significantly higher than that in Cj at 150–180 days after anthesis, but its expression in Pt and Cj showed an opposite trend at 270–300 days after anthesis (Fig. 5b). The expression of the *CitSUS* in Cj was low throughout the fruit growth and development processes, and the highest expression occurred at 270 days after anthesis. The expression of this gene in Pt was significantly higher than that in Cj, except at 30–60 and 240 days after anthesis (Fig. 5c). The expression of *CUAI1* showed an “M” pattern, reaching its peak at 150 and 285 days after anthesis, and its expression in Pt was significantly higher than that in Cj (Fig. 5d). The *CSCW1* gene expression in Pt and Cj was the highest at 30 days after anthesis and was then expressed at much lower levels in both rootstocks. Moreover, the *CSCW1* gene expression in Pt was always significantly higher than that in Cj except at 270 days after anthesis (Fig. 5e). *CitAI* expression in Pt reached the maximum at 120 days after anthesis and reached the second peak at 285 days after anthesis. The expression of this gene in Cj was very low throughout the fruit growth and development processes (Fig. 5f). *NIN* expression in Pt initially increased and then decreased, with the highest and lowest expression at 150 and 300 days after anthesis, respectively. Additionally, *NIN* expression in Pt was higher than that in Cj at 60 to 285 days after anthesis (Fig. 5g).

### Correlation analysis of each index

**Figure 6:**
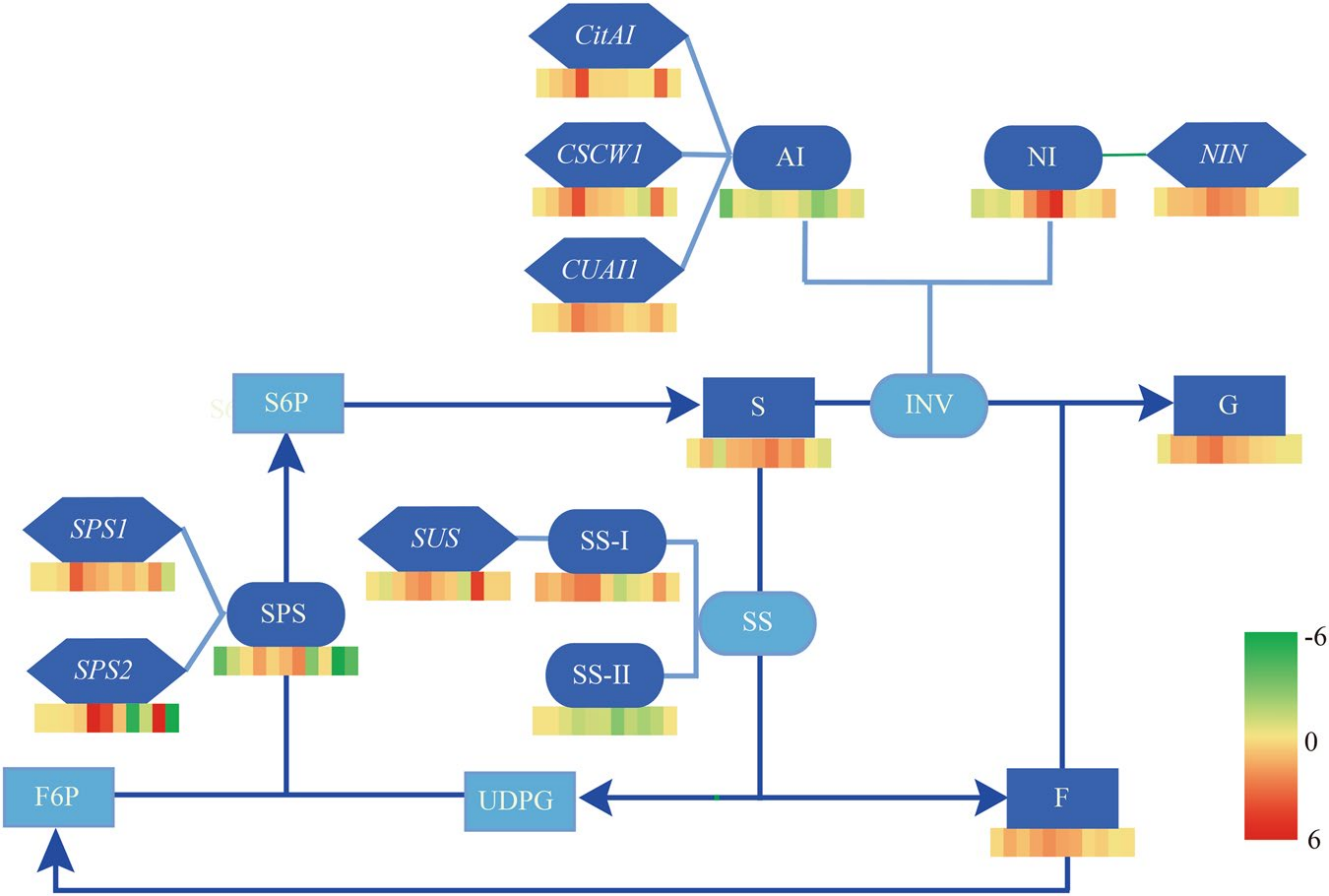
Difference in sugar accumulation between *K*. *tangor* fruits with Pt and Cj as rootstock during fruit growth and maturation. Different colours represent different values of $\frac{{X}_{Pt}-{X}_{Cj}}{|\overline{{X}_{Pt}-{X}_{Cj}}|}$, and higher values are redder and lower are greener. F presents fructose, G is glucose, and S is sucrose.

Correlation analysis of each index is summarised in Table 3. There was an extremely significant positive correlation between total sugar, sugar components, and SPS activity, whereas titratable acid showed an extremely significant negative correlation with total sugar, sugar components, SPS and NI activities, and *CitSPS2* gene expression. The NI activity was extremely significantly positively correlated with the total sugar (r = 0.84) and sugar components (r = 0.86, 0.81, 0.79). *CitSPS2* expression was positively correlated with the total sugar (r = 0.54), sugar components (r = 0.54, 0.50, 0.53), and SPS activity (r = 0.56). *CSCW1* expression was negatively correlated with the total sugar (r = −0.47) and sugar components (r = −0.48, −0.56, −0.52), and significant positively correlated with the SS-I activity (r = 0.46) and *CitSPS1* expression (r = 0.54). *CitSPS1* expression was positively correlated with the SS-I activity (r = 0.47) and *NIN* expression (r = 0.51), but significantly negatively correlated with the SS-II activity (r = −0.49). *CUAI1* expression was extremely significantly positively correlated with the SS-I activity (r = 0.67), and *NIN* (r = 0.63) and *CitSPS1* (r = 0.56) expression, but negatively correlated with the SS-II activity (r = −0.50). *CitAI* expression was significant positively correlated with the SS-I activity (r = 0.69), *CitSPS1* (r = 0.65) and *CUAI1* (r = 0.61) expression, but negatively correlated with the SS-II activity (r = −0.47). These correlations can also be visualized in Fig. 6.

**Table 3:** Relevance analysis.

	Total sugar	Titratable acid	Sucrose	Fructose	Glucose	SPS	SS-I	SS-II	AI	NI	*NIN*	*CitSPS1*	*CitSPS2*	*CUAI1*	*CitSUS*	*CSCW1*	*CitAI*
Total sugar																	
Titratable acid	−0.77**																
Sucrose	0.98**	−0.77**															
Fructose	0.93**	−0.58**	0.90**														
Glucose	0.90**	−0.56**	0.87**	0.99**													
SPS	0.64**	−0.63**	0.65**	0.50*	0.49*												
SS-I	−0.12	−0.05	−0.23	−0.09	−0.04	−0.24											
SS-II	0.24	−0.24	0.30	0.18	0.15	0.00	−0.36										
AI	0.07	−0.36	0.12	−0.07	−0.10	0.35	−0.10	0.33									
NI	0.84**	−0.64**	0.86**	0.81**	0.79**	0.39	−0.23	0.31	0.20								
*NIN*	0.20	−0.09	0.14	0.34	0.40	−0.15	0.37	−0.33	−0.20	0.41							
*CitSPS1*	0.17	−0.36	0.15	0.17	0.24	0.19	0.47*	−0.49*	0.03	0.19	0.51*						
*CitSPS2*	0.54**	−0.54*	0.54*	0.50*	0.53*	0.560**	0.11	−0.07	0.09	0.39	0.28	0.43*					
*CUAI1*	0.23	−0.13	0.11	0.32	0.37	−0.08	0.67**	−0.50*	−0.25	0.18	0.63**	0.56**	0.34				
*CitSUS*	0.37	−0.35	0.40	0.32	0.33	0.02	0.11	−0.14	−0.11	0.40	0.33	0.30	0.30	0.33			
*CSCW1*	−0.47*	−0.05	−0.48*	−0.56**	−0.52*	−0.14	0.46*	−0.38	0.40	−0.33	0.07	0.54**	−0.14	0.15	−0.09		
*CitAI*	0.05	0.03	−0.04	0.13	0.17	−0.09	0.69**	−0.47*	−0.18	−0.02	0.33	0.65**	0.05	0.61**	0.11	0.31

## Discussion

There are several methods to determine sugar components by high-performance liquid chromatography globally^37^. We optimised the HPLC method for the determination of sugar components in *Citrus*, and the Innoval NH_2_ column used in this analysis has higher application value. The stability, precision, repeatability, and recovery rate of the method used were high (Tables 1 and 2), and this method can therefore be used to effectively determine the content of sugar components in *Citrus* fruits.

As mentioned previously, sugars determine fruit flavour^38^. This study showed that different rootstocks had a significant effect on sugar accumulation in *K*. *tangor* fruit. This was consistent with the results of Killiny *et al*.^39^. Organic acids play an important role in the flavour and pH of fruit and affect the sensorial quality of fruits^40^. The sugar content in Cj rootstock was significantly lower than that of Pt rootstock, but the difference in acid content was small. Therefore, we focused on the exploration of glucose metabolism. Moreover, there were significant differences in sugar component content, as well as sucrose metabolism enzyme activity and gene expression levels between Cj and Pt rootstocks (Fig. 6). However, the sugar contents of the two rootstocks were essentially the same at 300 days after anthesis (the ripening stage) (Fig. 2).

Previous studies have reported that the total sugar content and composition are closely related to fruit quality^41^. Sucrose, glucose, and fructose are the major nutritional components of fleshy fruits^42^. Many studies found that sucrose, which was transported through sieve elements and then directly enters into the sink organs through the plasmodesmata or apoplastic space, is the main component for sweetness^43,44^. In accordance with the present study results, sugars in most *Citrus* fruits, including *K*. *tangor*, are of the sucrose accumulation type^18^, except in some acidic fruits such as lemons^45^. In this study, sucrose content was the highest, followed by fructose content in the riped *K*. *tangor* fruit. Moreover, during fruit growth and development, sucrose was accumulated continuously. However, the content of fructose and glucose changed slightly in the later stages of fruit development (Fig. 3), although they also increased continuously in a previous study^46^. This just showed that different rootstocks (Cj and Pt) had an important effect on the accumulation of sugar components in *K*. *tangor* fruit, especially in the early stage of sugar accumulation.

According to previous studies, sucrose and its main components, glucose, and fructose, are some of the most important sugars in the process of carbon assimilation^47^. The sugar content in *K*. *tangor* with Pt as a rootstock was higher than that in *K*. *tangor* with Cj, but there was no significant difference at maturity. Studies have shown that *Citrus* fructose has the strongest flavour among the sugar components, followed by sucrose^48^. In this study, the content of sucrose and fructose in the fruits of the two rootstocks was significantly different in the later stages of fruit growth and development (240–270 days after anthesis), whereas the content of glucose was only slightly different (Fig. 3), which may be why *K*. *tangor* fruits with Ziyang xiangcheng rootstock have a light flavour. Another study found that rootstock regulates sugar metabolism mainly by affecting the content of sucrose and glucose, but there was little effect on fructose content^39^. These results were inconsistent with the conclusion of this study and whether this difference was caused by scion or rootstock remains to be further researched.

Sugar accumulation is an important factor determining fruit quality and sucrose metabolism is important in the regulation of sugar accumulation^49–51^. Furthermore, *Citrus* SPS plays an important role in sucrose biosynthesis^29^. SS, a sugar accumulation enzyme, determines fruit quality^32^. The correlation analysis showed that SPS was extremely significantly positively correlated with fruit sugar content. This result coincides with the study of Zhang *et al*.^52^. It was because SPS increased the substrate for sucrose synthesis by producing sucrose-6-phosphate, which in turn increases the content of sucrose. We also found that the SS-II activity of Cj was always higher than that of Pt. Moreover, the SS-I activity of Pt was higher than that of Cj during the early growth and maturation stages, but it was opposite during the fruit discolouration period. SS-II caused sucrose to break down, and SS-I controls the synthesis of sucrose. Moreover, the NI activity increased with the increase in sugar content (Fig. 4), and this phenomenon was consistent with the results of a study on grapes^53^. The increase of NI activity promoted the production of fructose and glucose. In addition, sucrose content decreased after fruit ripening, while fructose and glucose content increased. This suggested that invertase catalysed the irreversible hydrolysis of sucrose into glucose and fructose to maintain the balance of sucrose-sink during fruit growth and development. This further explained the difference in sugar content between the two rootstocks at the level of enzyme activity.

Furthermore, sucrose metabolism enzyme genes affect the sugar content in fruit through the regulation of sucrose metabolism enzymes^52,54^ and seven of these genes were quantitatively analysed in this study. The results showed that for genes related to sugar accumulation or degradation, the expression in Pt was higher than that in Cj, and the expression was essentially the same at the maturity stage for the fruit of both rootstocks (Fig. 5). This result revealed why the sugar content in Cj rootstock was always lower than that in Pt rootstock at the molecular level. Furthermore, sugar metabolism-related genes of Pt, in which the sugar content was higher, presented relatively higher expression. These results were consistent with the results of Zhu *et al*.^53^, but differed from the results of Silva^55^ in that the expression levels of *SPS1* showed an opposite pattern. Therefore, most of the sucrose metabolizing enzyme activity of Pt was higher, which made its sugar content always higher than Cj.

Before the fruit ripening stage, the sugar content of Cj rootstock was lower than that of Pt and the fruit flavour was weak. This was due to the regulation of sucrose metabolism-related enzymes and gene expression. However, 15 to 30 days later, Cj rootstock increased the expression of sucrose synthesis orientation genes (*CitSPS1*, *CitSPS2*), reduced the expression of sucrose decomposition orientation genes (*CSCW1*, *CUAI1*), increased the activity of SPS and AI, and reduced the activity of SS-I (the direction of sucrose cleavage) in *K*. *tangor* fruit. These results therefore indicate that there is no significant difference in the sugar content in *K*. *tangor* fruit between the two rootstocks at 30 days after fruit ripening (300 days after anthesis).

## Materials and Methods

### Plant materials

Five-year-old *K*. *tangor* trees (trifoliate orange and Ziyang xiangcheng were the rootstocks), planted in the Shehong Agricultural Science and Technology Park, were used in this study. There were six *K*. *tangor* trees for each of the two rootstocks. All trees were robust, moderately managed, free of pests and diseases, and spaced 3 m × 4 m apart.

Ten *K*. *tangor* fruits from each tree of the two rootstocks were randomly picked every 30 days from 30 to 270 days after anthesis. The fruit was sampled every 15 days after ripening (285 and 300 days after anthesis, respectively). *Kiyomi tangor* fruit peels were then quickly removed and the pulp was cut into small pieces, mixed, and immediately placed on dry ice. Some pulp was taken to the laboratory, and then immediately frozen in liquid nitrogen and stored at −80 °C to extract sucrose metabolism enzymes and RNA, and the remaining pulp was placed in a refrigerator at −20 °C to determine sugar and acid content.

### Total sugar and titratable acid content

TS content in ripe fruit was estimated using the anthrone–sulphuric acid method with minor modifications^56^, while TA content was determined using the He’s^57^ method with slightly modification.

### Extraction of sugar components

Sugar components were extracted according to the method described by Zhang *et al*.^52^. Two grams of ground *K*. *tangor* fruit flesh was mixed with 4 mL of H_2_O and placed in a water bath at 80 °C for 15 min. The sample was then centrifuged at 4 °C for 15 min at 9000 rpm. We then centrifuged the residue again under the same conditions. The resulting supernatant was combined and the volume was fixed to 10 mL. The sample solution was extracted with a disposable syringe and filtered using a 0.45 µm hydrophilic membrane into a sample bottle, and then stored at 4 °C until analysis.

### Chromatography conditions and verification of HPLC methodology

Sugar (glucose, fructose, and sucrose) content was analysed by high-performance liquid chromatography (HPLC) (LC-1260; Agilent Technologies, Sacramento, CA, USA) according to the method of Zhang *et al*.^52^, but we optimised the chromatographic conditions. Samples were isolated on the Innoval NH_2_ column (4.6 mm × 250 mm, 5 µm, Agela Technologies, Tianjin, China) at room temperature. The HPLC experiment was completed using a mobile phase (acetonitrile:water = 80:20 (V/V)), 20 μL sample volume, 1 mL · min^−1^ flow rate, 30 °C column temperature, and 40 °C detection temperature.

The feasibility of the HPLC method was verified by linear relationship, precision, repeatability, stability, and recovery. The precision, repeatability, stability, and recovery of the samples were tested with reference to the method described by Huang^58^ to analyse the reliability of the treatment method and the chromatographic conditions to determine sugar content in *K*. *tangor* fruit. The mixed standard solution (2 mg · mL^−1^) was prepared and sample injection was repeated six times. The standard deviation (RSD) of the standard solution was calculated according to the peak area of each standard sample. The six identical samples were then weighed, the content of sugar components in each sample was calculated according to the peak area, and the RSD values were then calculated. The same sample was then injected at 0, 2, 4, 6, 8, 12, 24, 36, 48, and 72 h, respectively, and the RSD values of the sugar content were calculated for the different times. Next, the six samples with known content of sugars were weighed again and a certain amount of the mixed standard solution was added to carry out the recovery experiment.

### Determination of sucrose metabolism enzyme activity

Sucrose metabolism enzyme was prepared from frozen tissues of *K*. *tangor* as described by Zhang^44^. SPS, SS-I, SS-II, AI, and NI activities were assayed using the Plant SPS, SS-I, SS-II, AI, and NI ELISA test kits (Shanghai BOYE Biology Science and Technology Co. Ltd., Shanghai, China) according to the manufacturer’s instructions.

### Quantitative real-time polymerase chain reaction analysis

Total RNA was extracted from *K*. *tangor* fruit of two rootstocks using RNAiso Plus (TaKaRa, Dalian, China). First-strand cDNA was synthesised with using the Prime Script RT reagent Kit with gDNA Eraser (Takara, Dalian, China)^26^. Primer 3.0 online tool (http://bioinfo.ut.ee/primer3–0.4.0/) was used to design primers, which were synthesised by Sangon Biotech, China. Primer sequences of the *CitSPS1*, *CitSPS2*, *CUAI1*, *CitSUS*, *CSCW1*, *CitAI*, *NIN*, and *β-actin* gene were listed in Table 4. The quantitative real-time polymerase chain reaction (qRT-PCR) experiment was conducted using SYBR Premix Ex Taq II (Takara, Dalian, China) and the CFX96 Real-Time PCR system (Bio-Rad, California) with three technical replicates^59^. All primers were amplified with no template control to ensure the amplicons were not primer dimers. The relative gene expression levels were calculated using the 2^−ΔΔCT^ method with the *β-actin* gene serving as the internal control^60^.

**Table 4:** Specific primers for quantitative real-time PCR.

Genes	Primer Forward	Primer Reverse
*CitSPS1*	GCTCCCTCCTTATCCTTTCGT	AGCAGCAACAAGACATCGAG
*CitSPS2*	GTTGAACTTGCCCGAGCCTT	ACATCTCGTTCGGTTCACCA
*CUAI1*	ACAGCTTGGTTGACATCCGAA	ATAAAAGTCCACGCACTCCC
*CitSUS*	AGAGTCACACTGCTTTCACTC	GCTCATATCAGCACCAGGAG
*CSCW1*	AATAACATCAACGCTAGCTCA	GCGTTTAGCCTTAACCCAGT
*CitAI*	CCTTTCTTCACATCCGCAAT	GCATTGGTTCCAAGGACTTC
*NIN*	CAGCATTACTCTCTGCACGT	TTCCCTGATATGGAATGACAAAG
*β-actin*	CCAAGCAGCATGAAGATCAA	ATCTGCTGGAAGGTGCTGAG

### Data analysis

The data were analysed with Statistic, version 23.0 of IBM SPSS. Statistically significant differences (p < 0.05) and extremely significant differences (p < 0.01) between the treatments were revealed after independent sample t-test graphs were plotted using Adobe Illustrator and SigmaPlot 12.5.

## Supplementary information

Supplementary Figure
