# Supplementary material for: Investigation of the cause of reduced sugar content in Kiyomi tangor fruit of Ziyang xiangcheng (Citrus junos Sieb. ex Tanaka) rootstock
Source: Sci Rep. 2019 Dec 17;9:19263. doi: 10.1038/s41598-019-55957-3 (PMC6917820; doi:10.1038/s41598-019-55957-3)
Supplement: Supplementary file 1 — Supplementary Figure [file 41598_2019_55957_MOESM1_ESM.doc]

**Investigation of the cause of reduced sugar content in** ***Kiyomi tangor* fruit of Ziyang xiangcheng (*Citrus junos* Sieb. ex Tanaka) rootstock**

Tiantian Dong^1,†^, Bo Xiong^1,†^, Shengjia Huang^1^, Ling Liao^1^, Xia Qiu^1^, Guochao Sun^1,2^, Yunzhenzi He^1^, Changwen Duan^1^, Xiaojia Wang^1^, Xu Zhang^1^, Sichen Li^1^, Jin Zhu^3^ & Zhihui Wang^1,2^

^1^College of Horticulture, Sichuan Agricultural University, Chengdu 611130, Sichuan, China.

^2^Institute of Pomology and Olericulture, Sichuan Agricultural University, Chengdu 611130, Sichuan, China.

^3^Sichuan Horticultural Crop Extension Station, 610041, Sichuan, China.

Correspondence and requests for materials should be addressed to Z.-H.W. (E-mail: [wangzhihui318@126.com](mailto:wangzhihui318@126.com))

^†^ These authors contributed equally to this work.

**Supplementary Figures**

Figure S1.


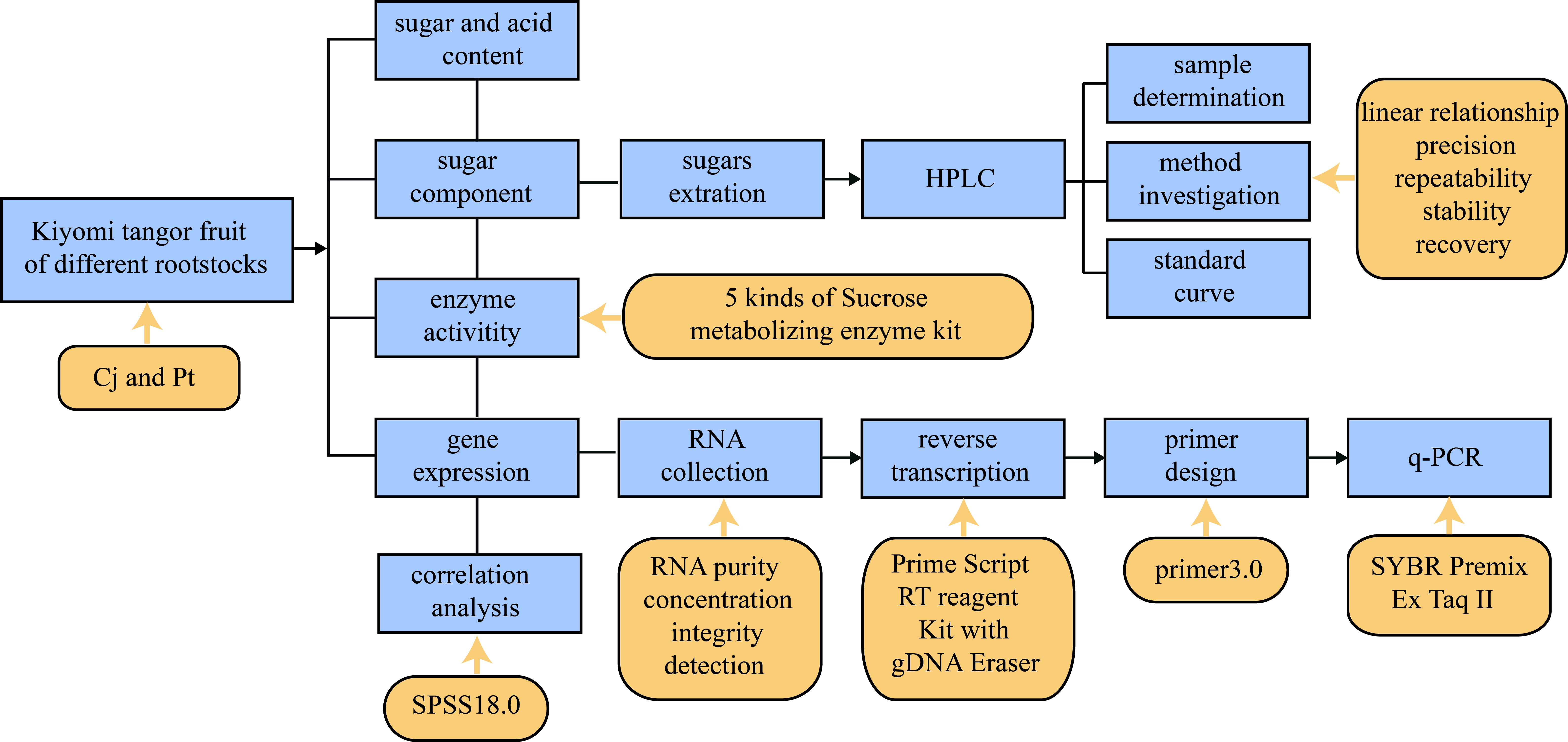


Figure S1. Schematic overview of the study. Using Kiyomi tangor materials, we explored the cause of when Cj was used as a rootstock in K. tangor production, the sugar content of this fruit was lower than that of the fruit using the Poncirus trifoliata (L.) Raf. rootstock (abbreviated Pt.). We used high performance liquid chromatography, spectrophotometry, and qPCR to determine Citrus sugar accumulation, sucrose metabolic enzyme activity, and gene expression. The reliability of determination of sugar components by HPLC was tested with linear relationgship, repeatability, precision, stability and recovery.

Figure S2.


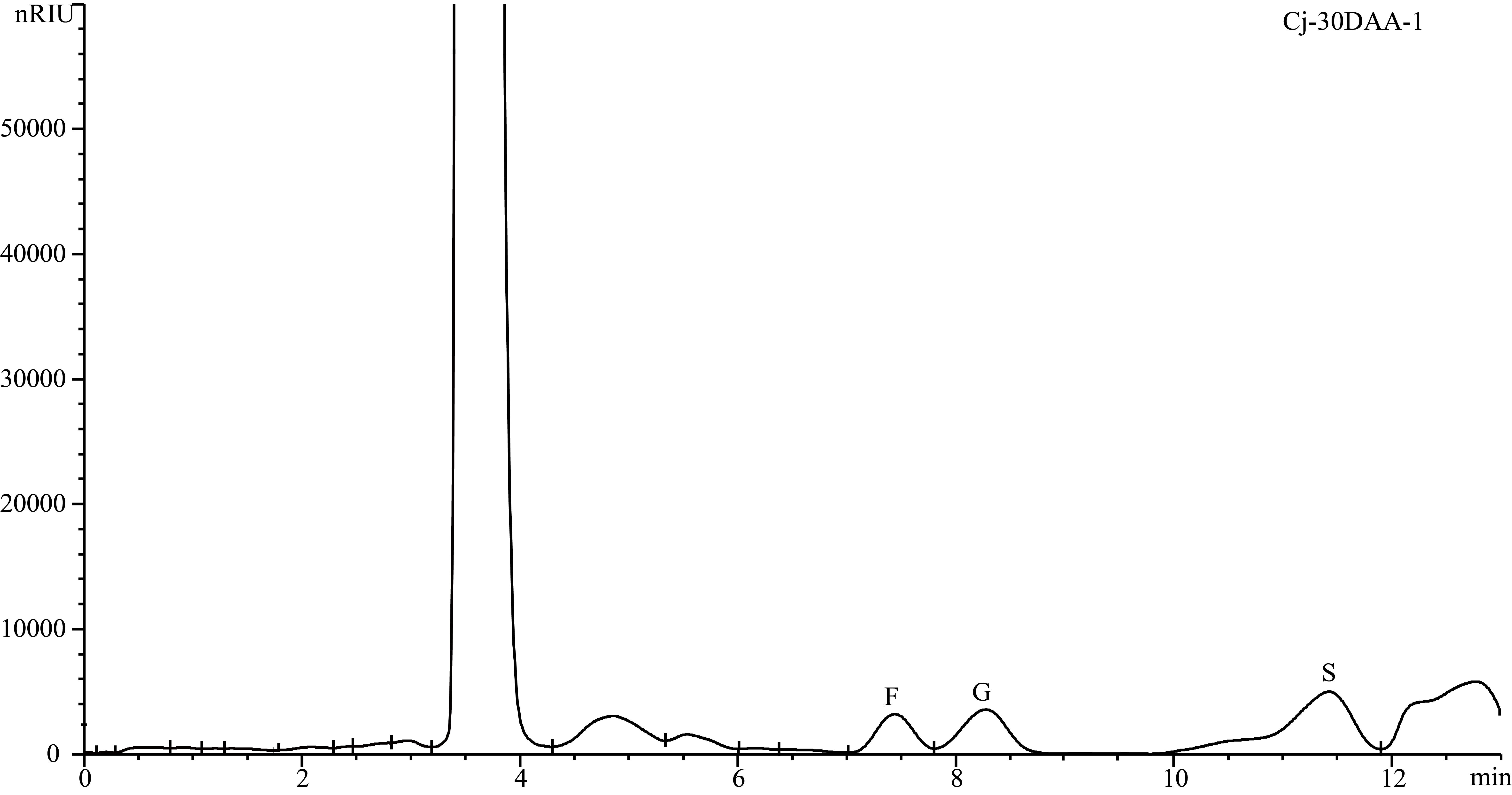

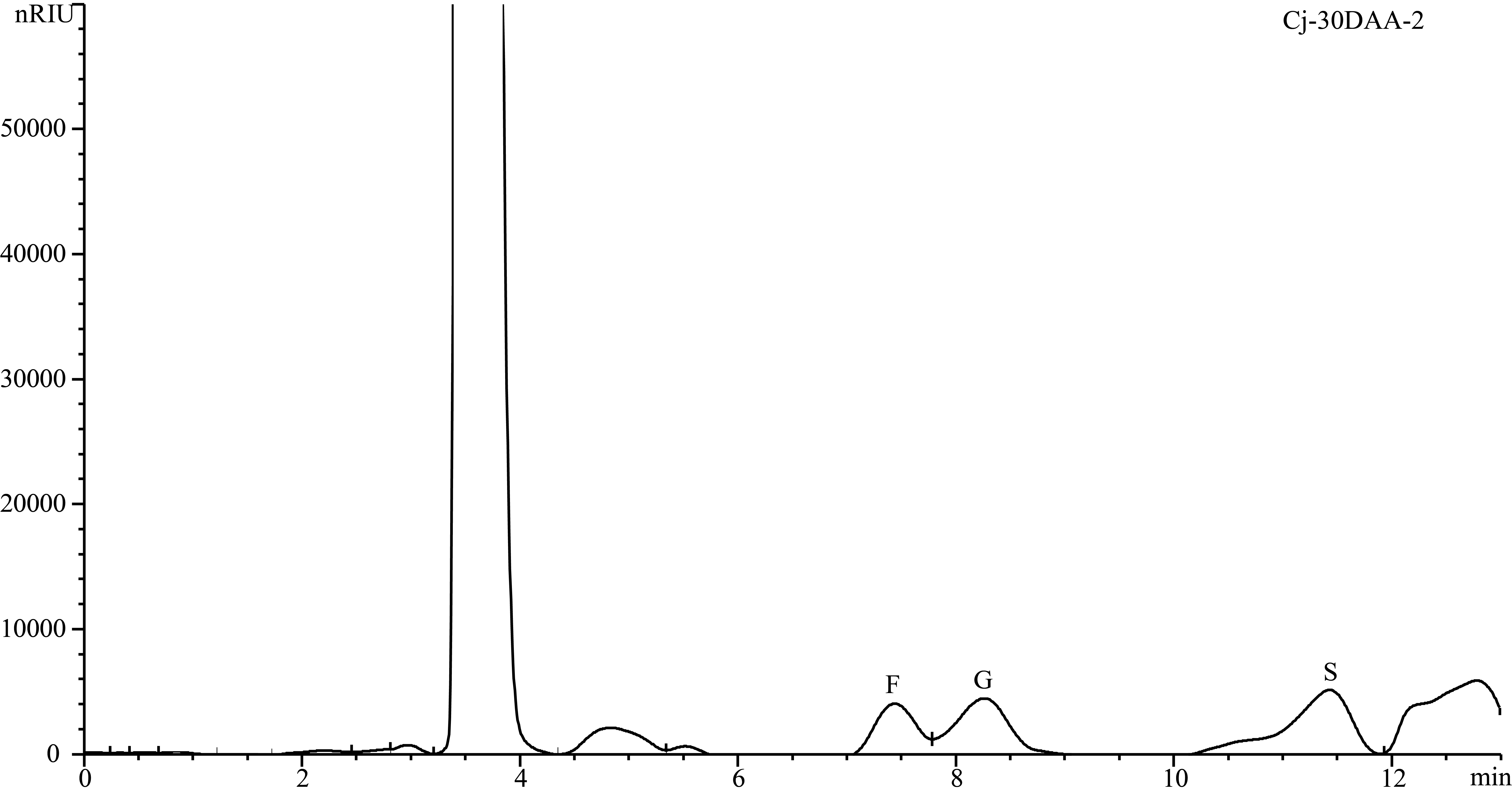

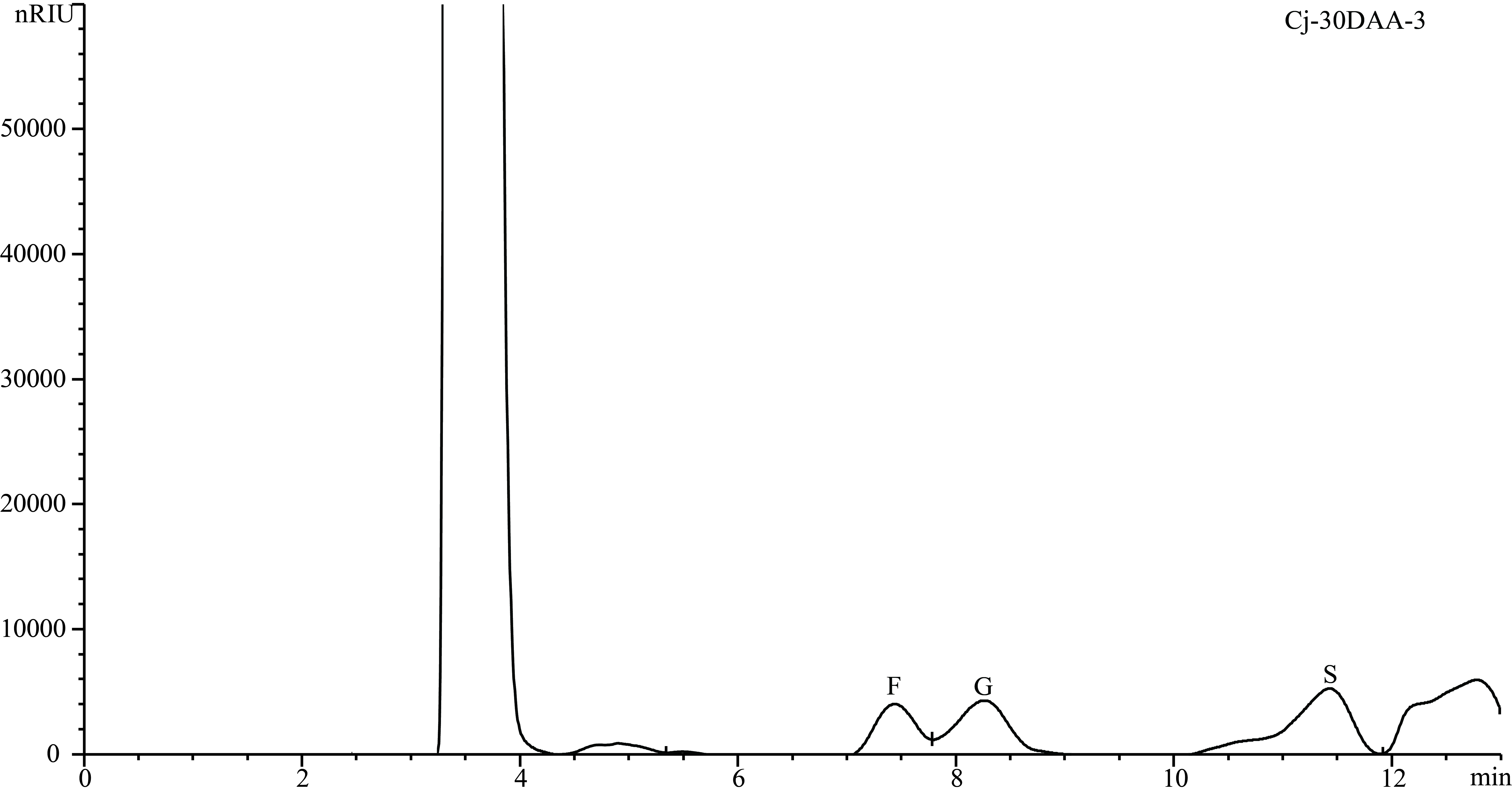


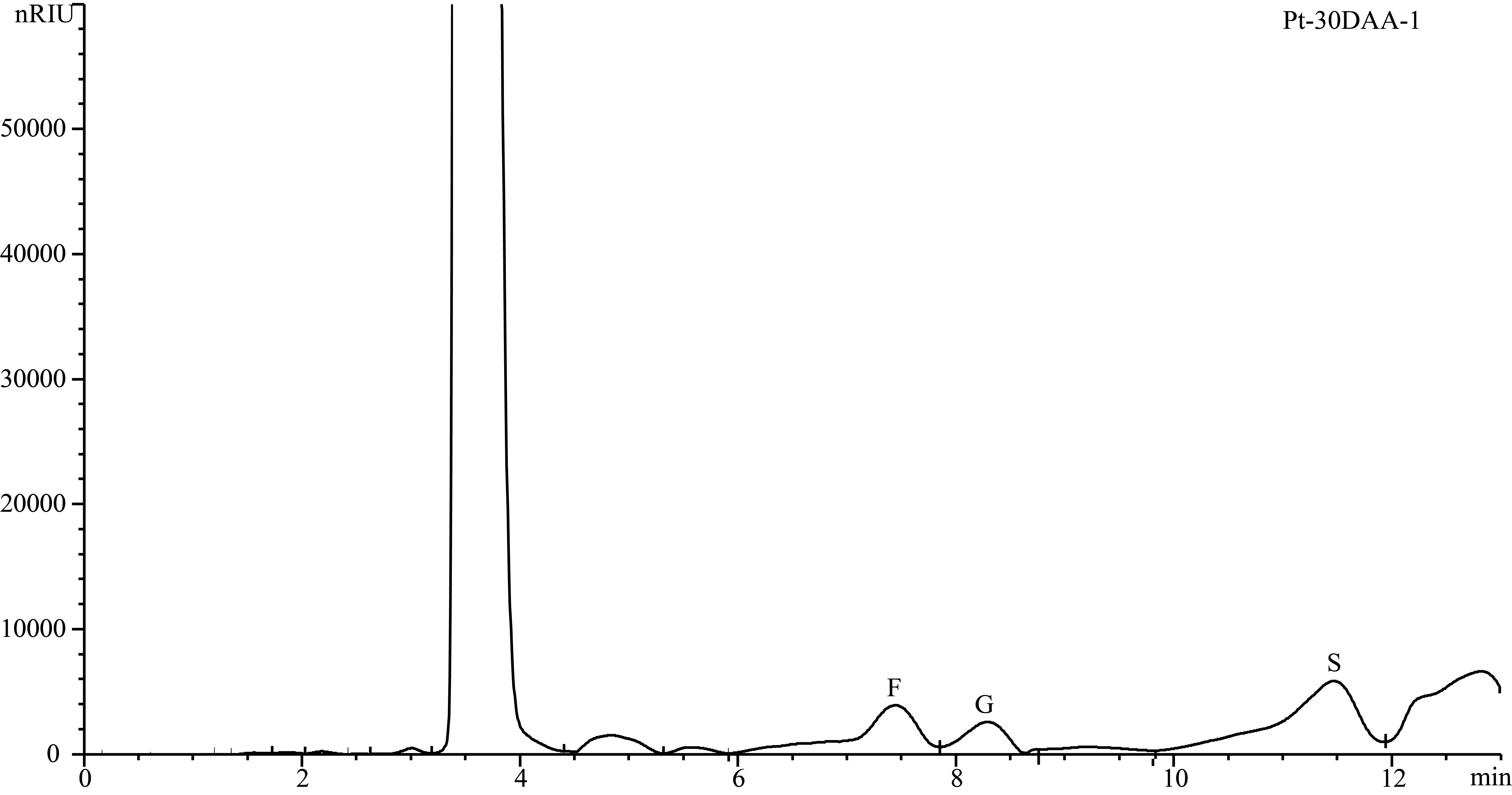

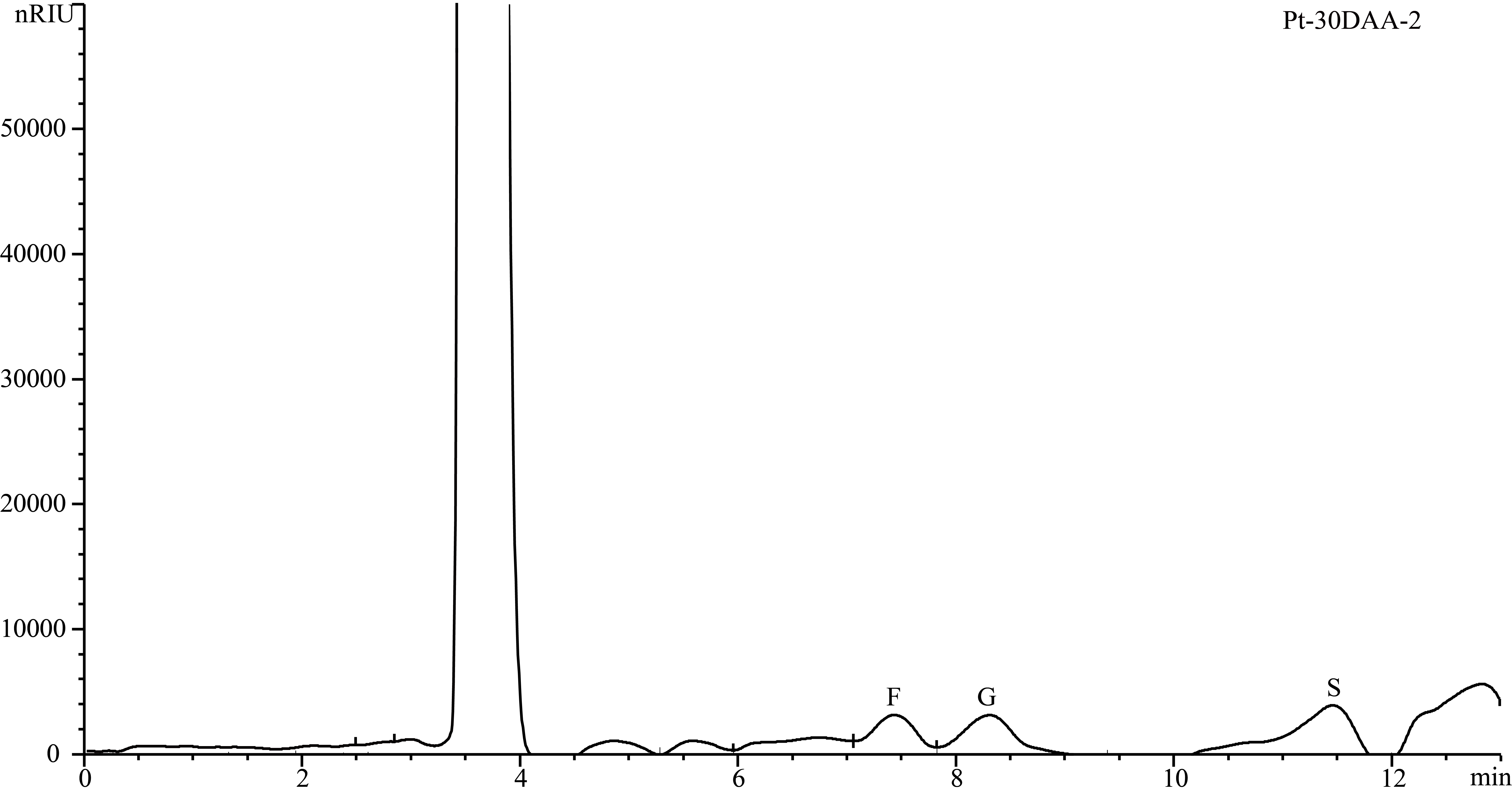

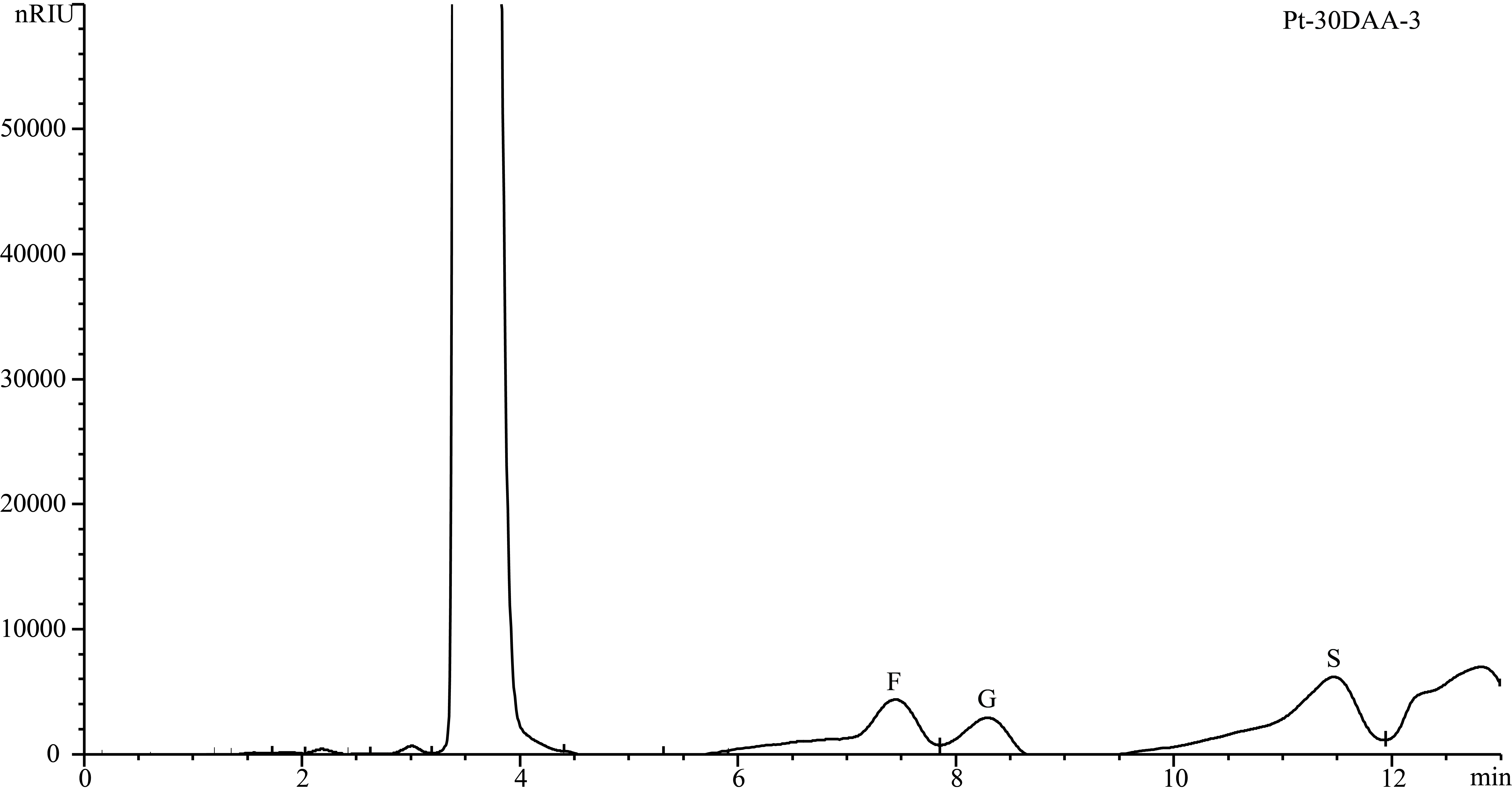


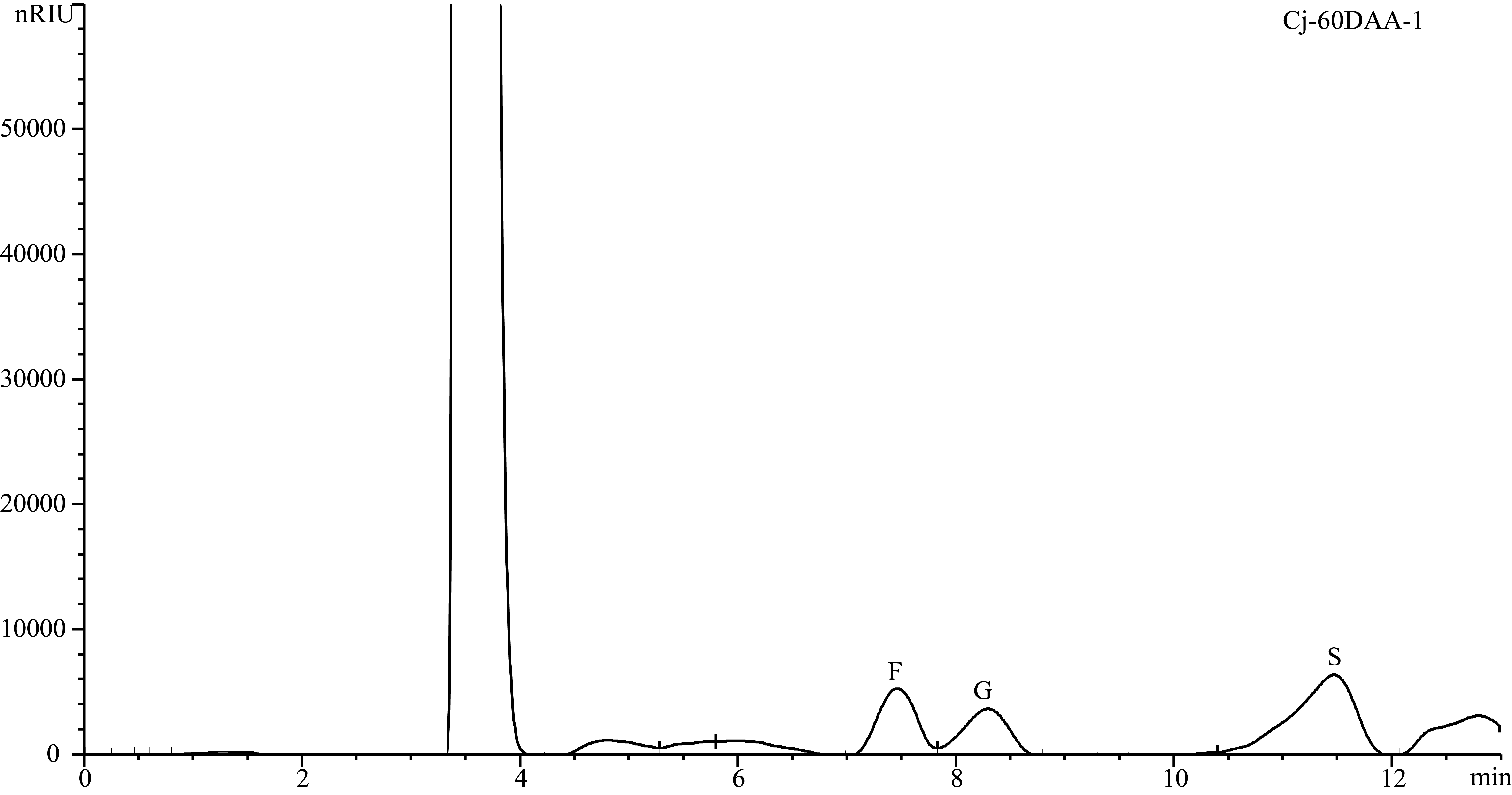

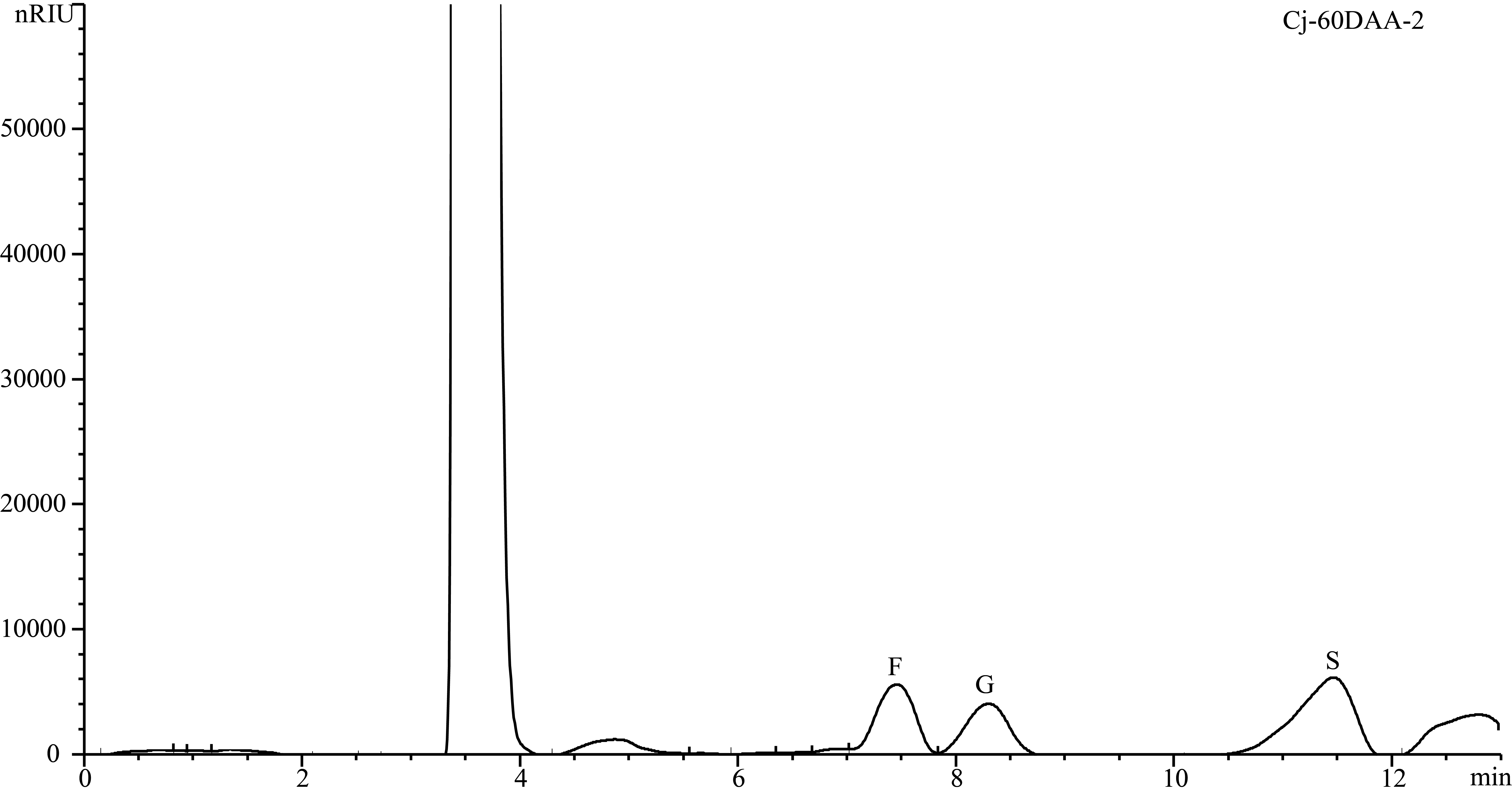

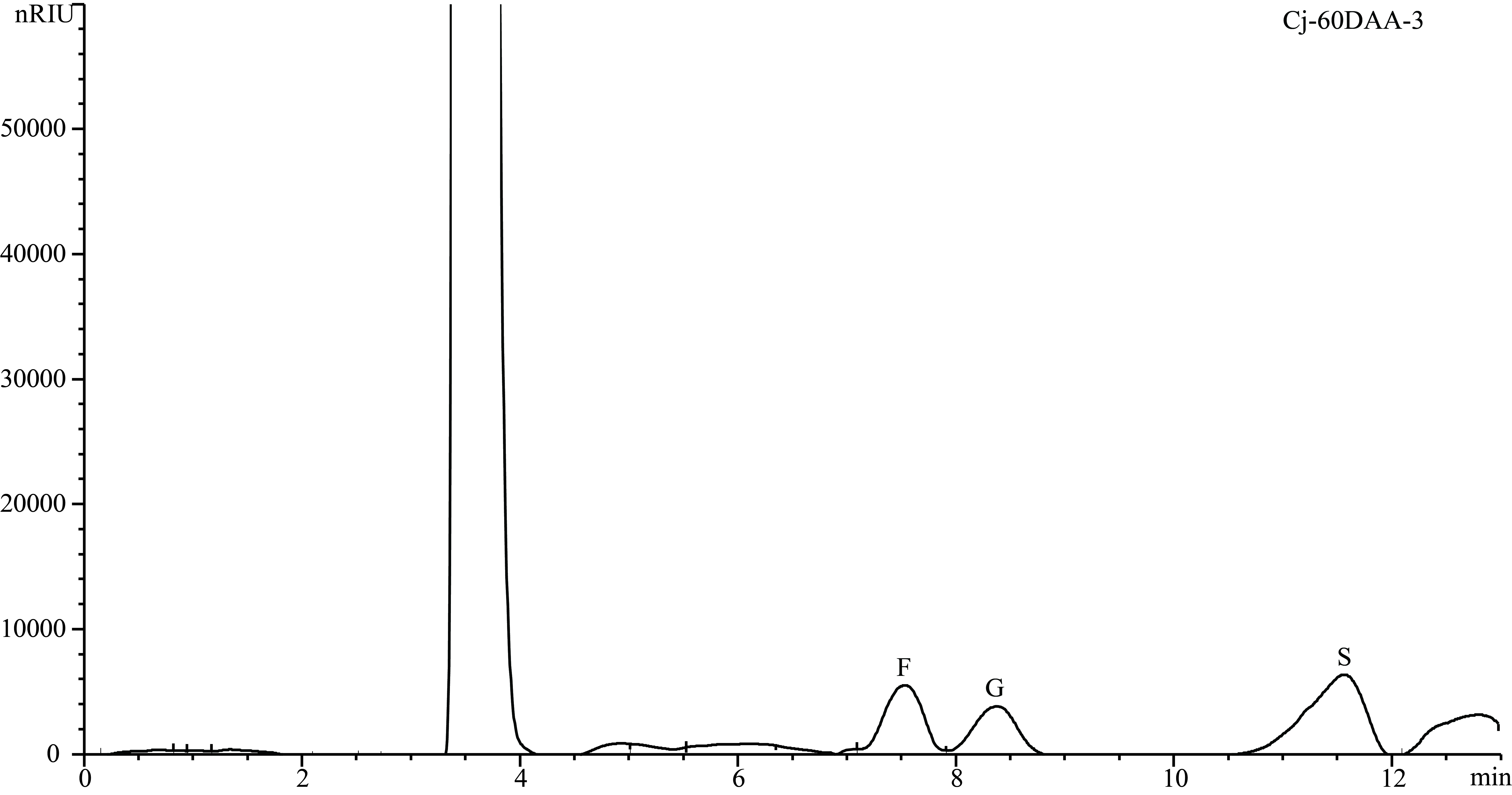


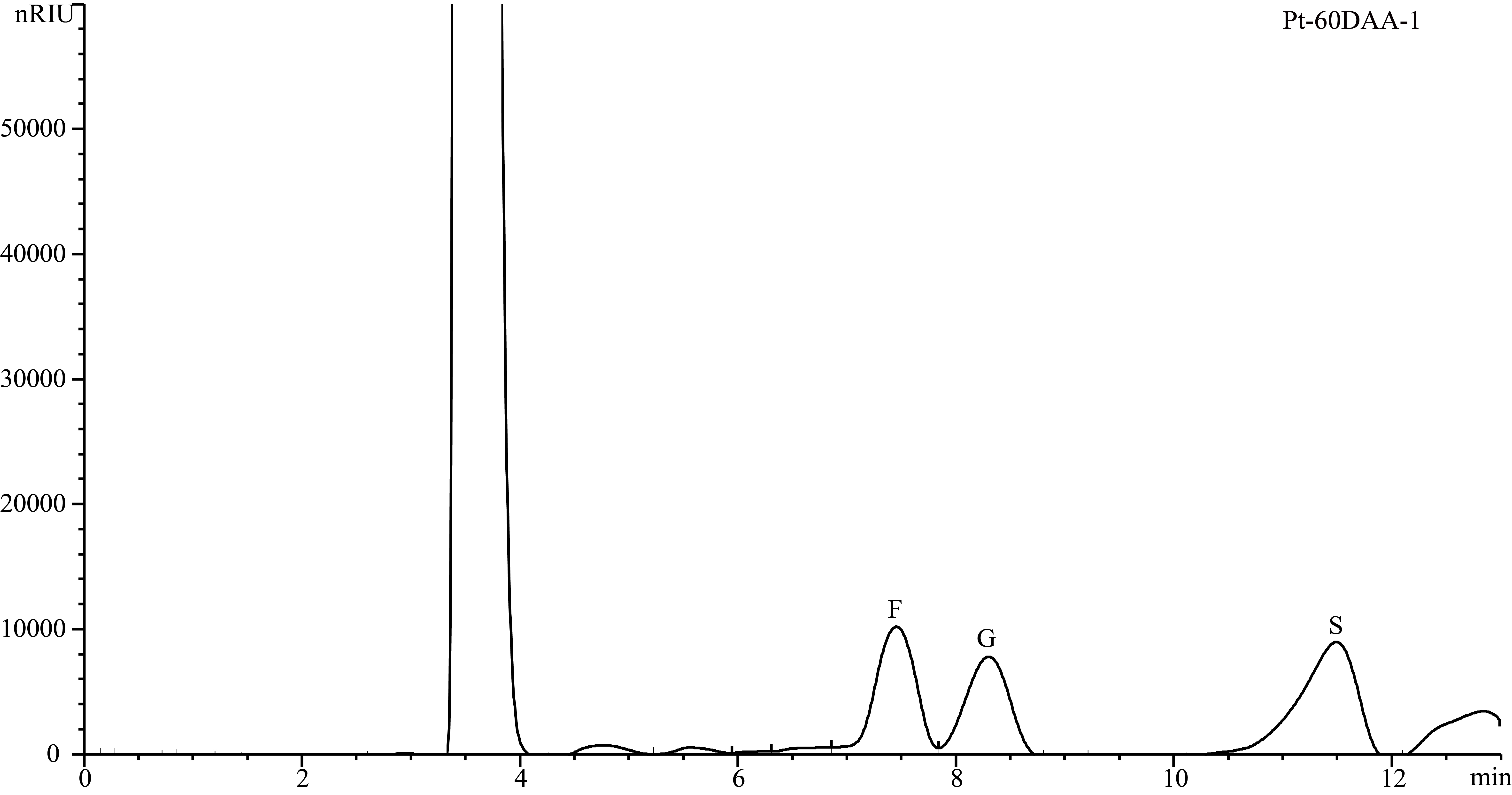

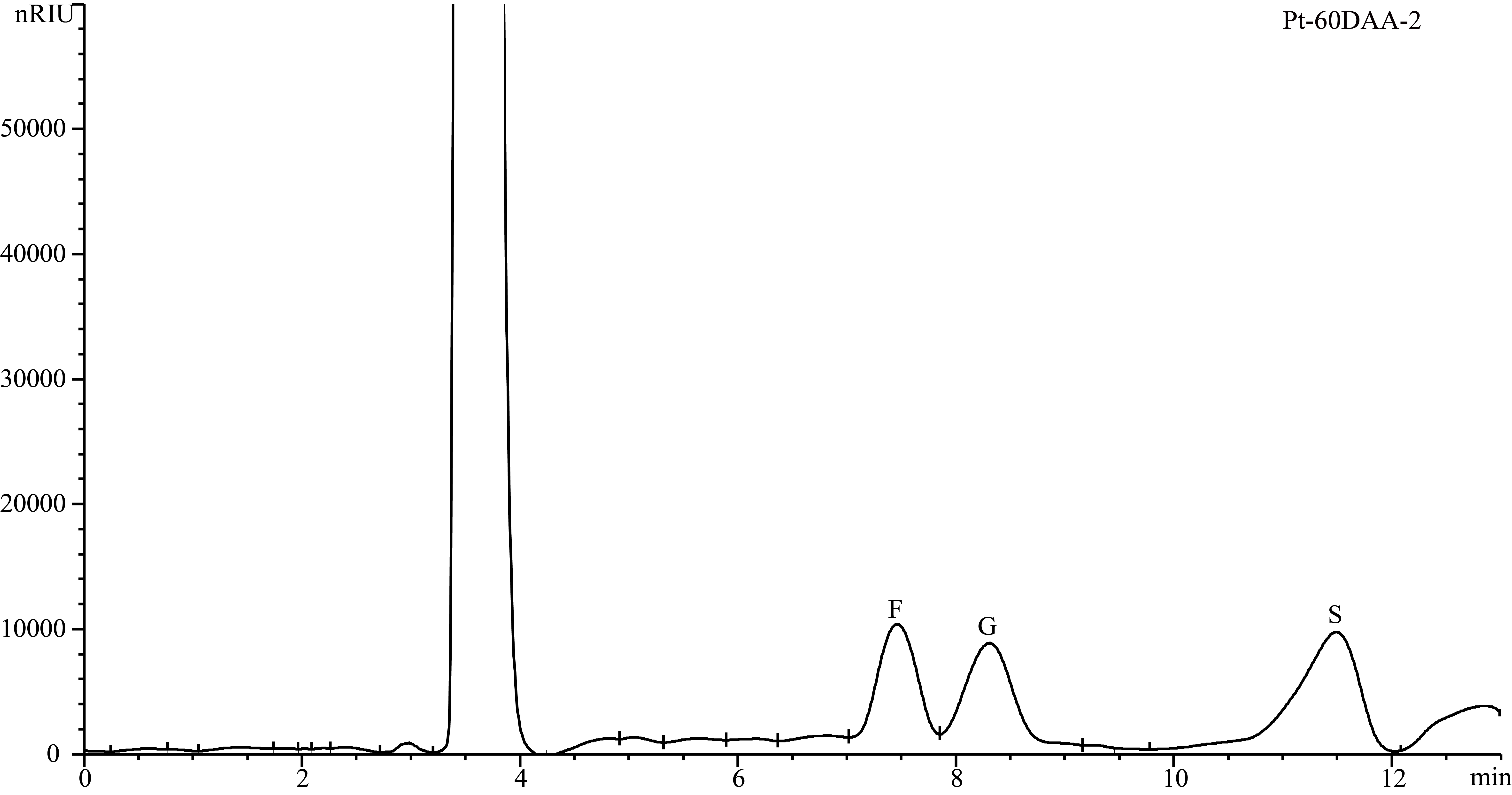

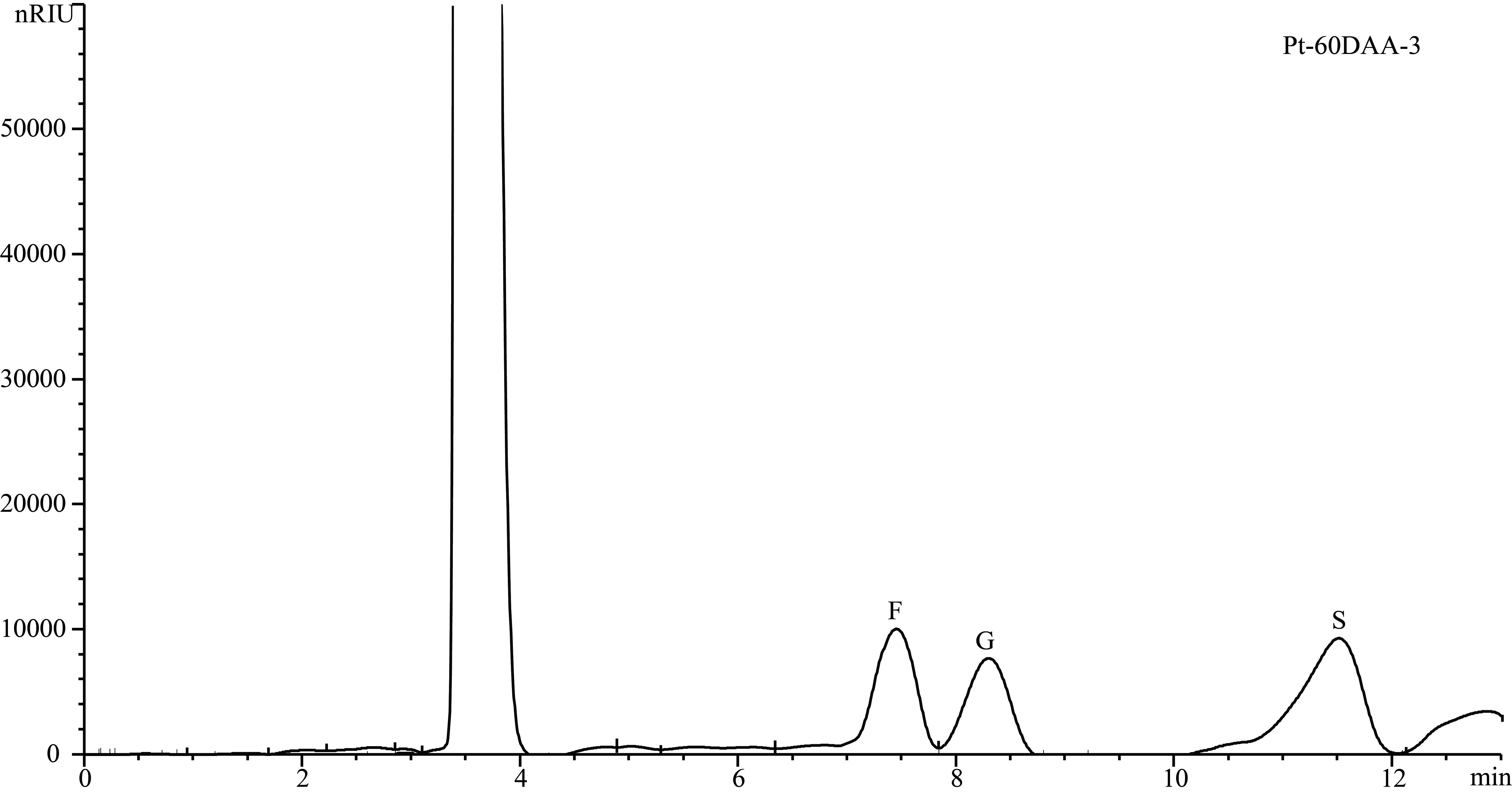


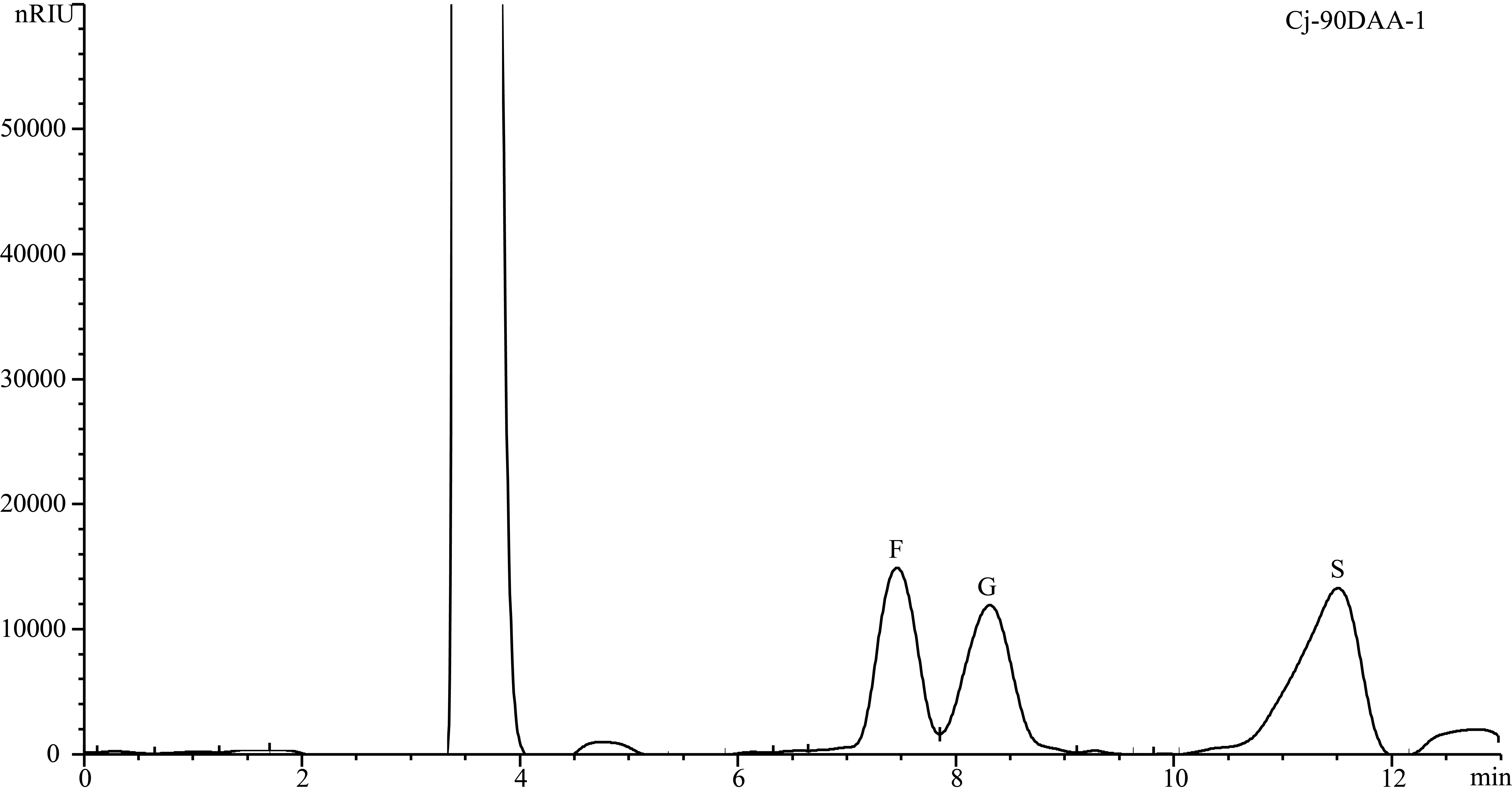

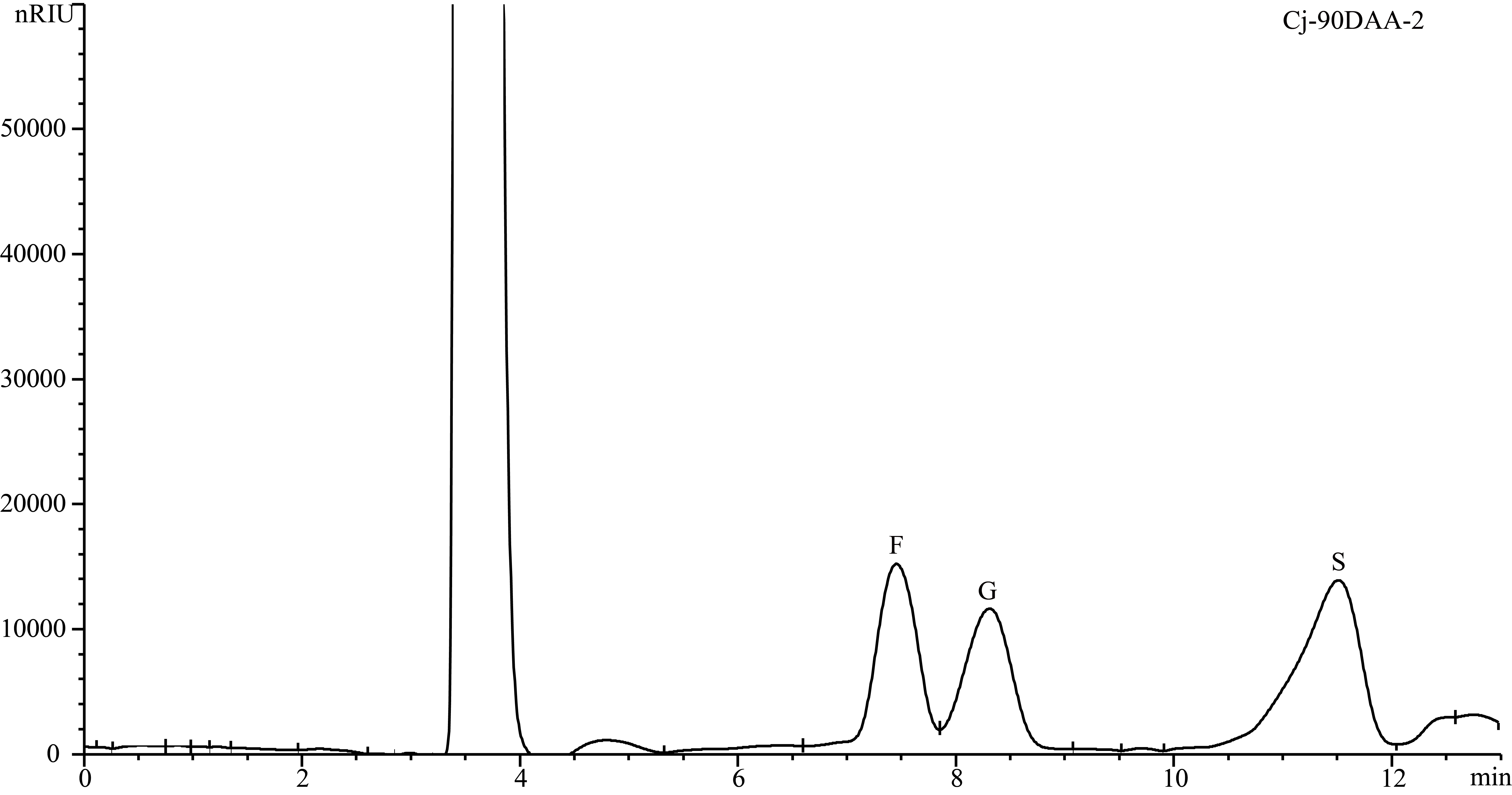

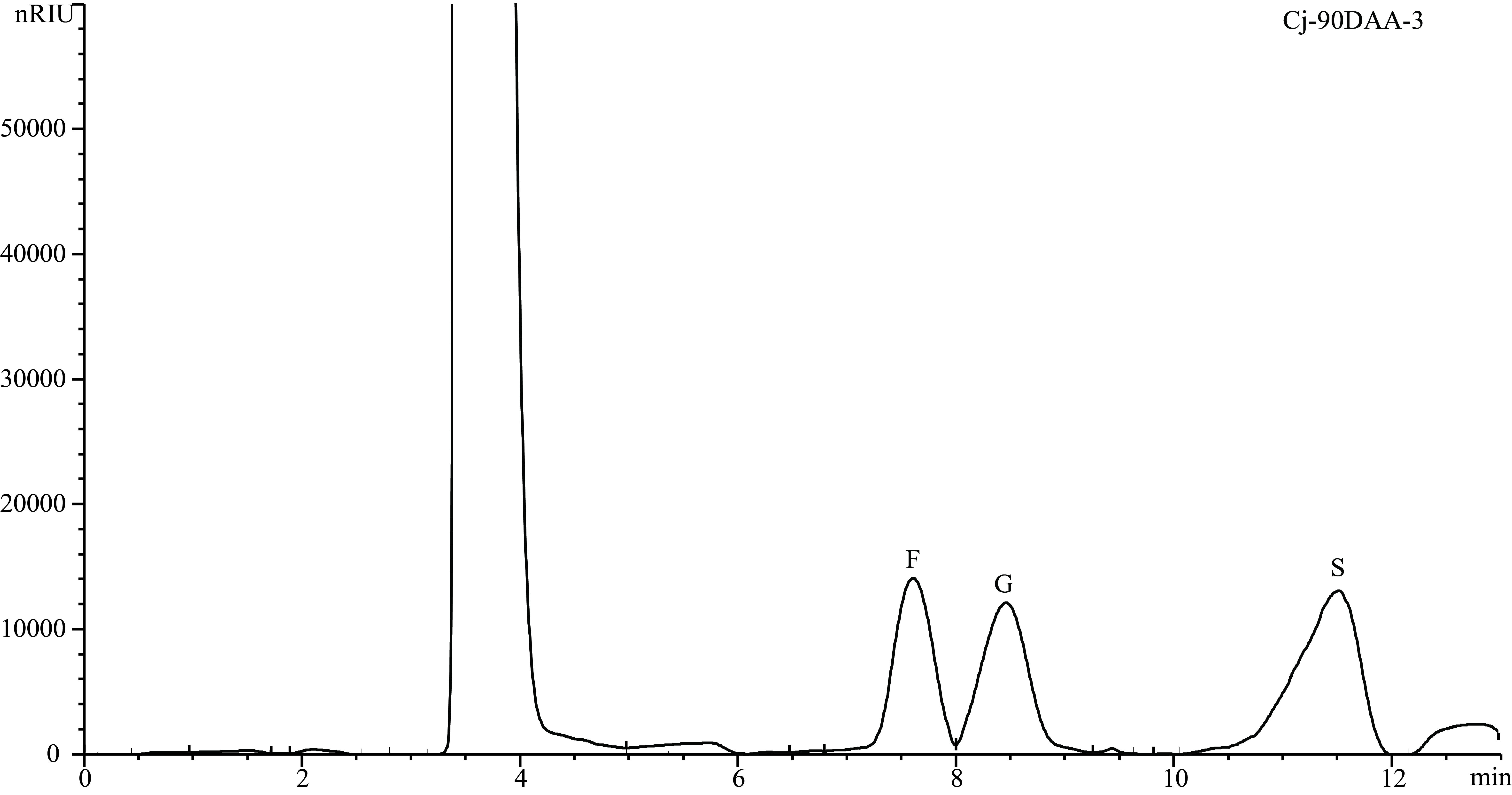

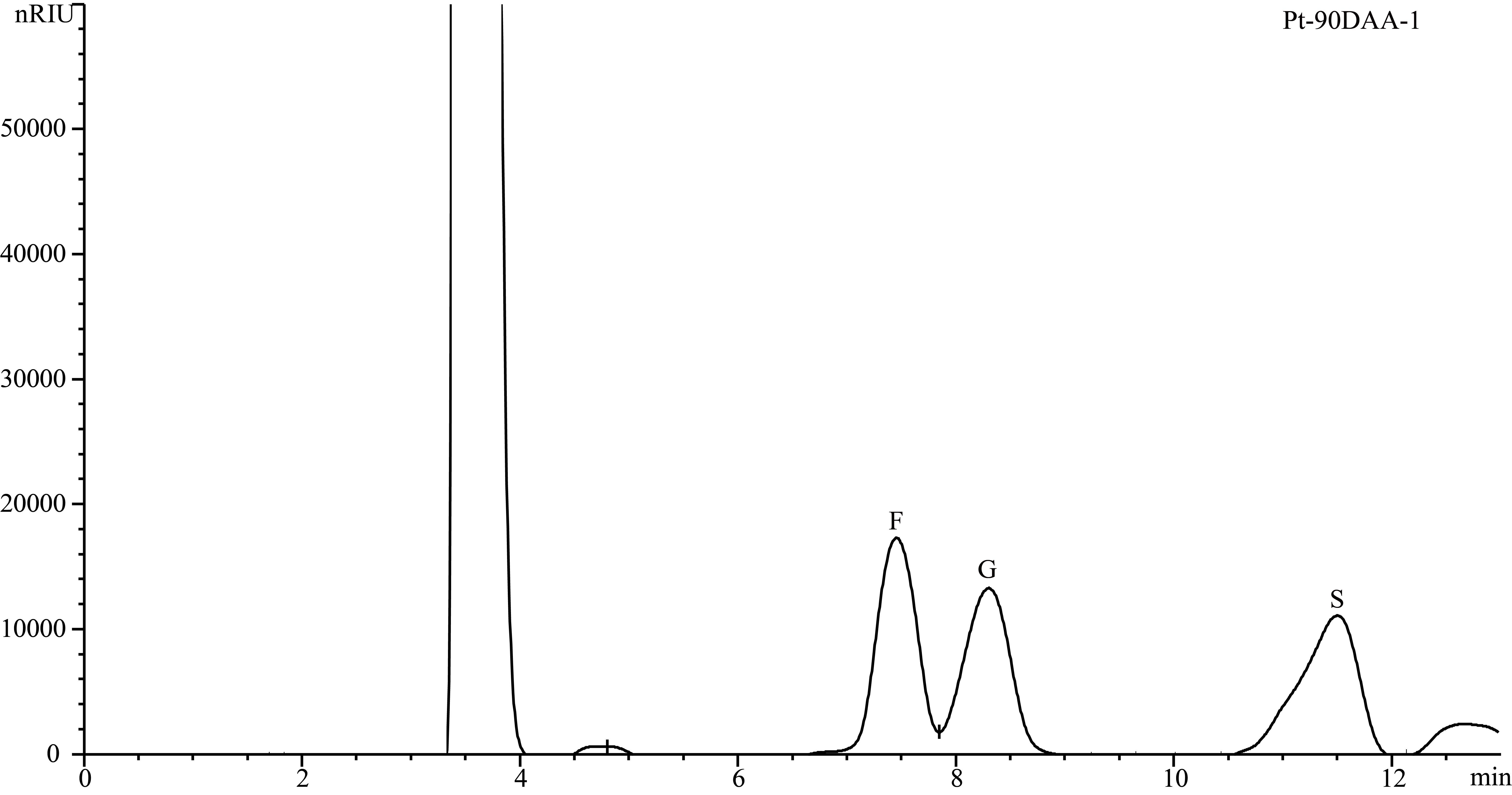

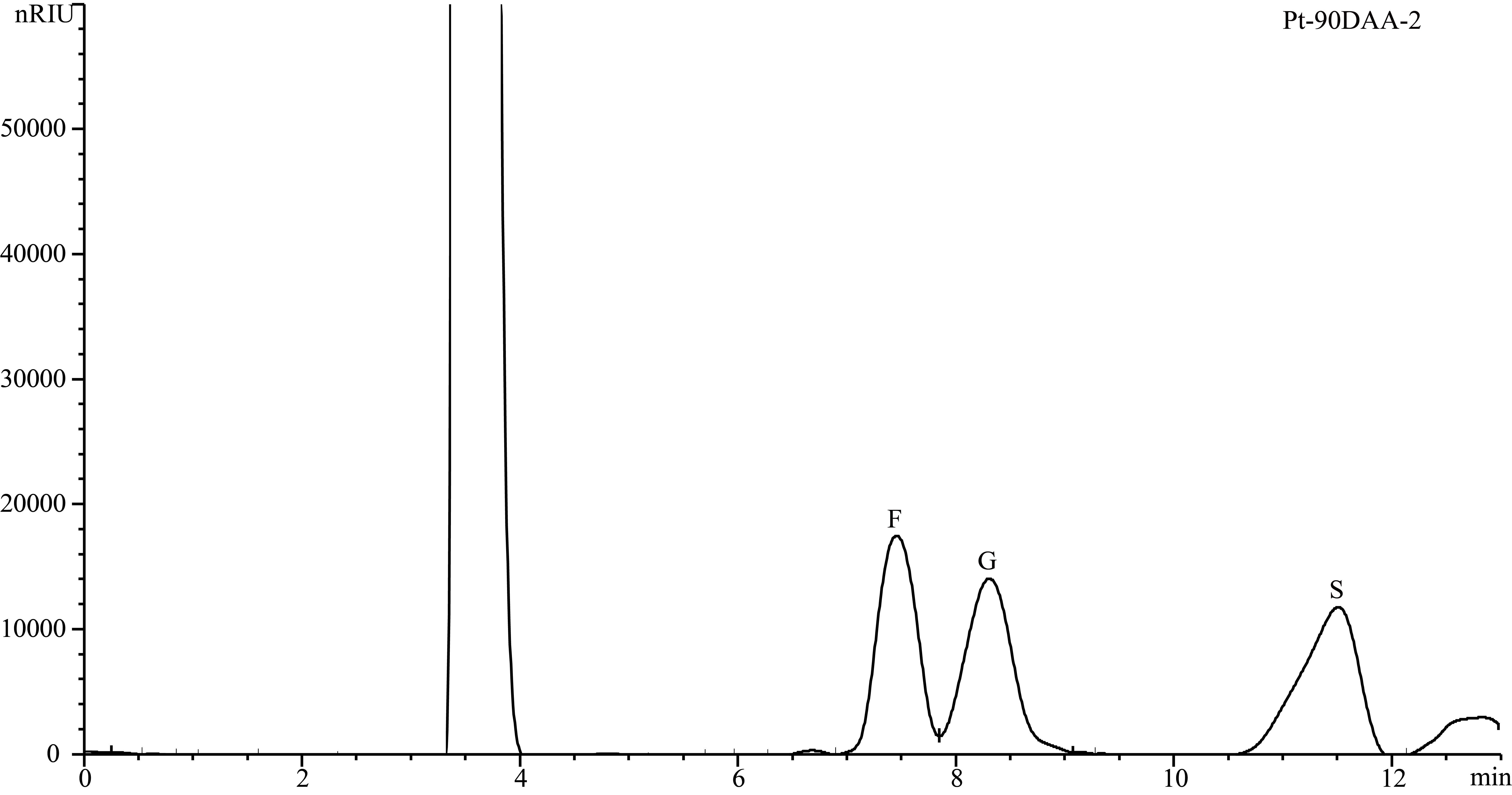

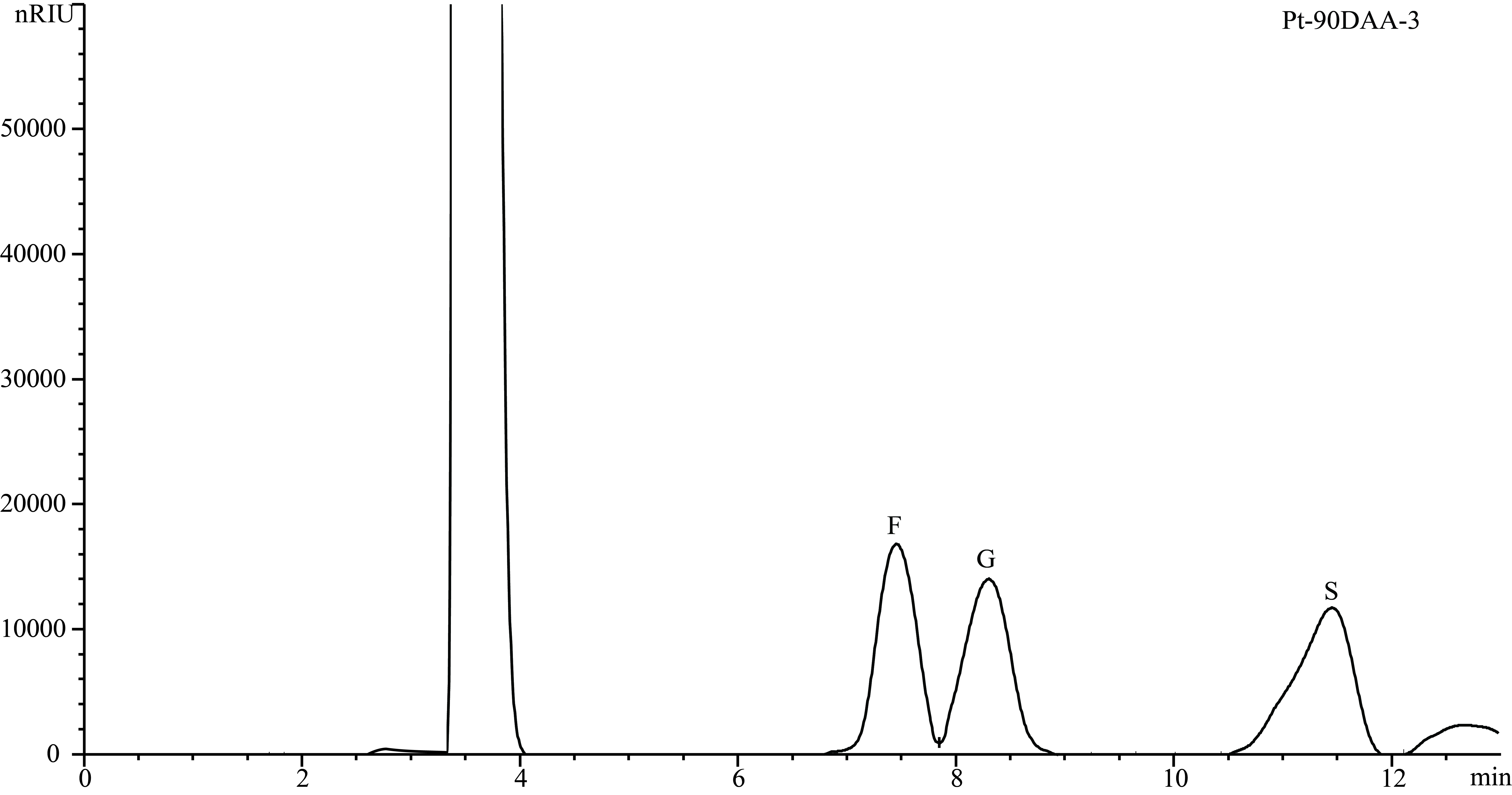

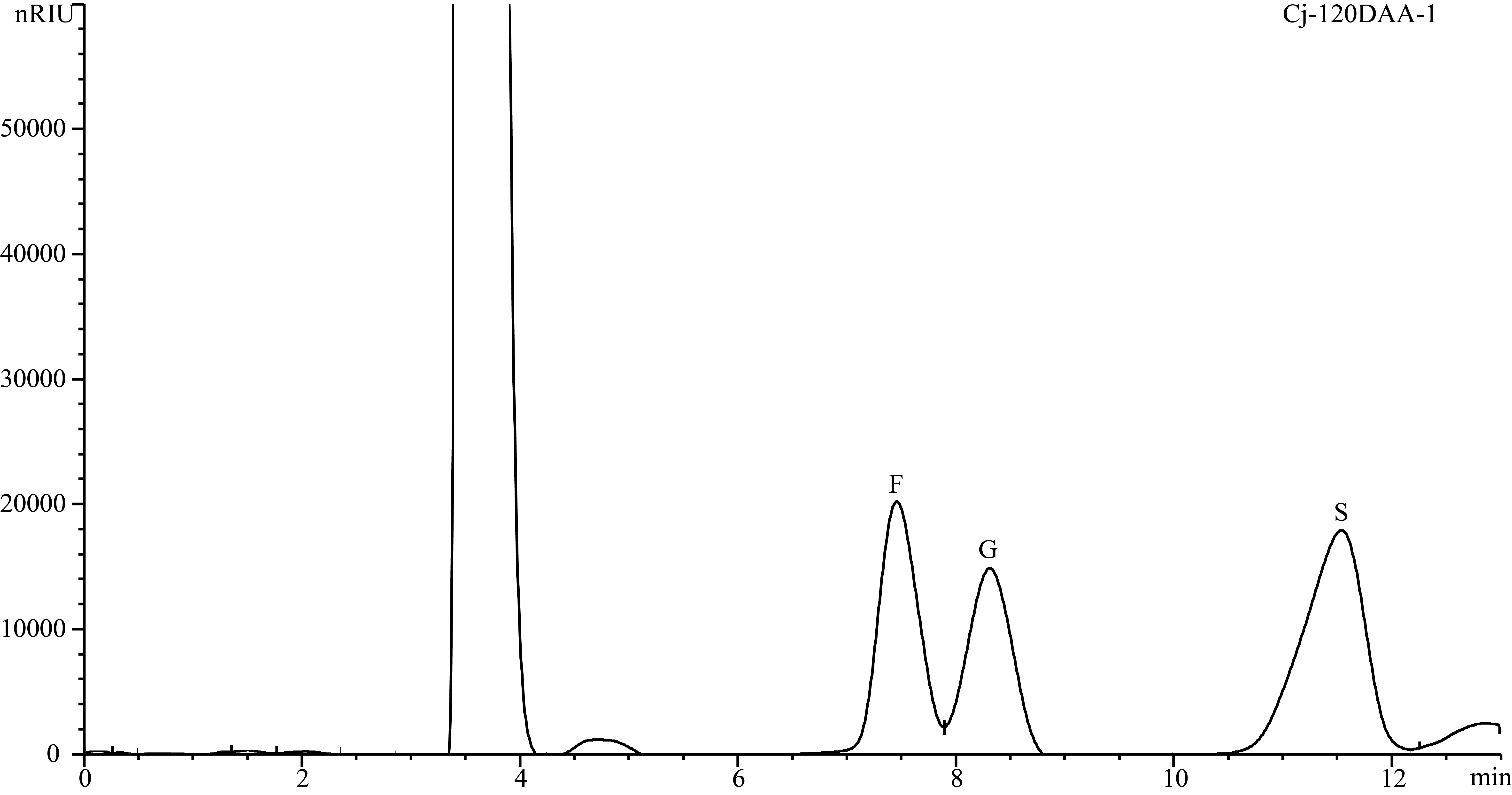

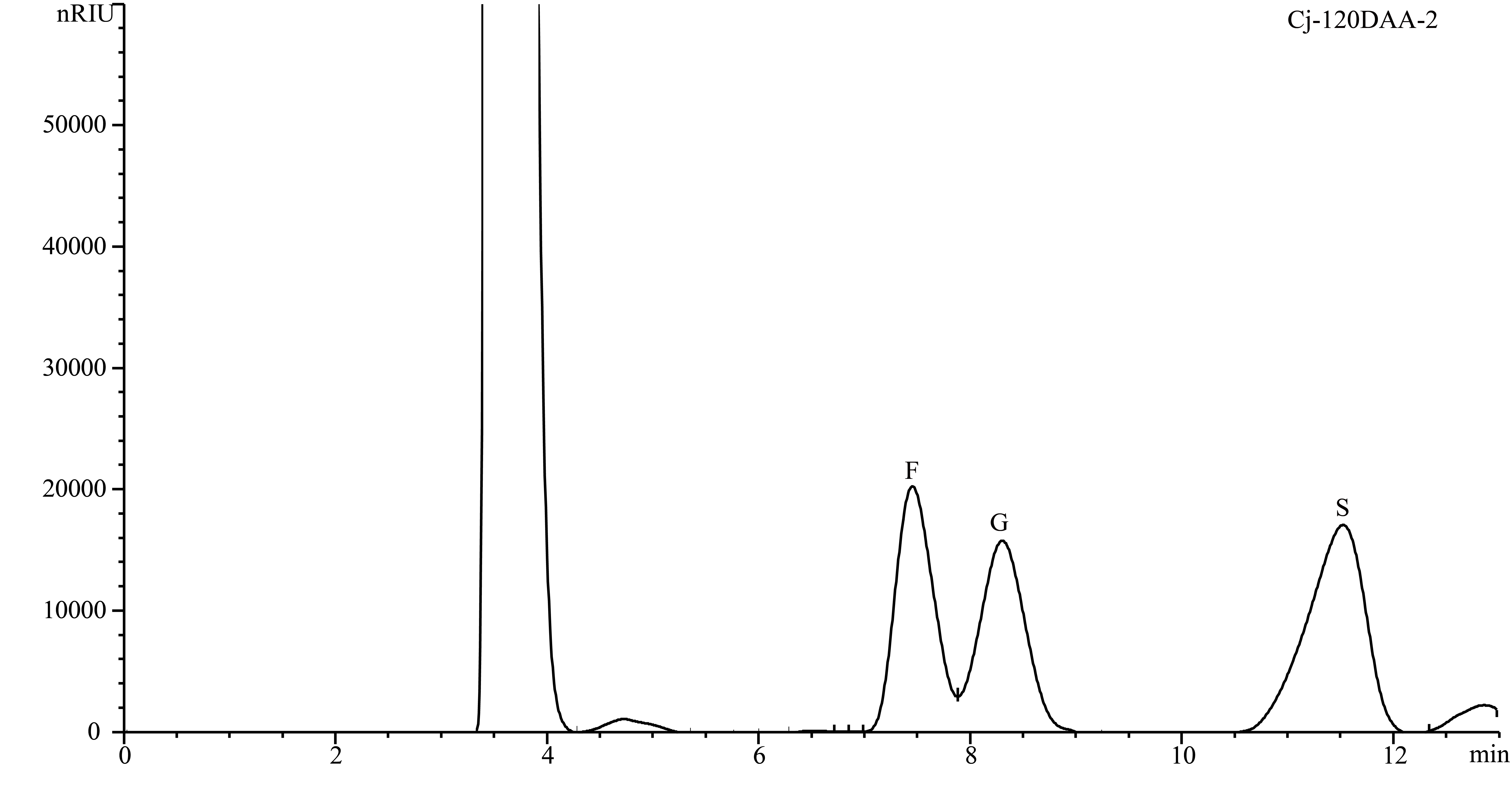

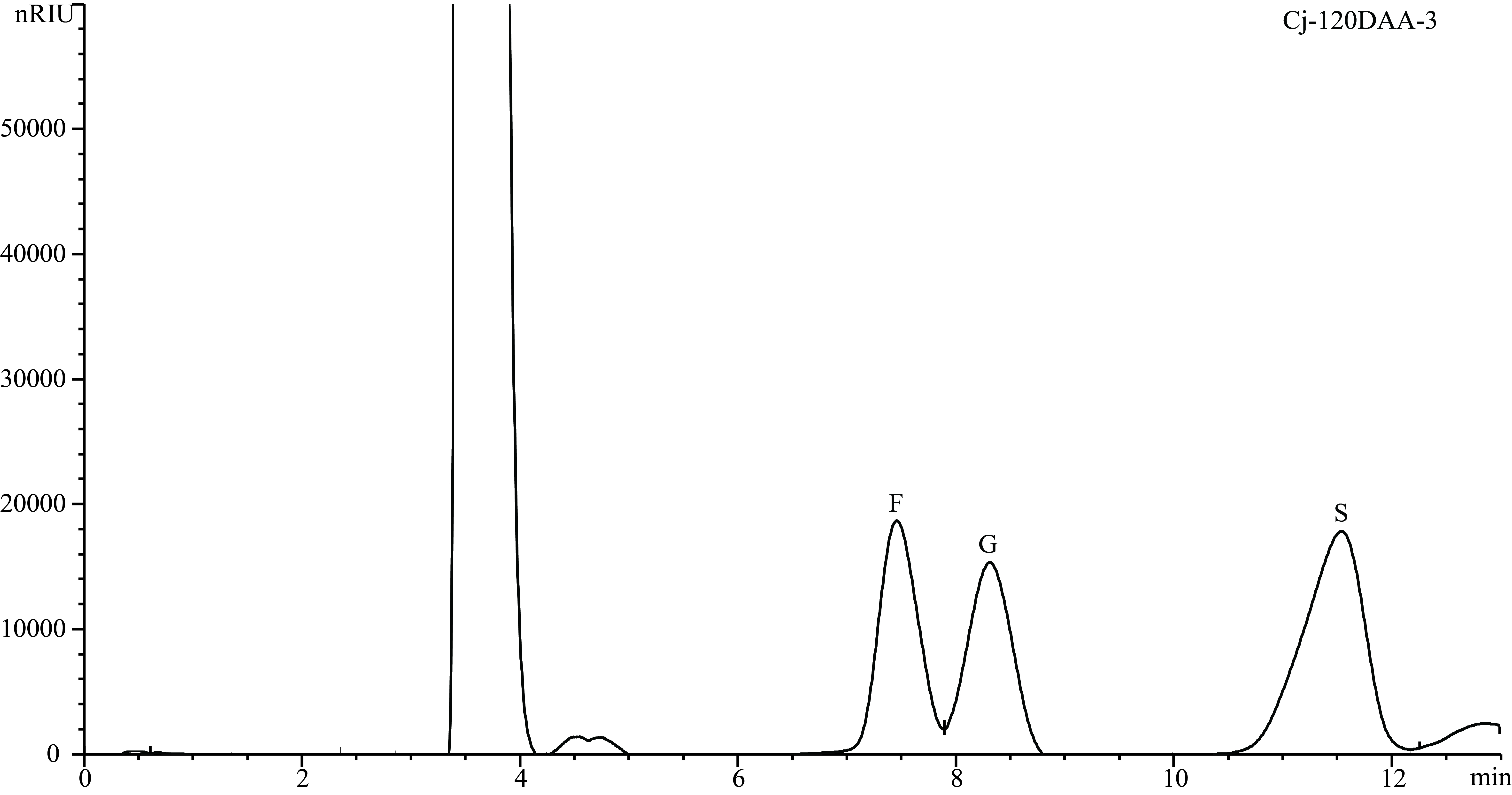


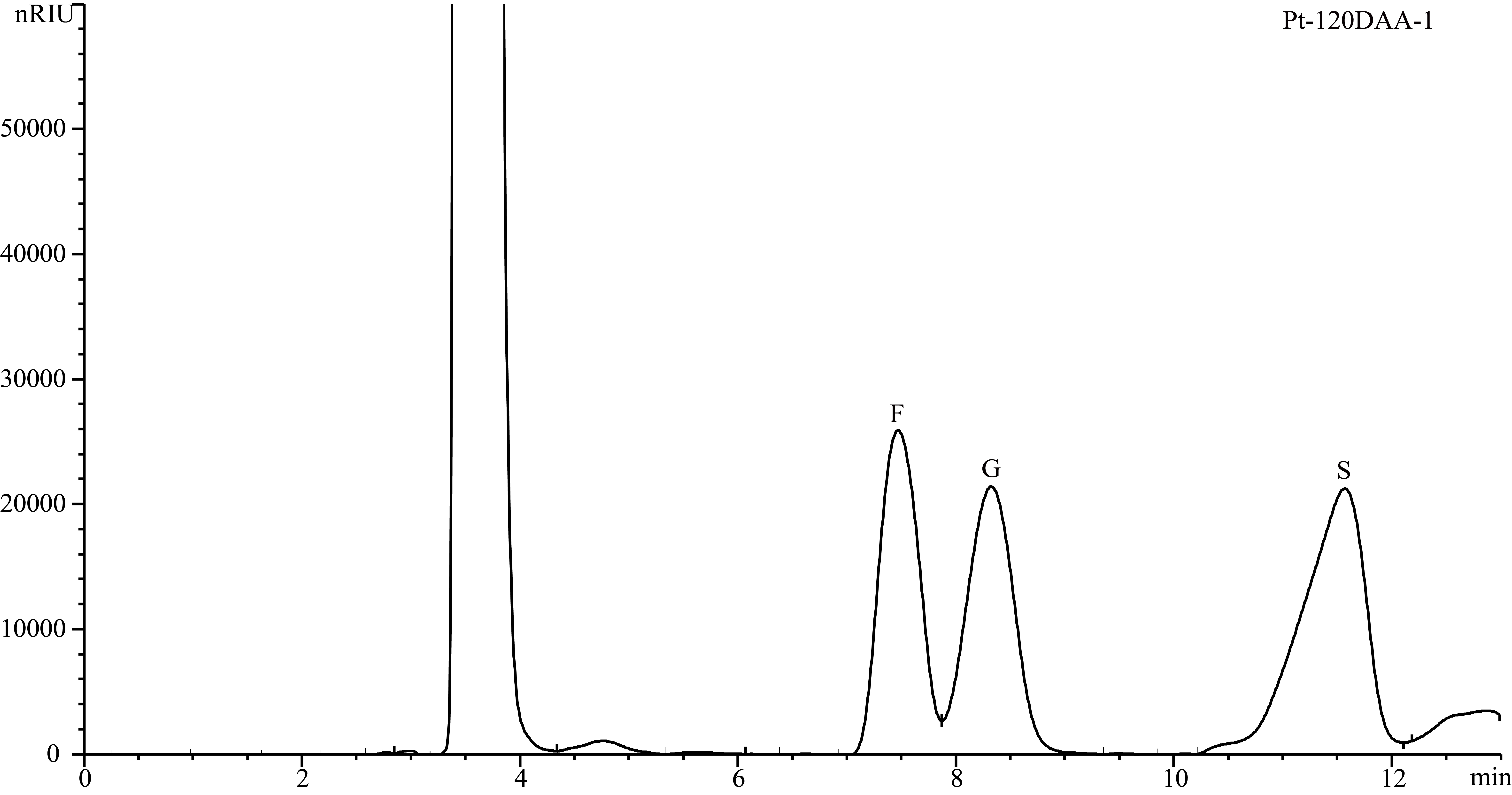

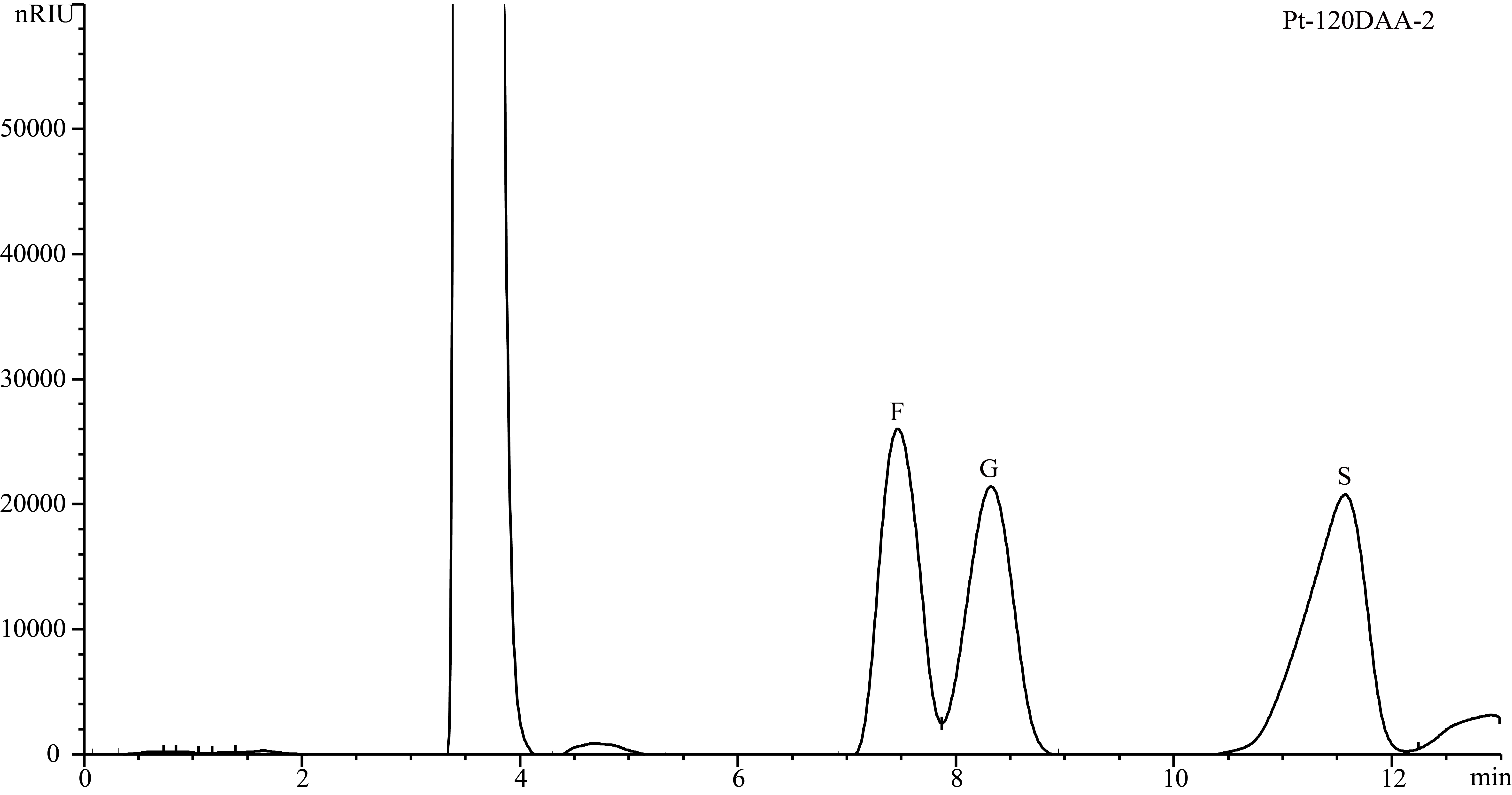

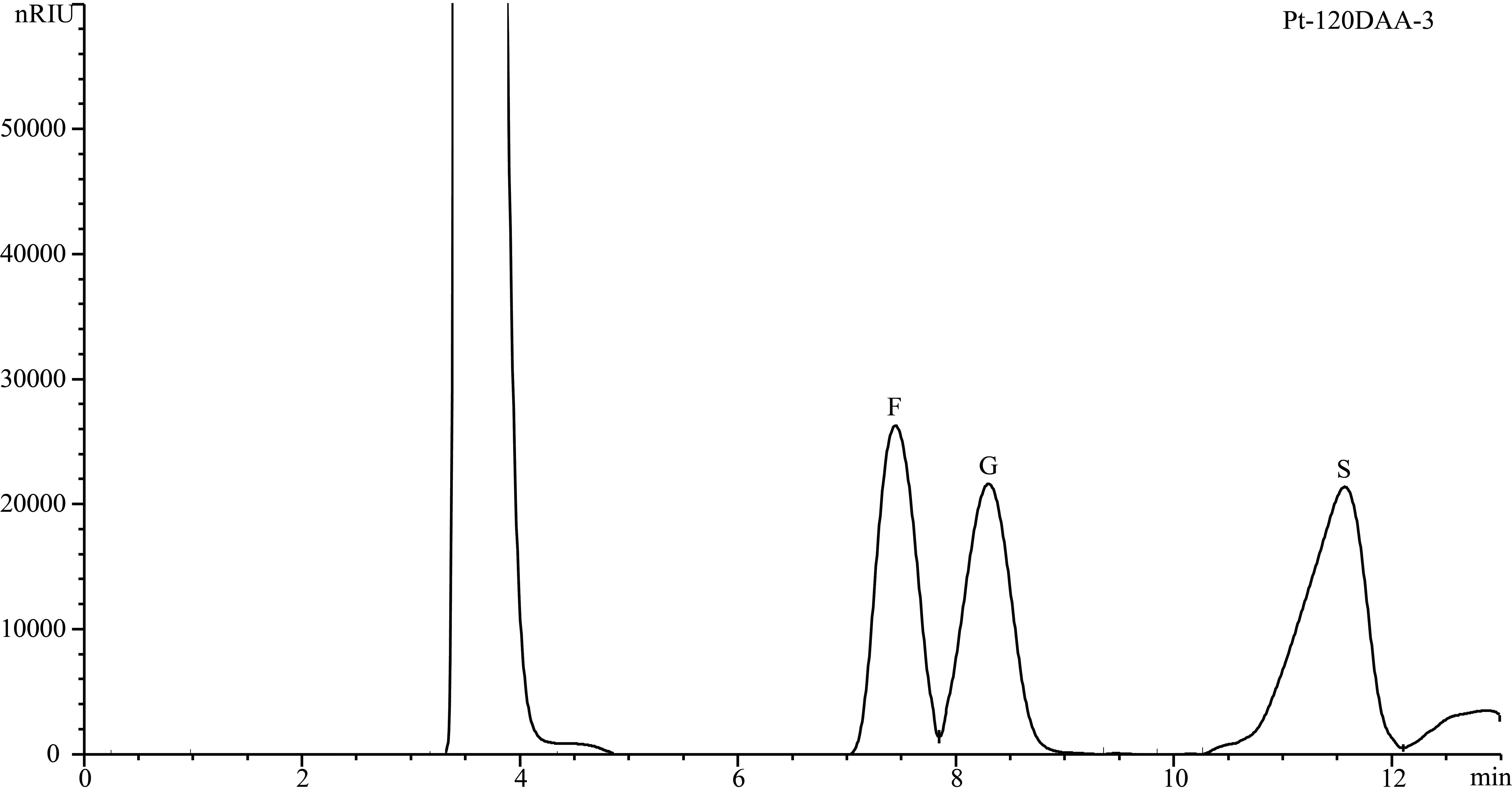


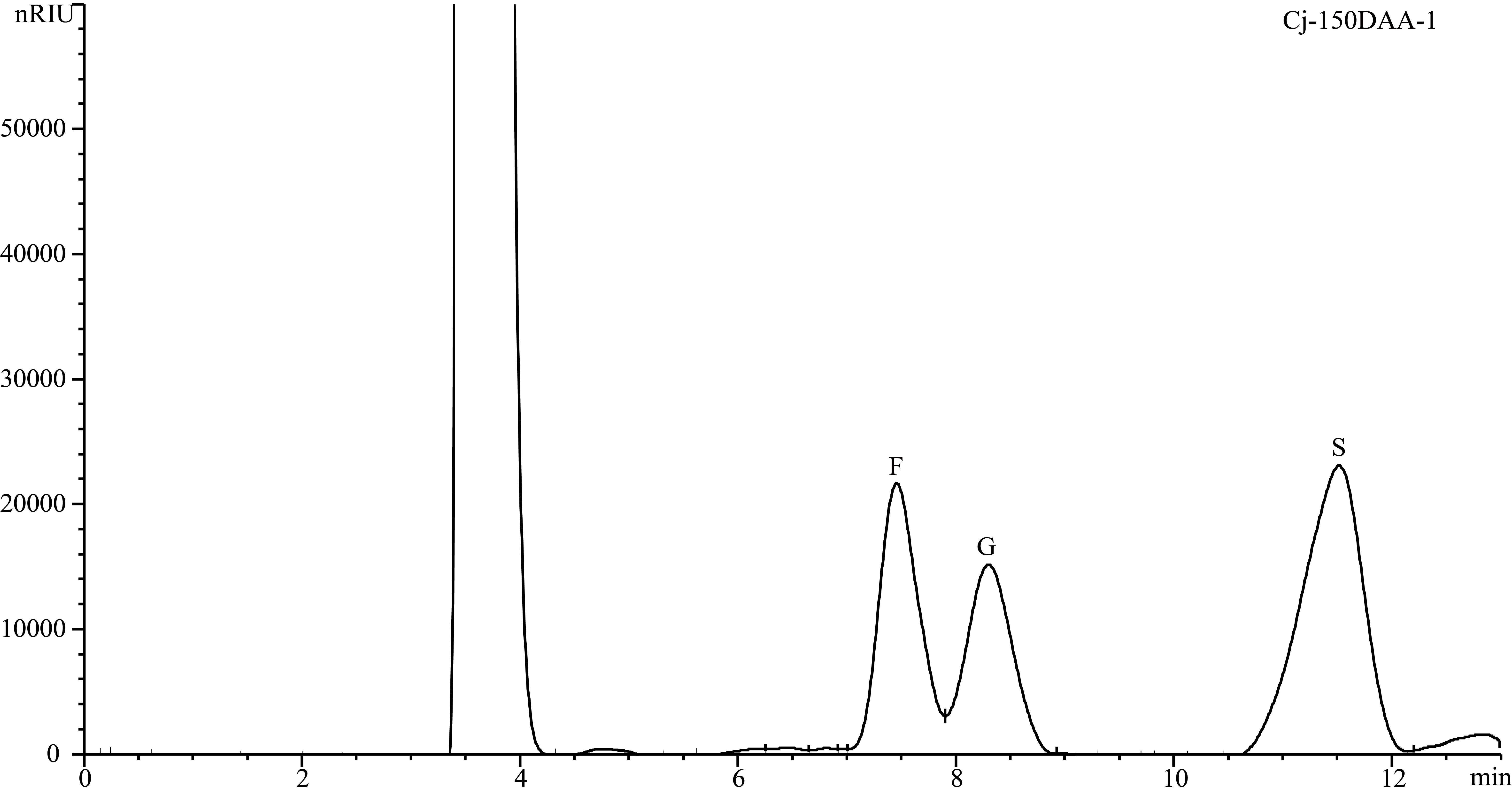

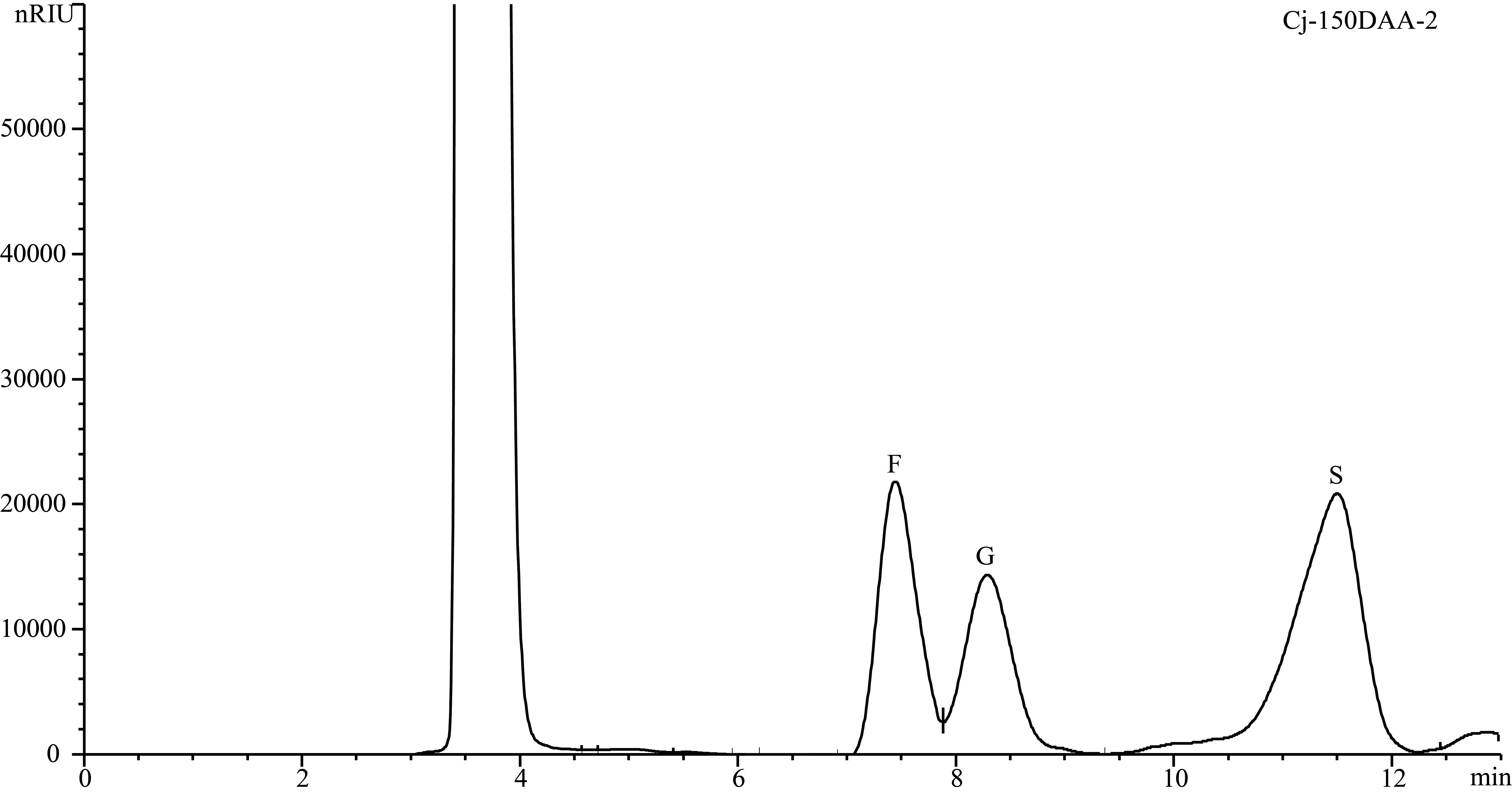

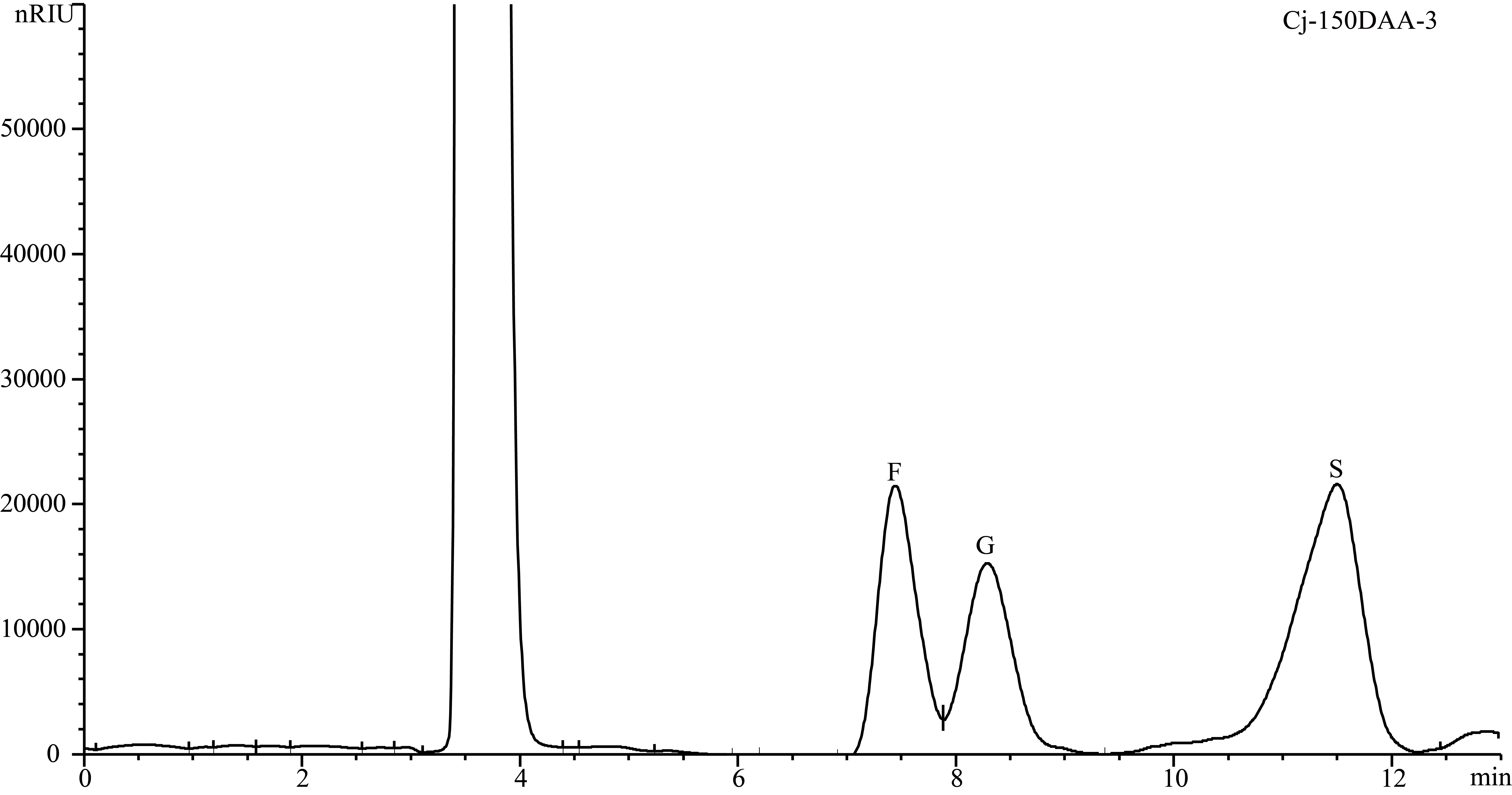


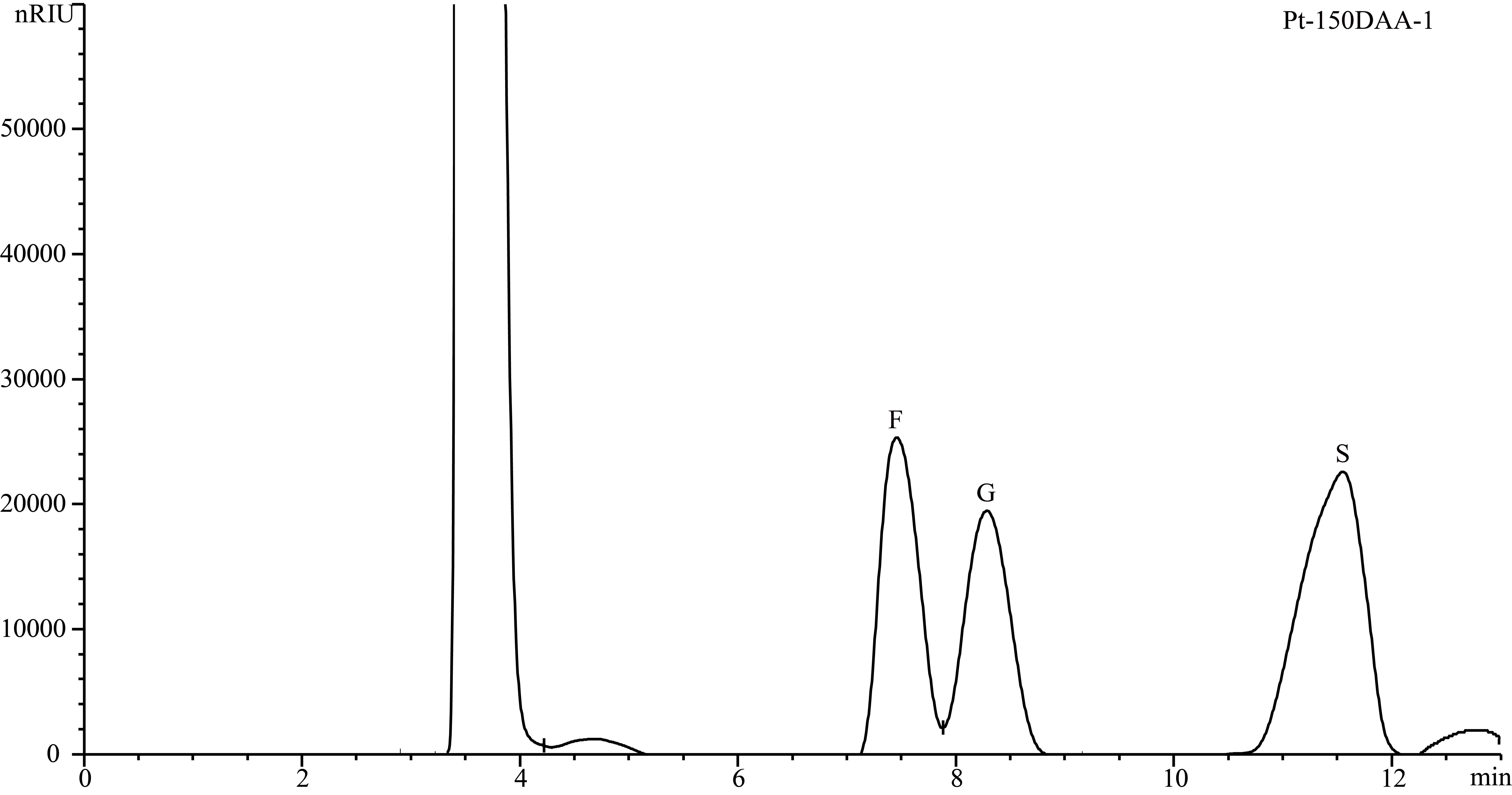

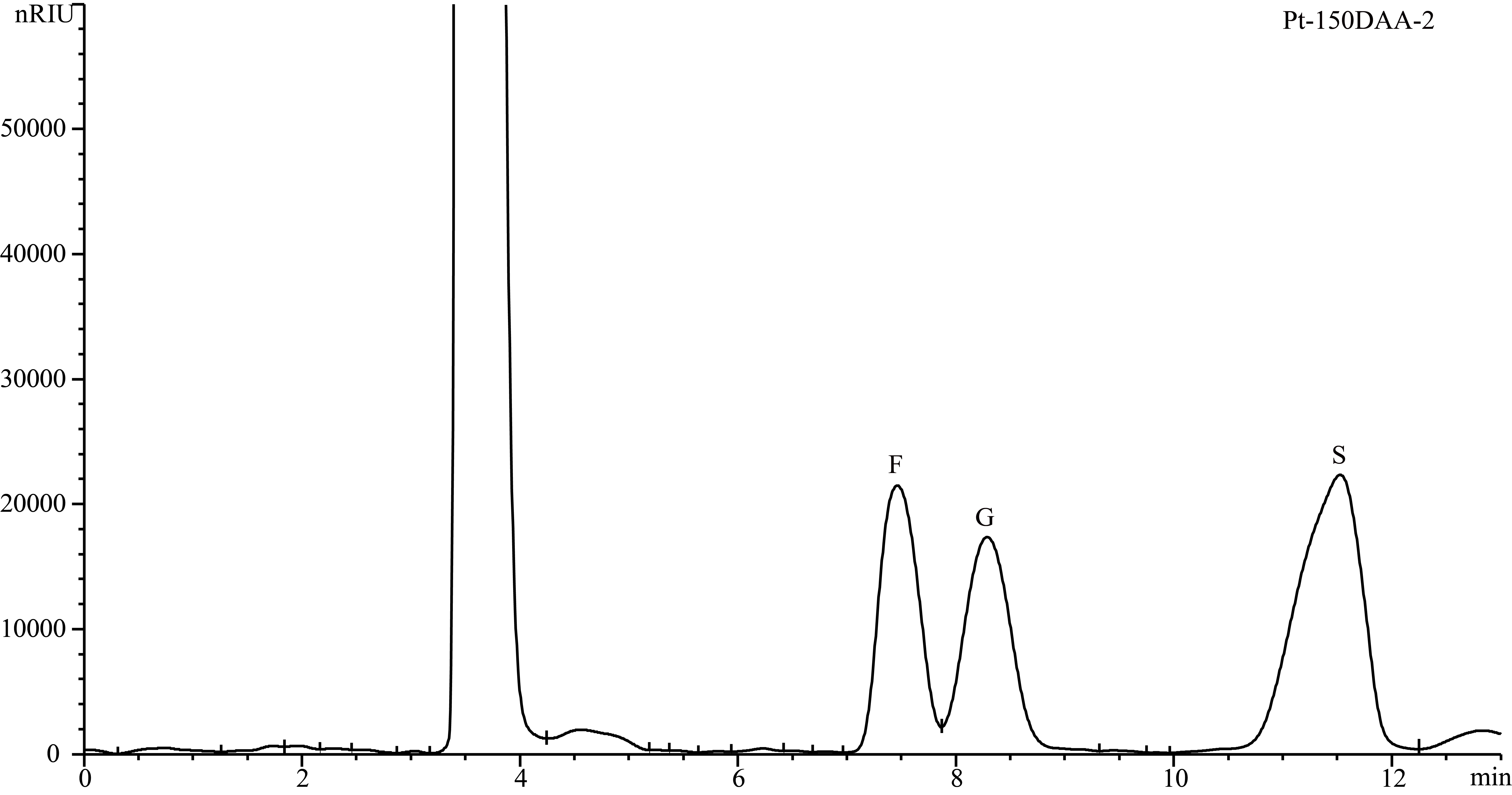

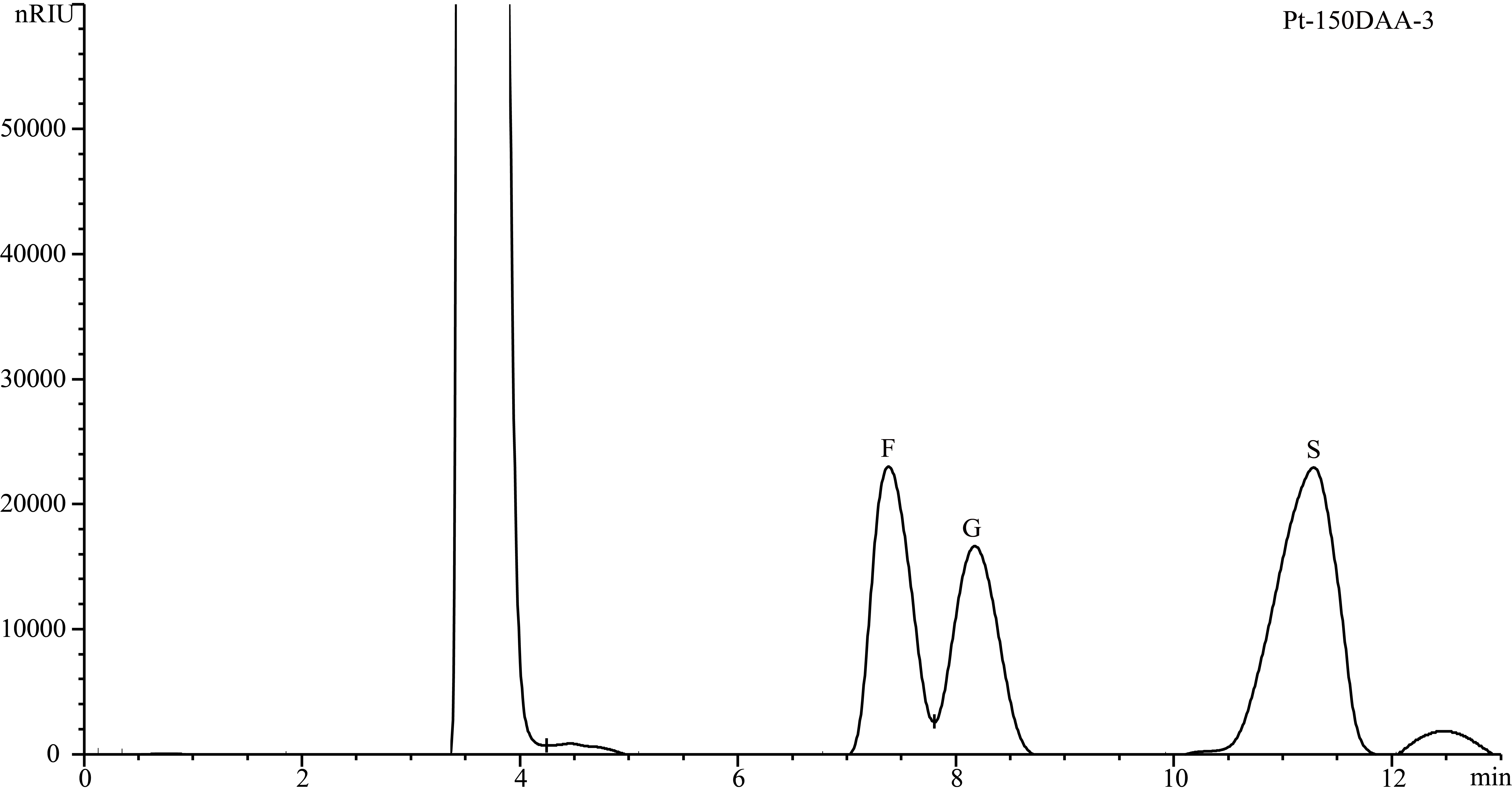


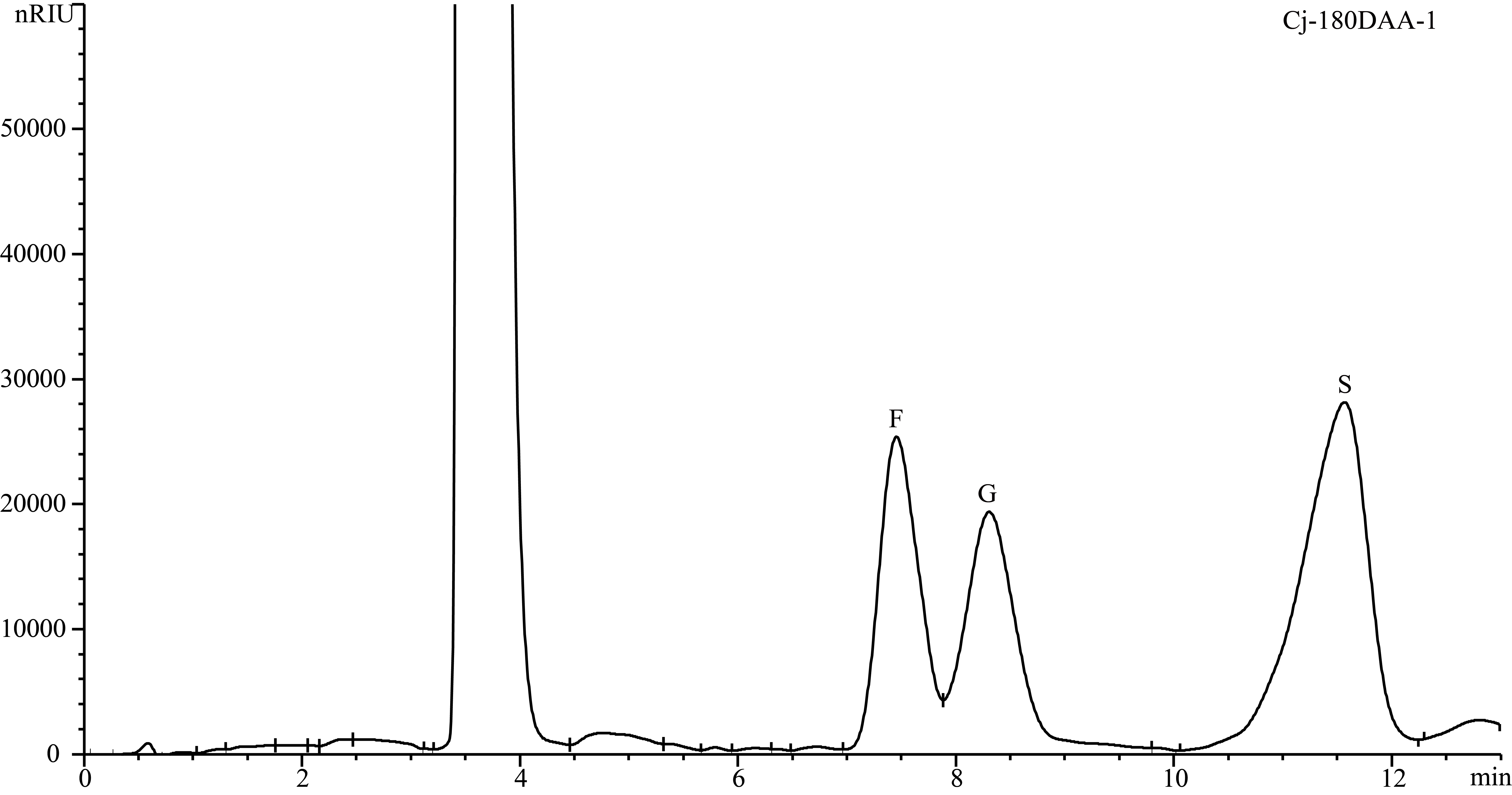

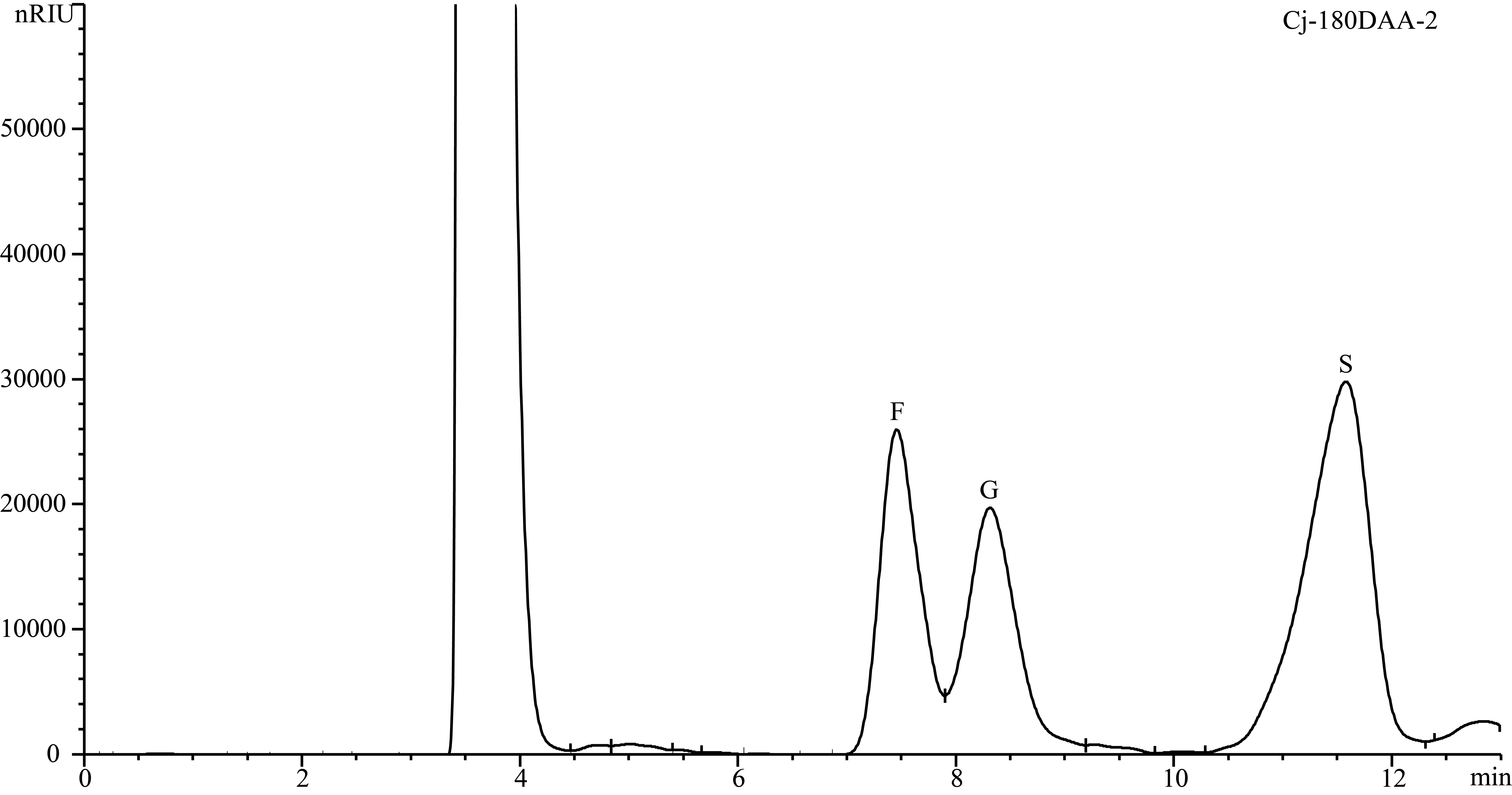

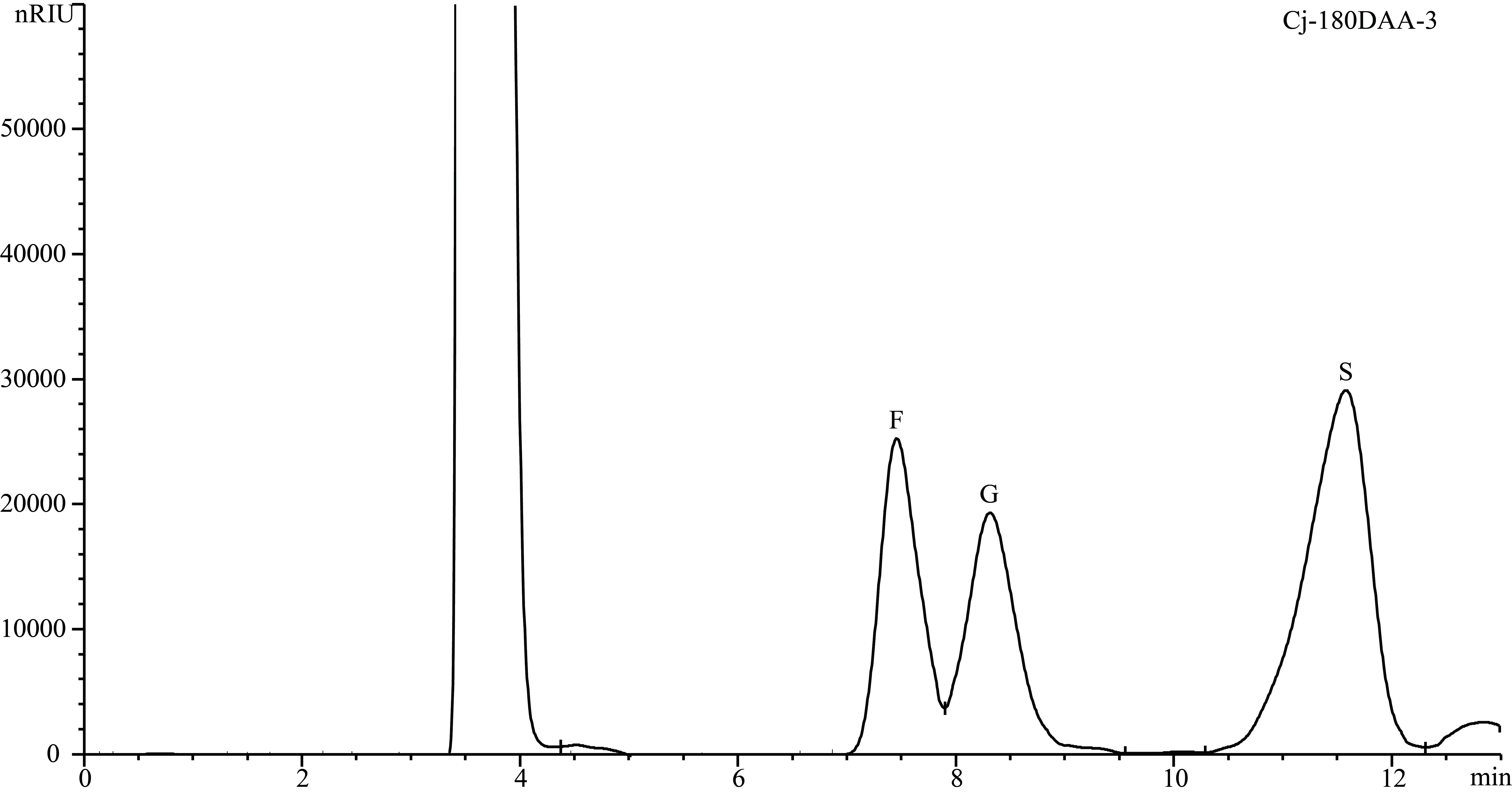


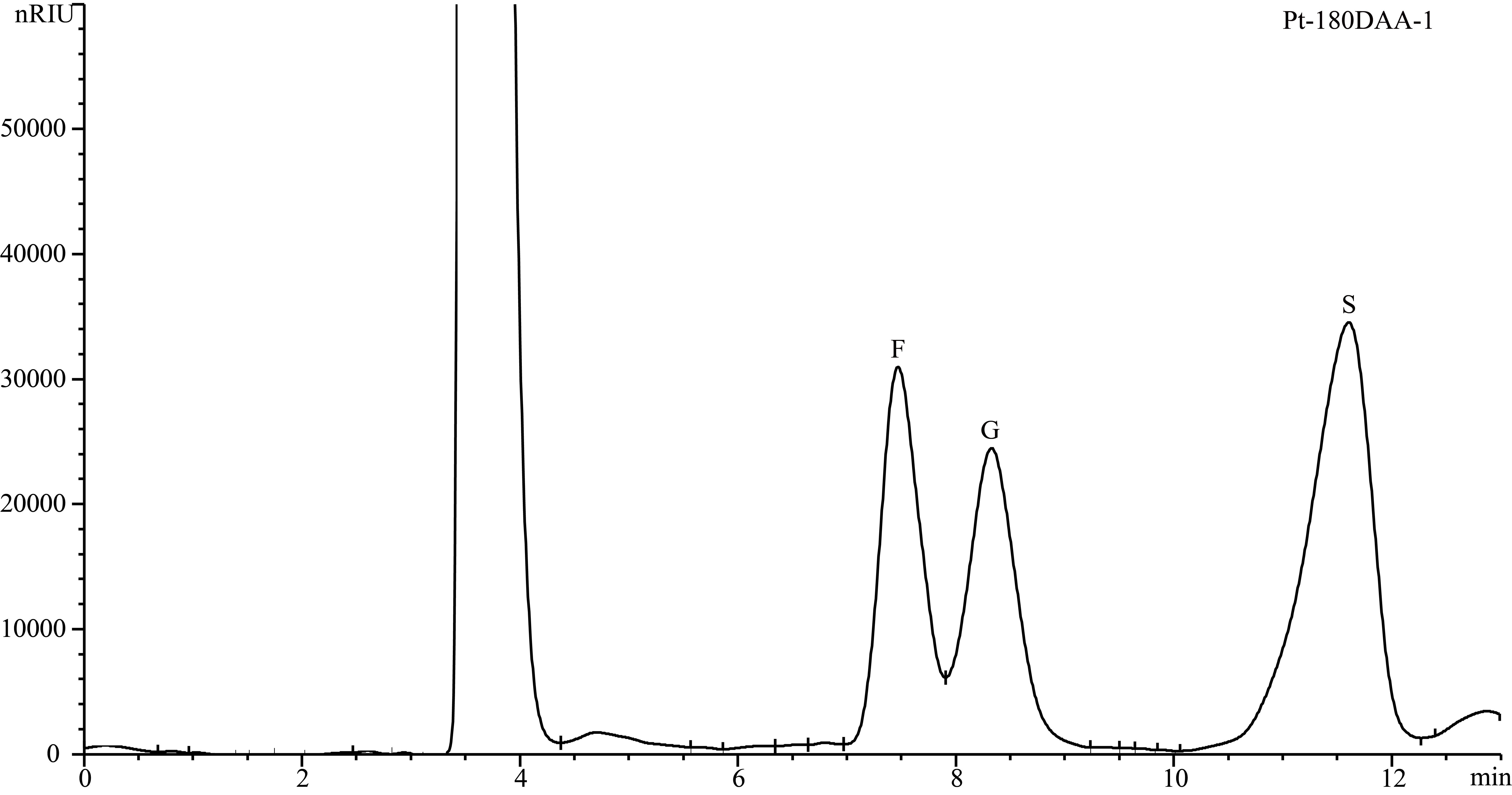

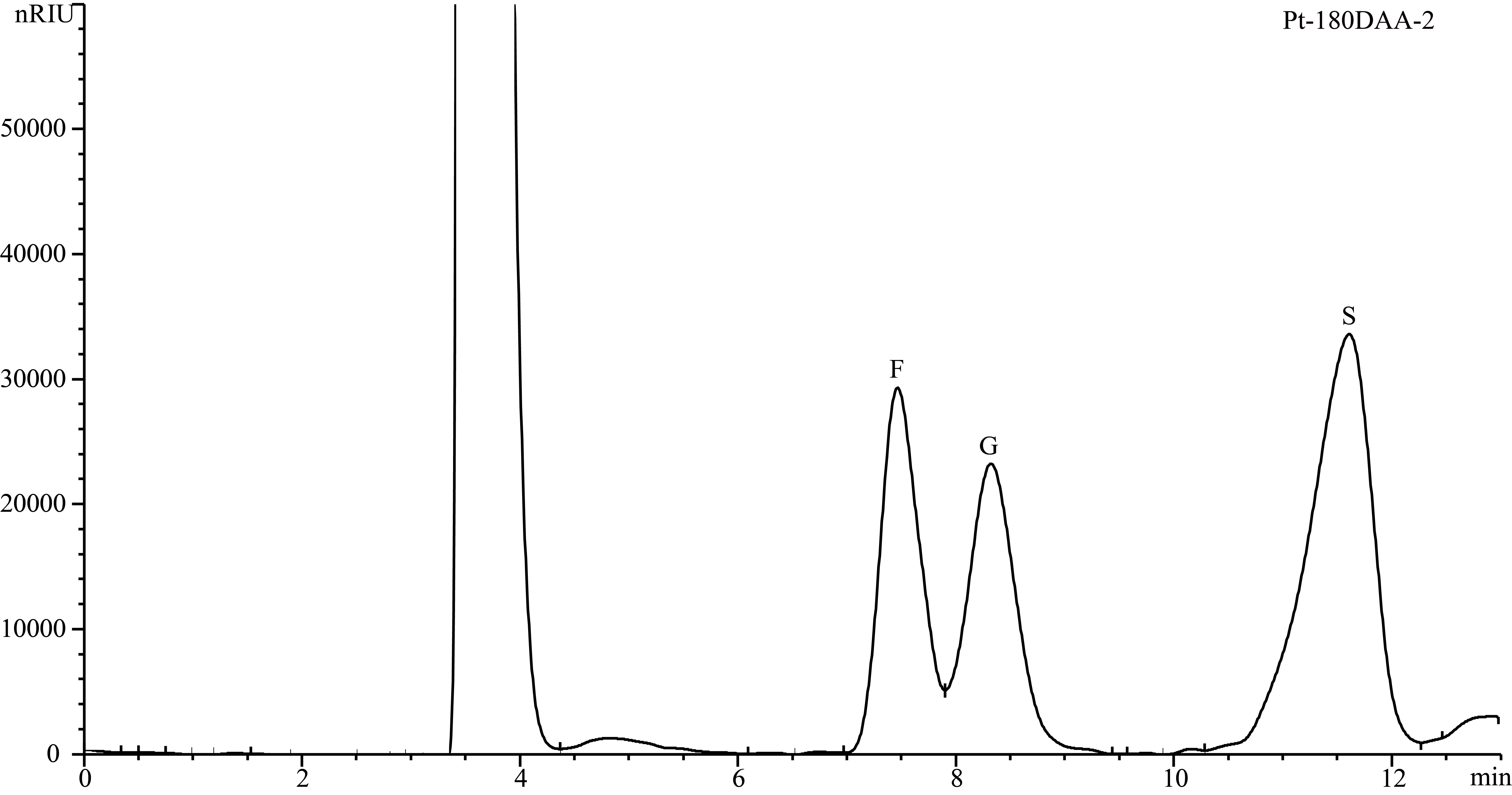

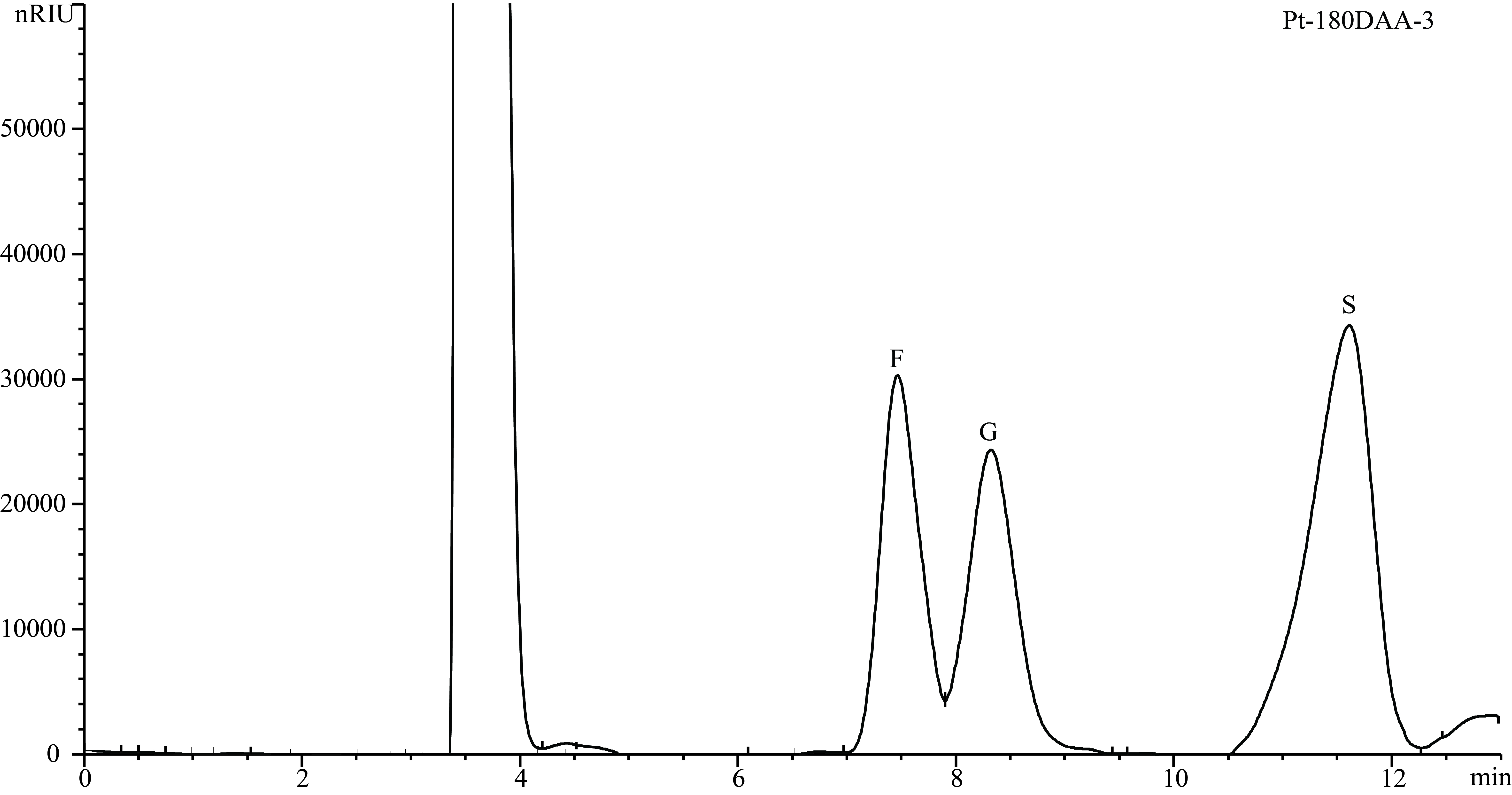


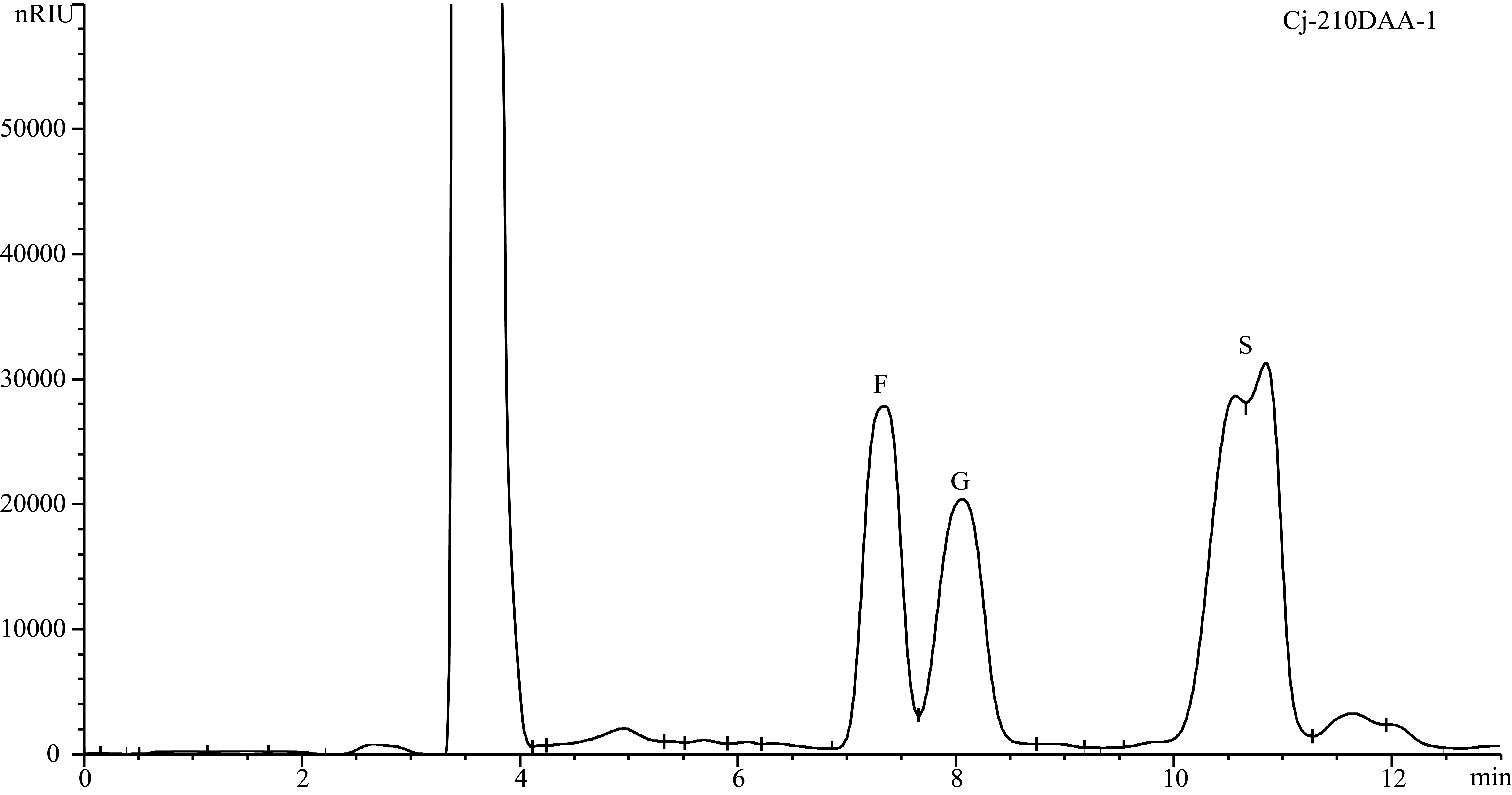

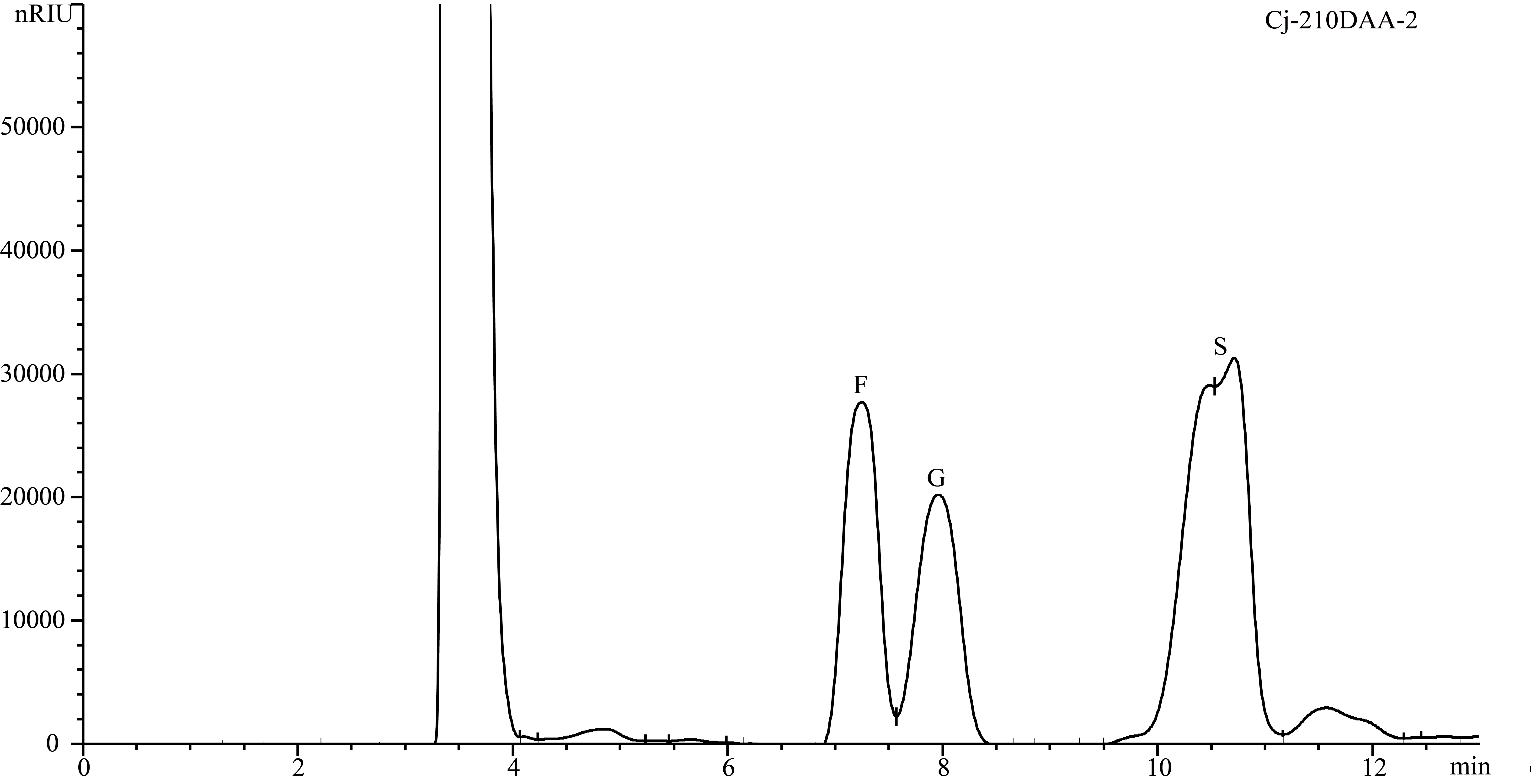

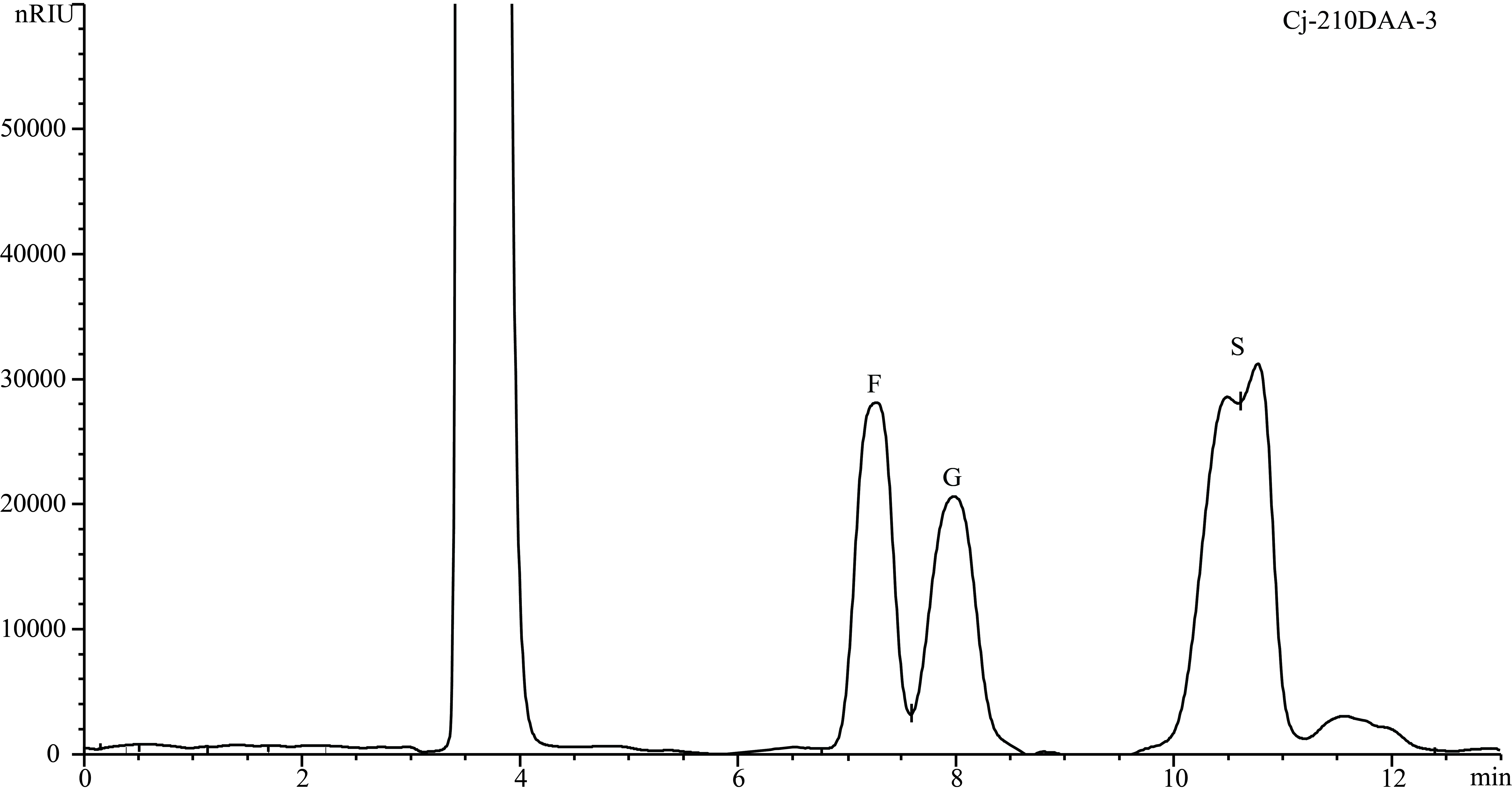


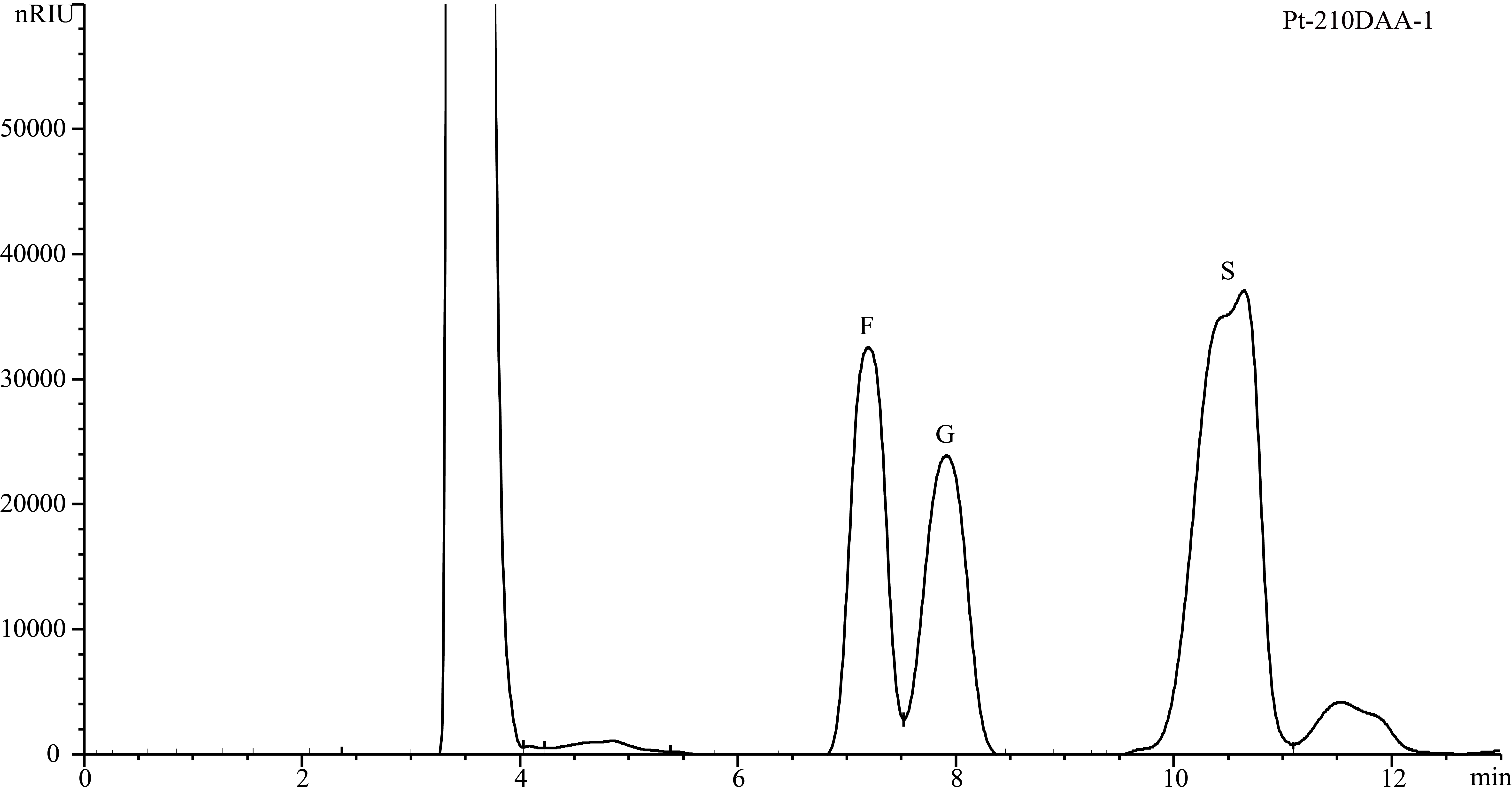

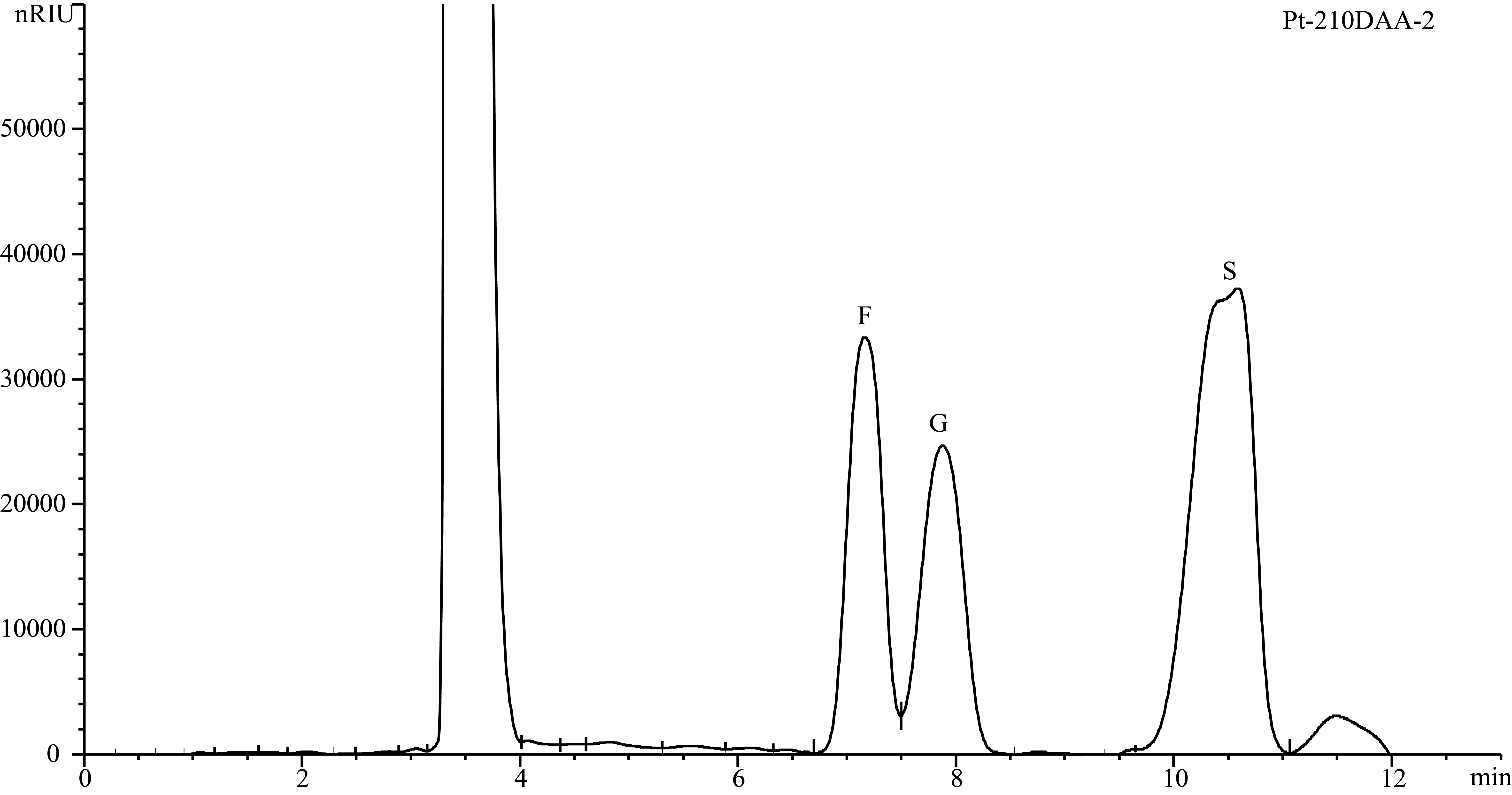

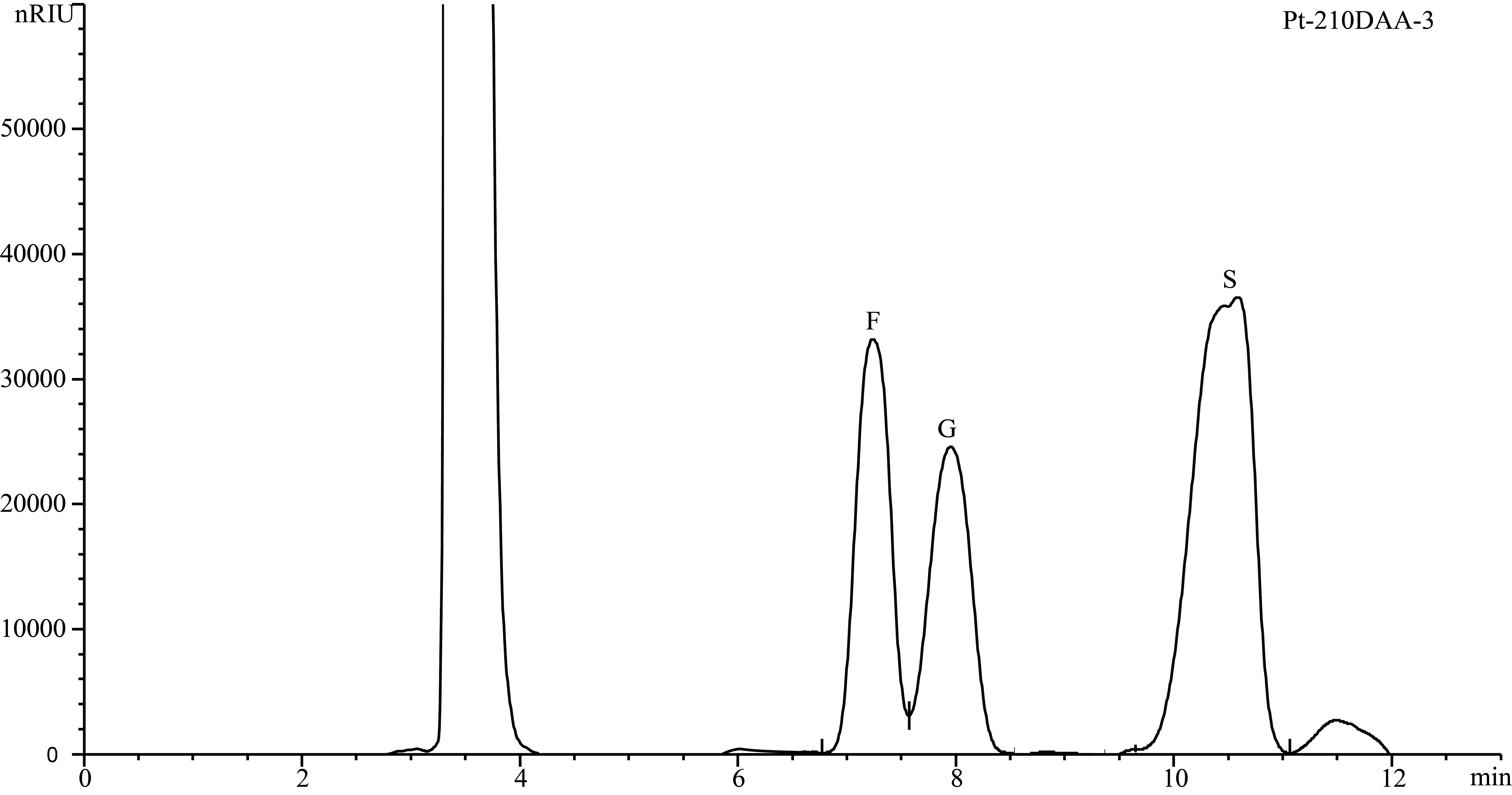

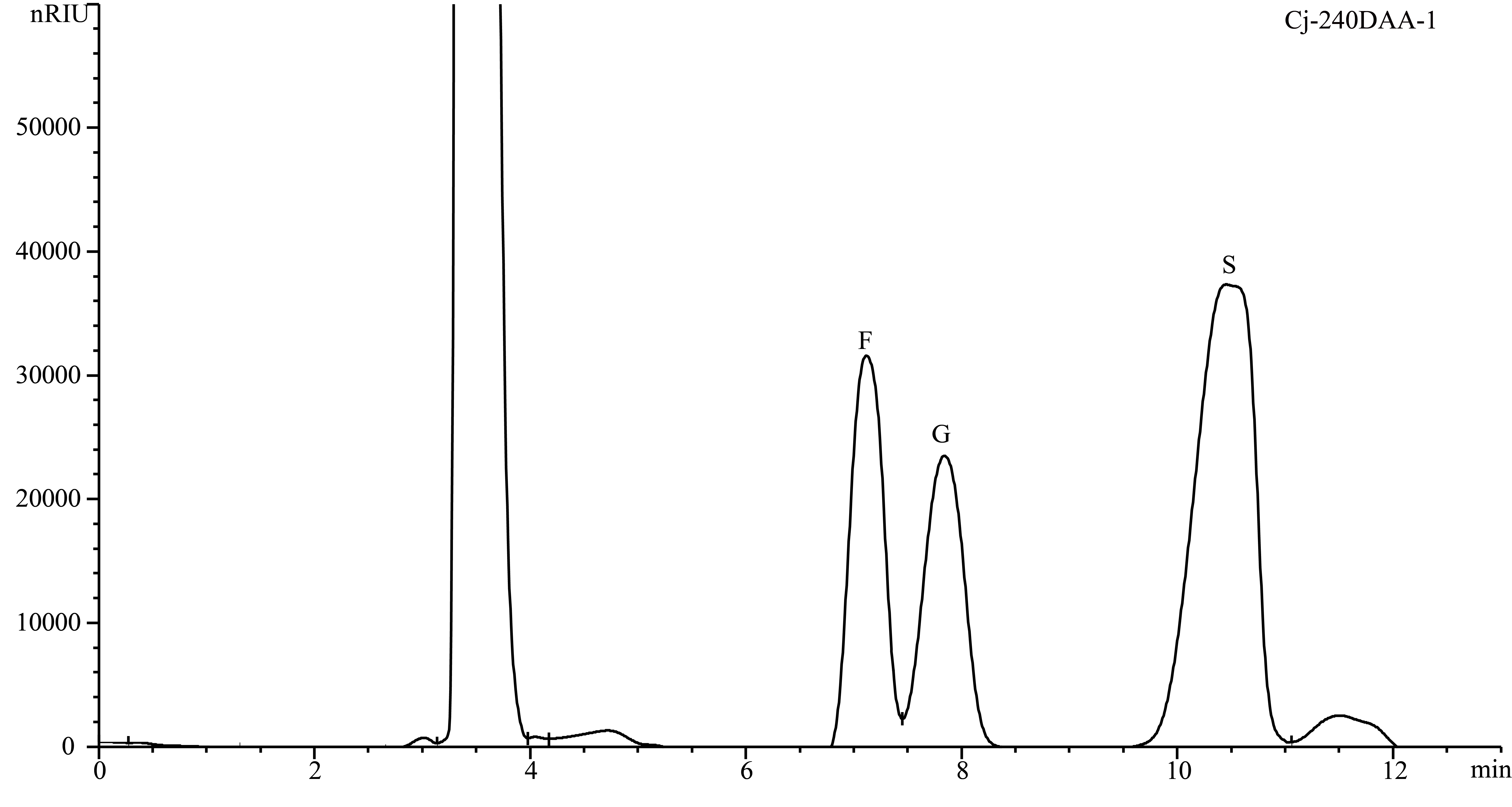

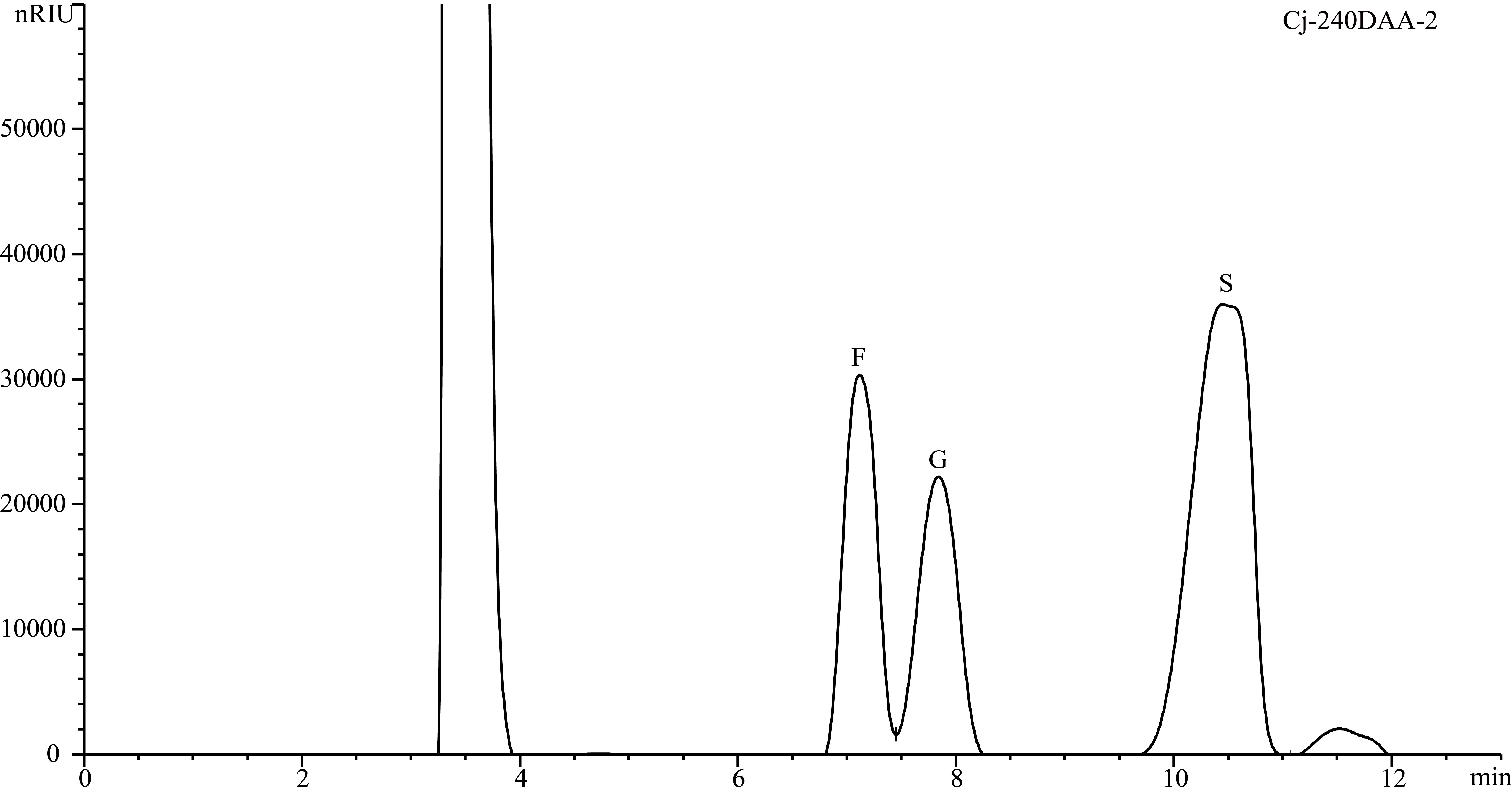

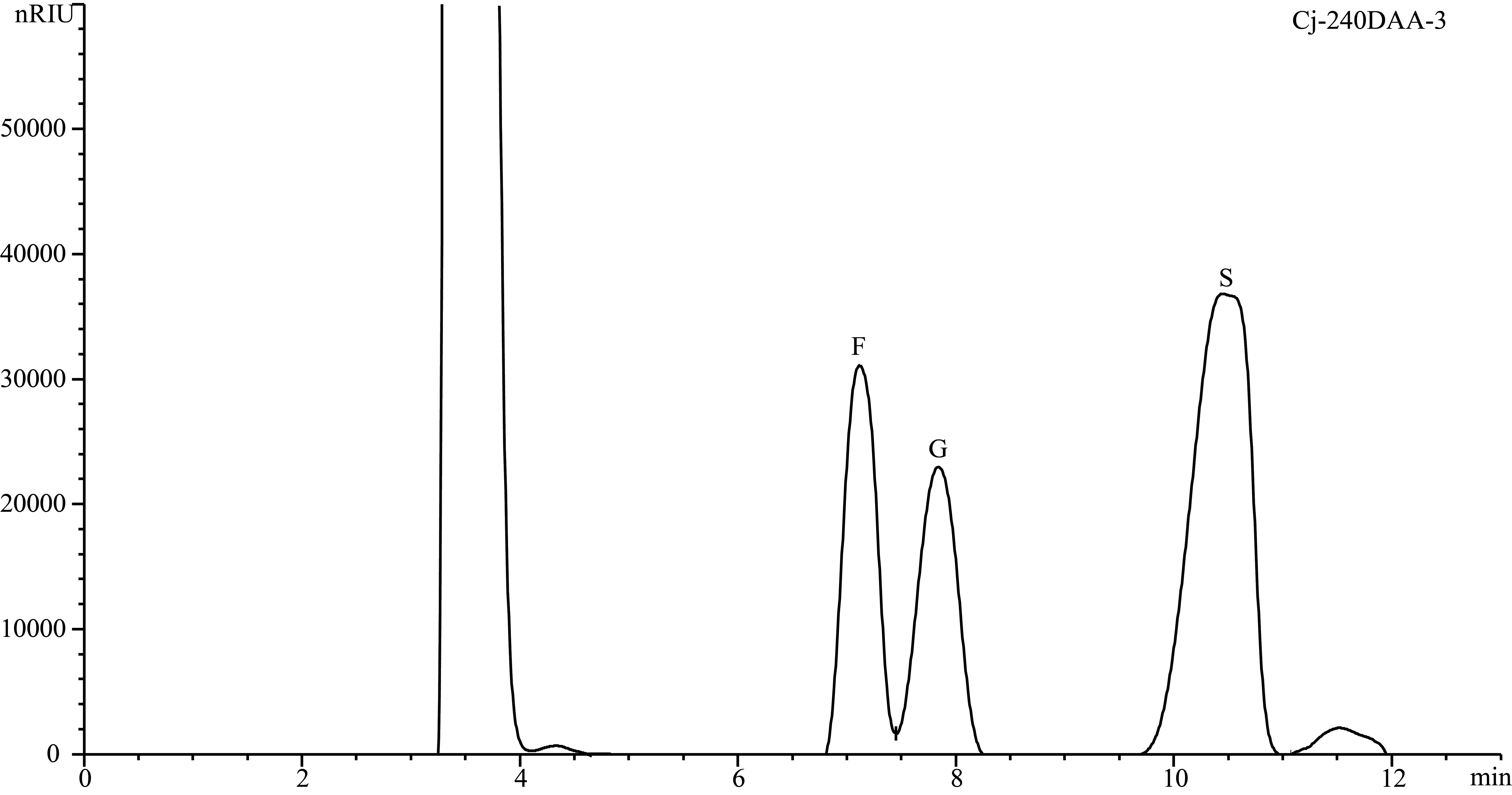

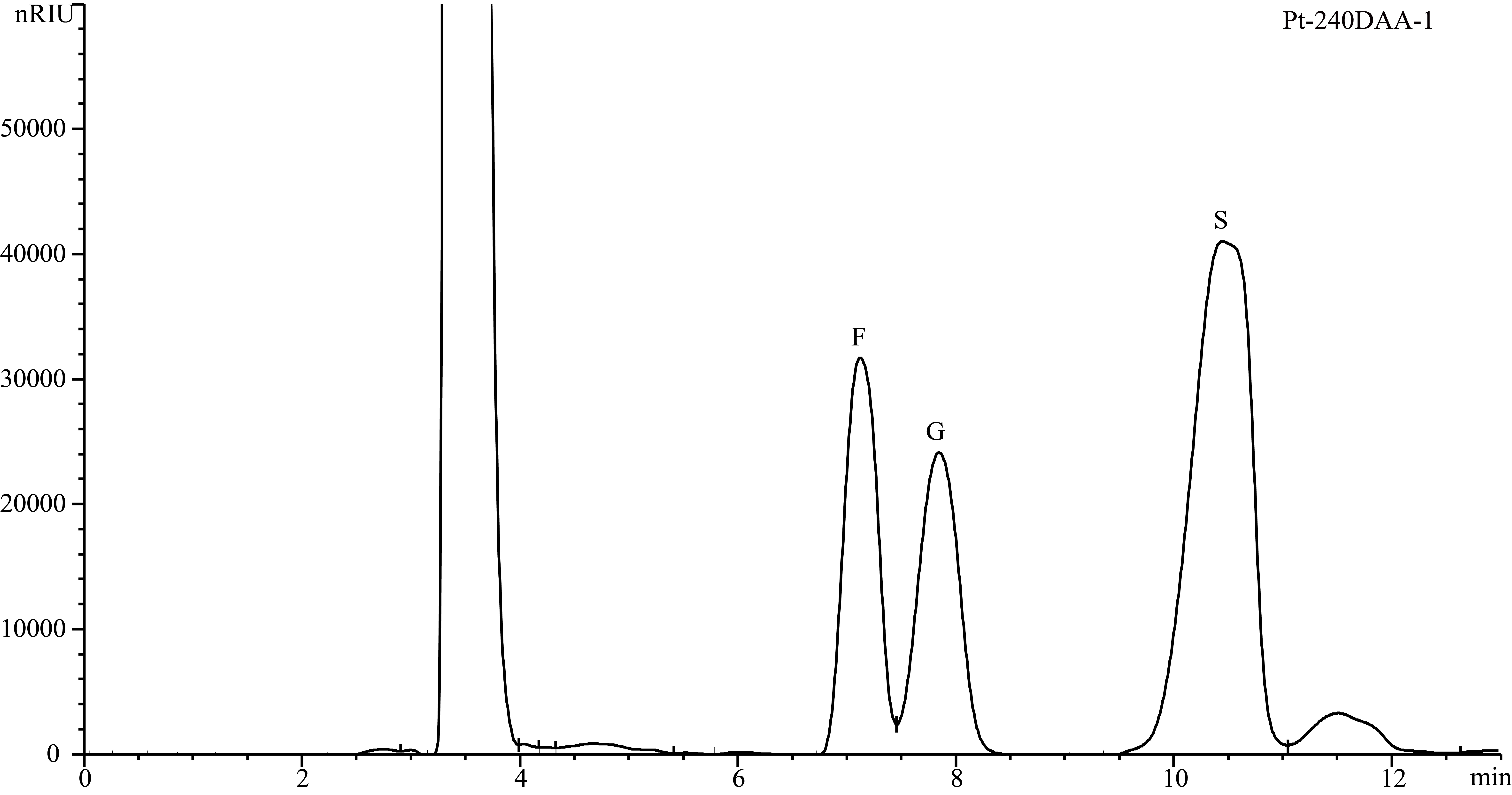

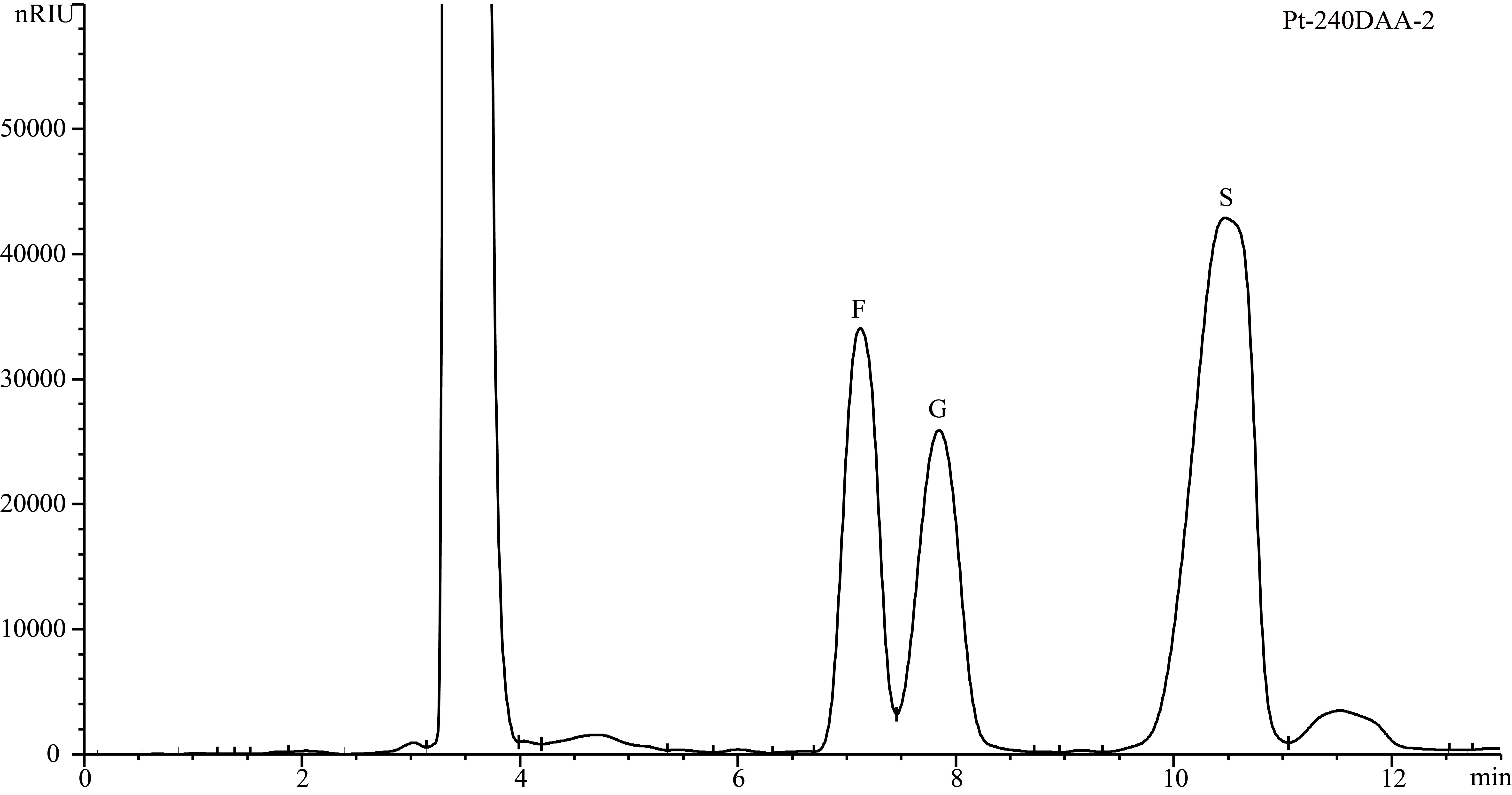

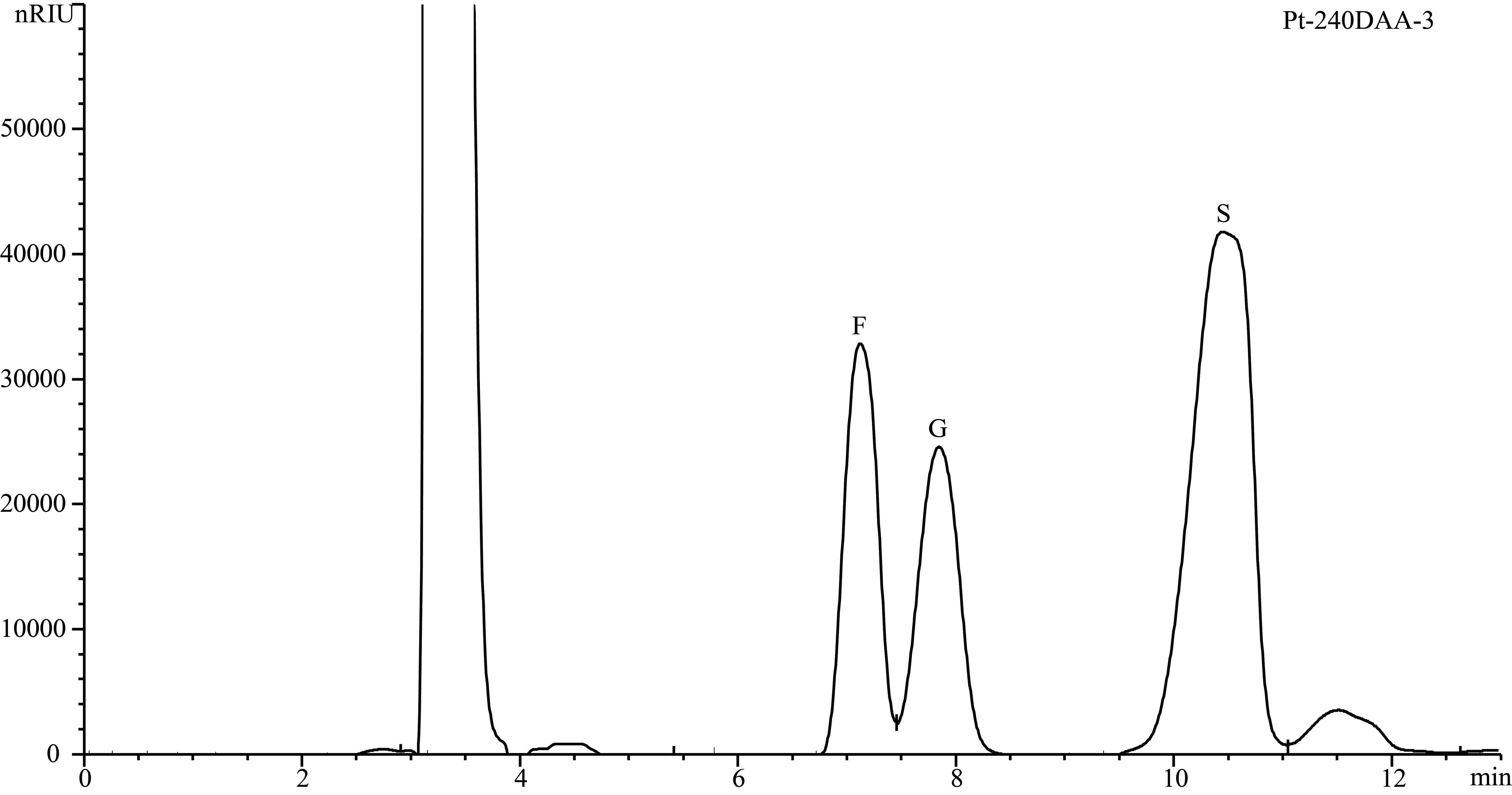

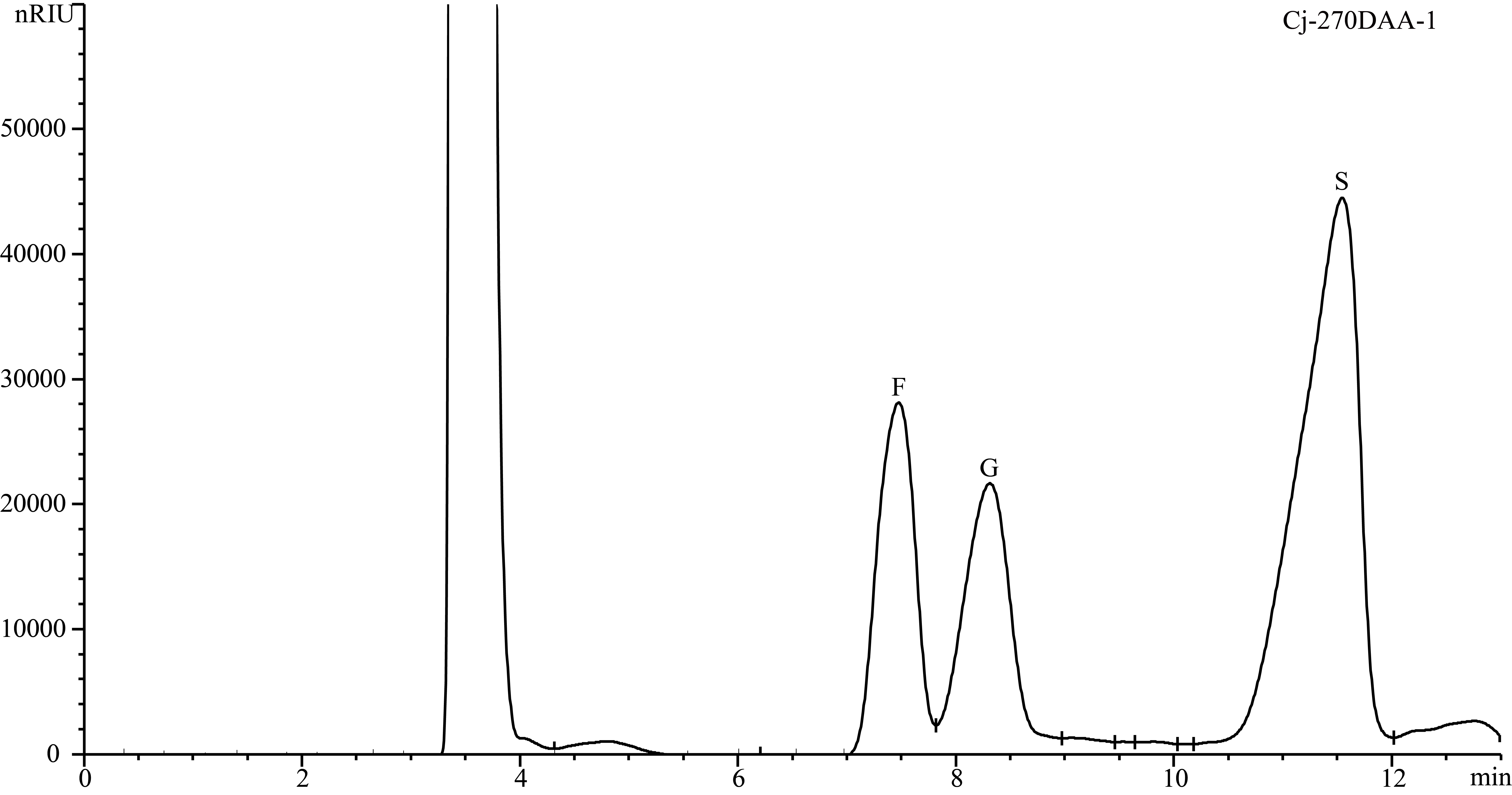

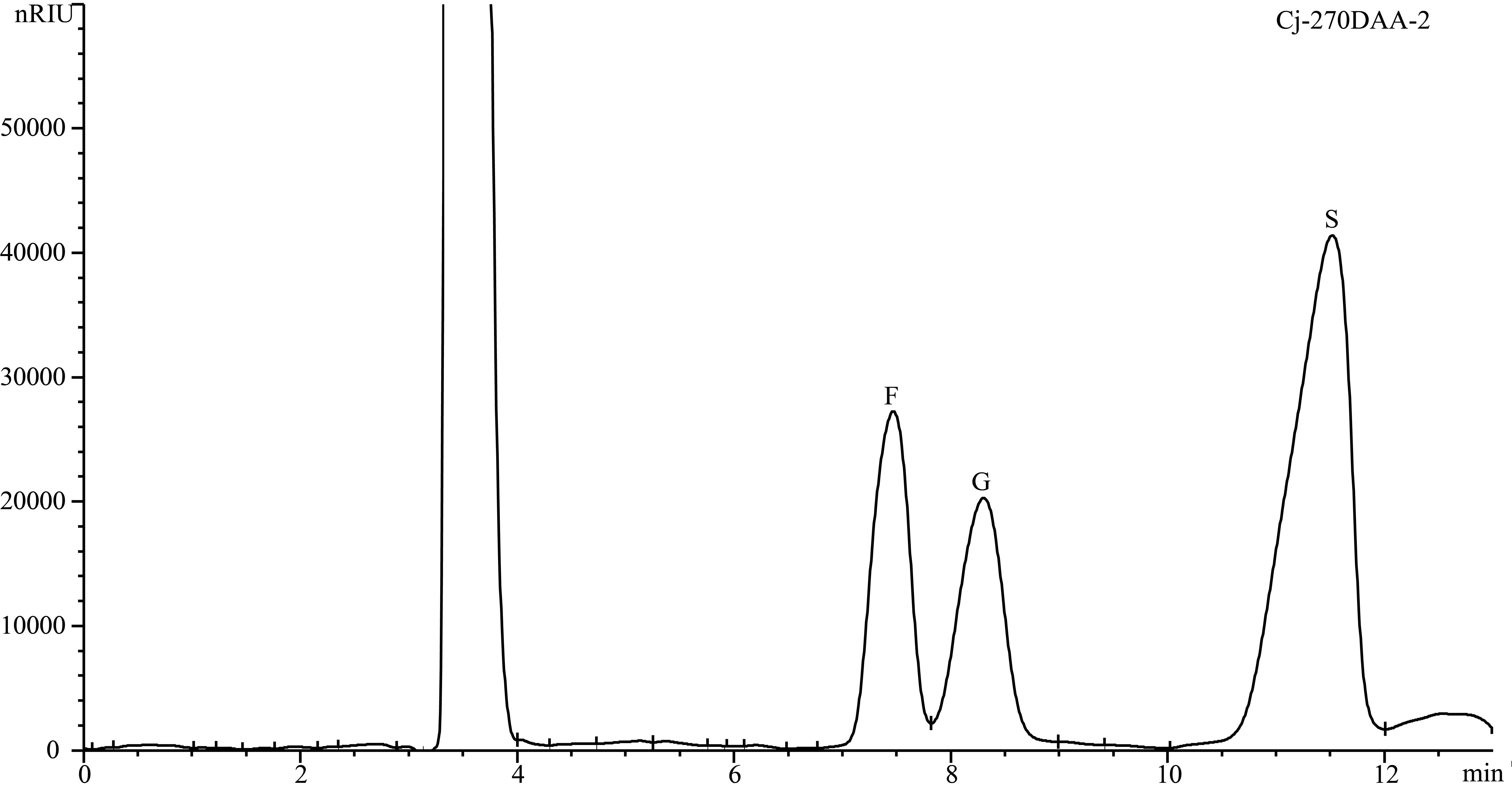

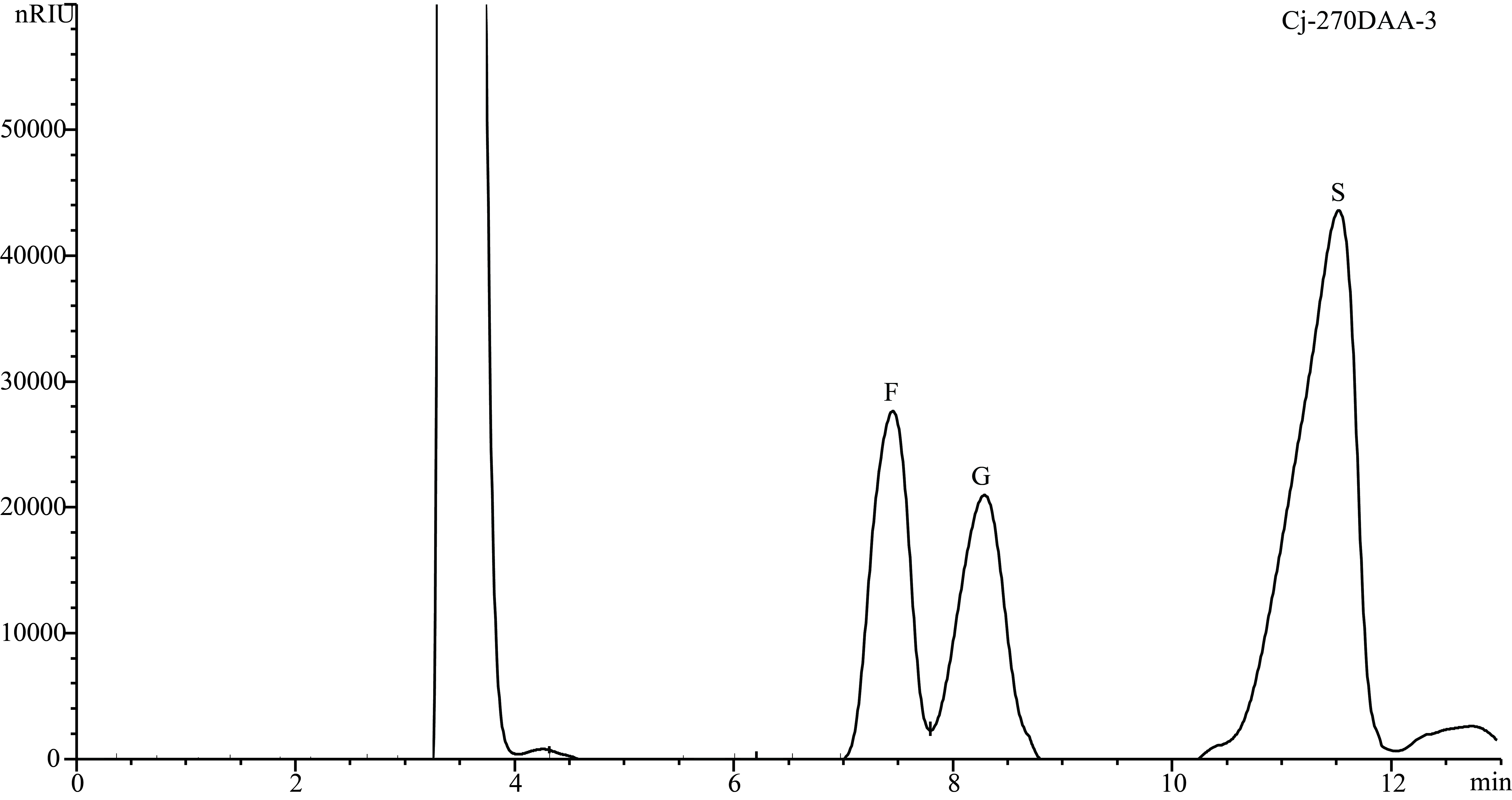

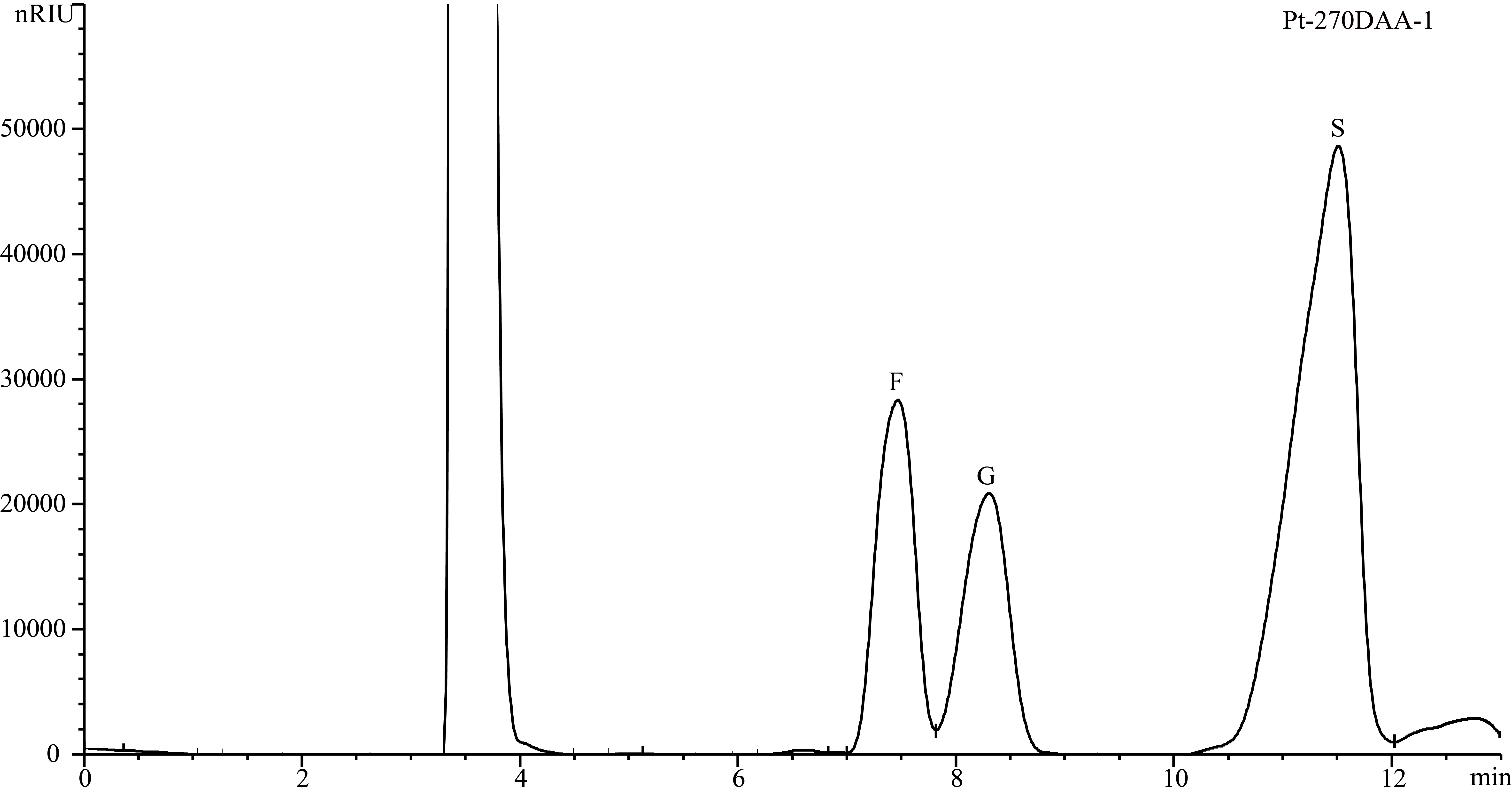

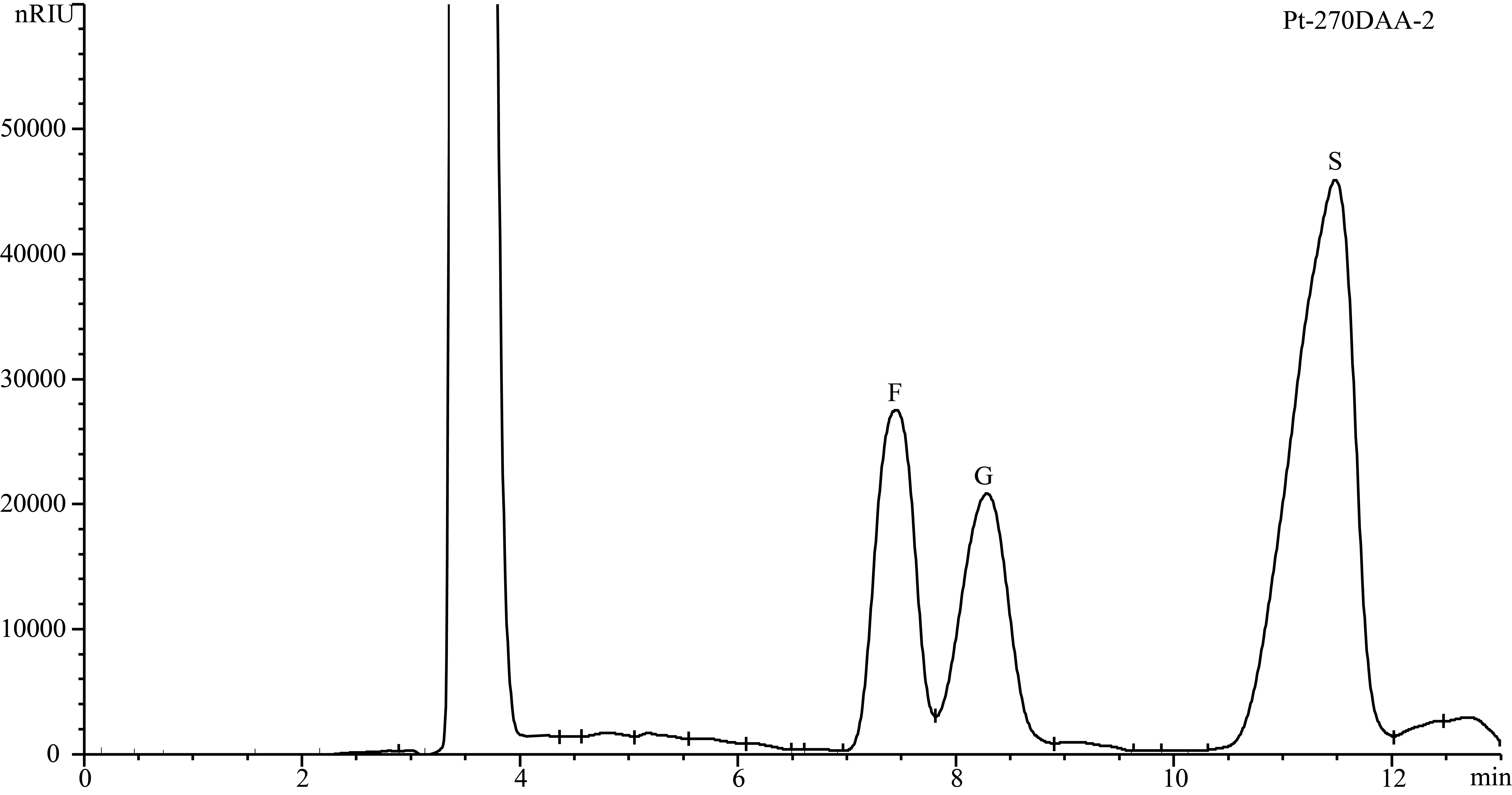

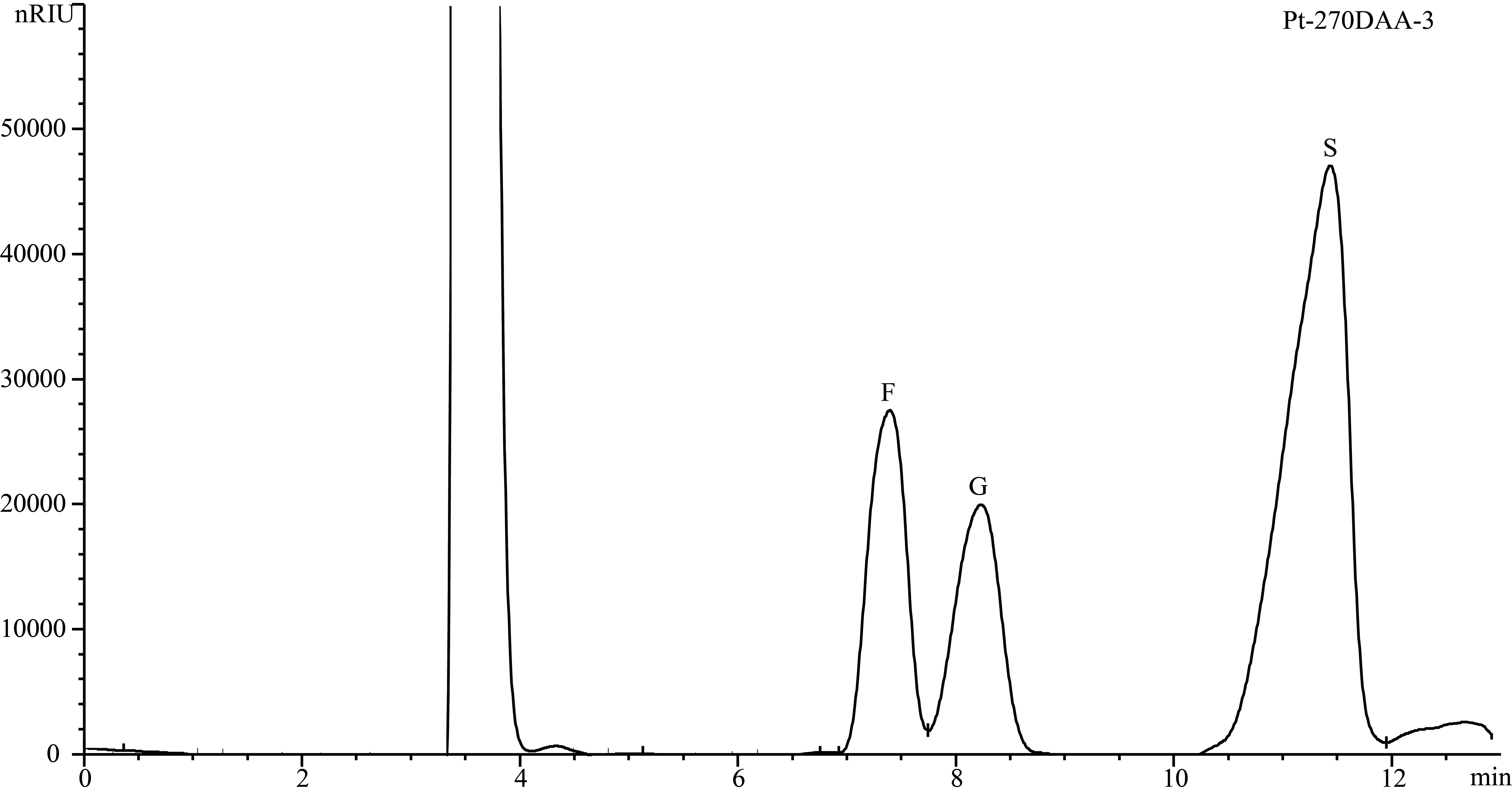

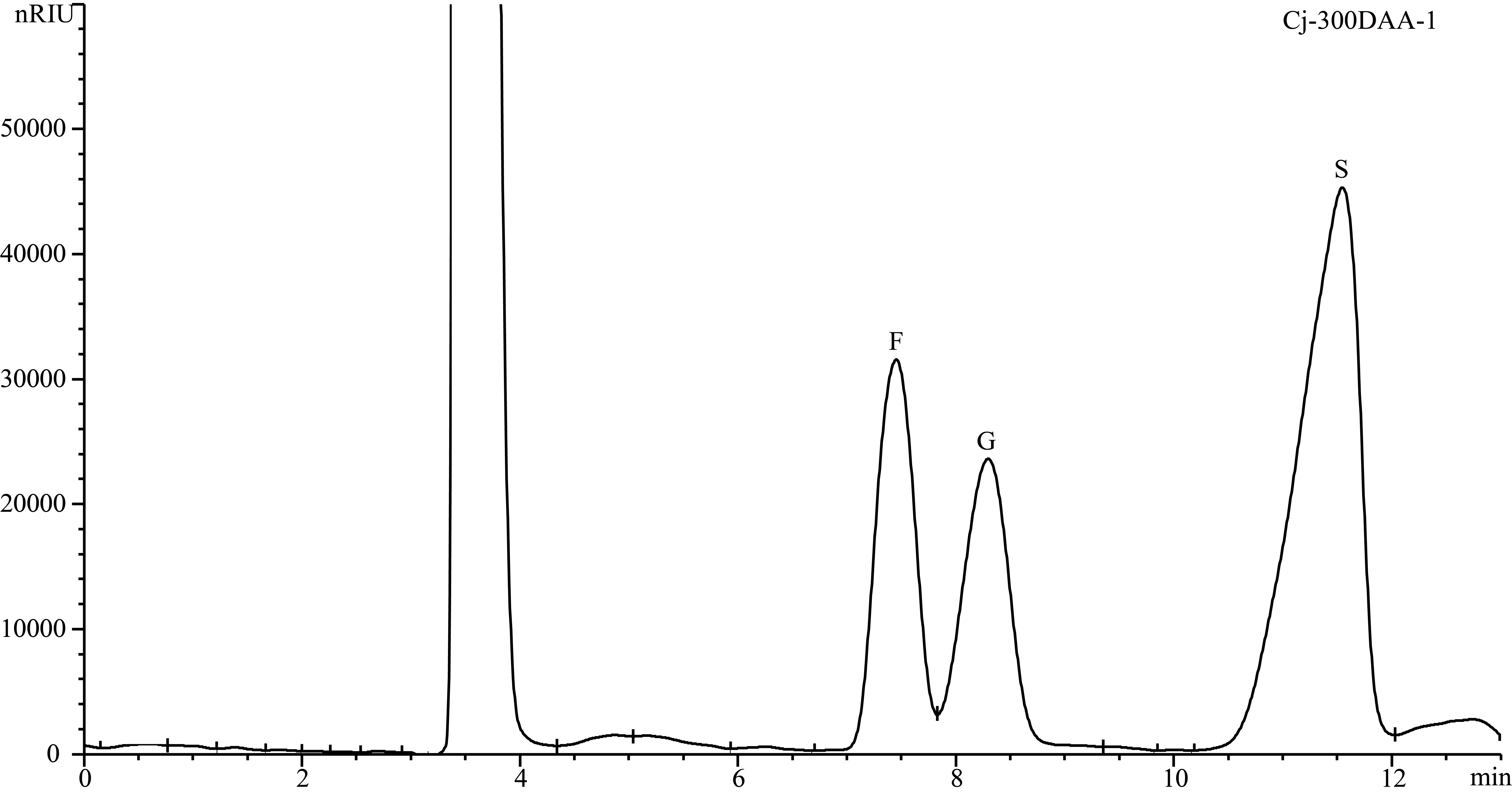

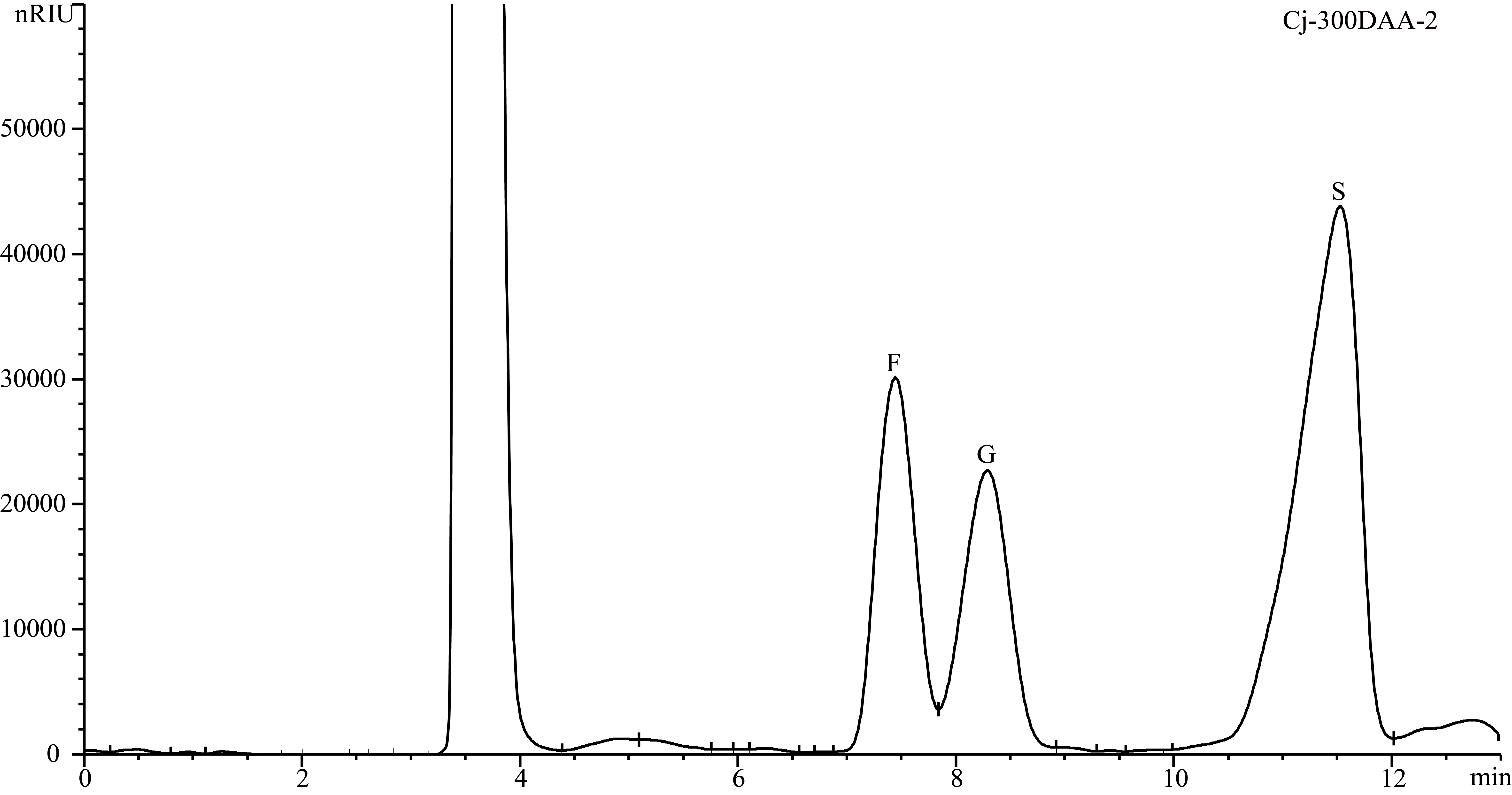

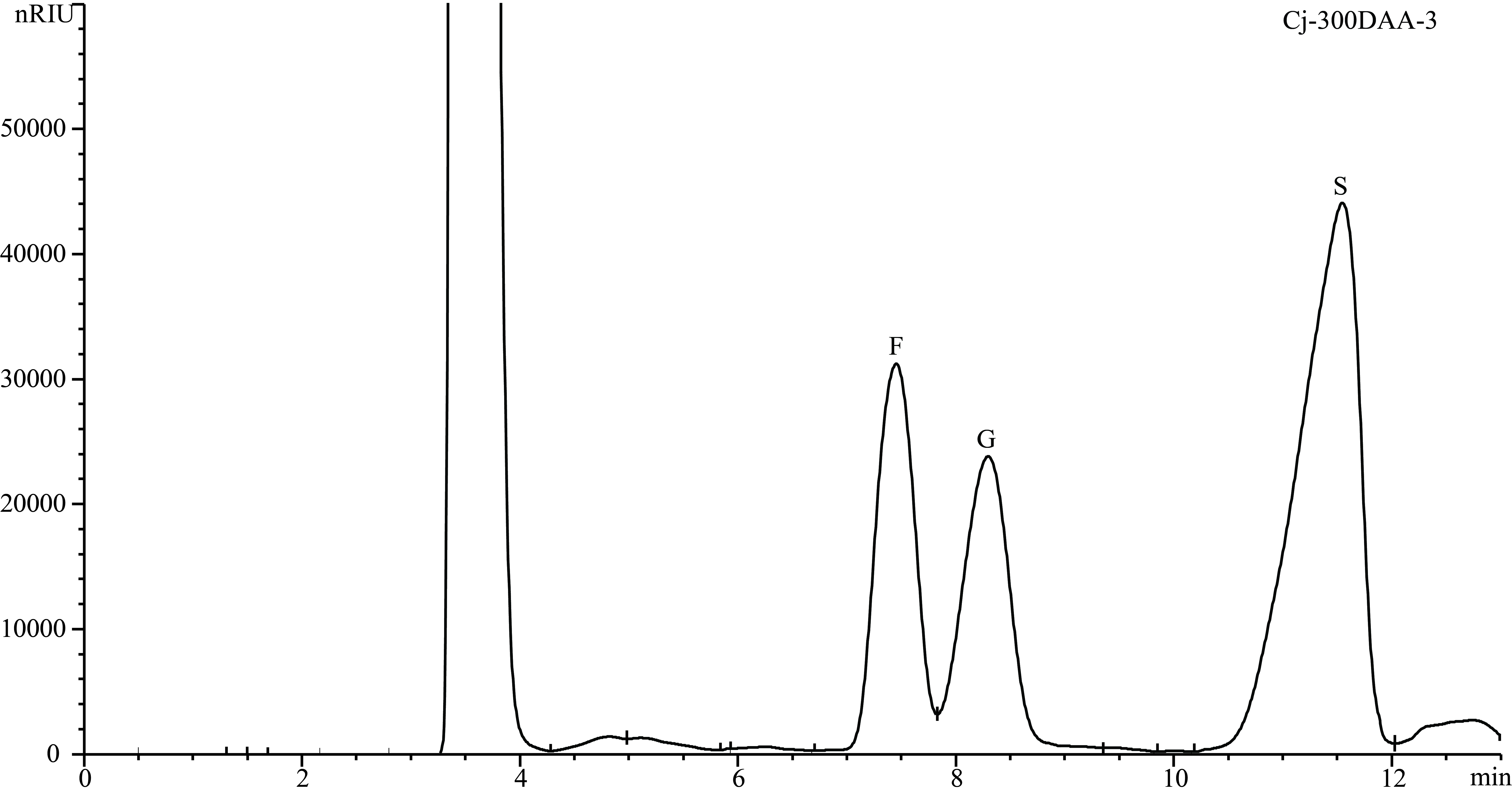

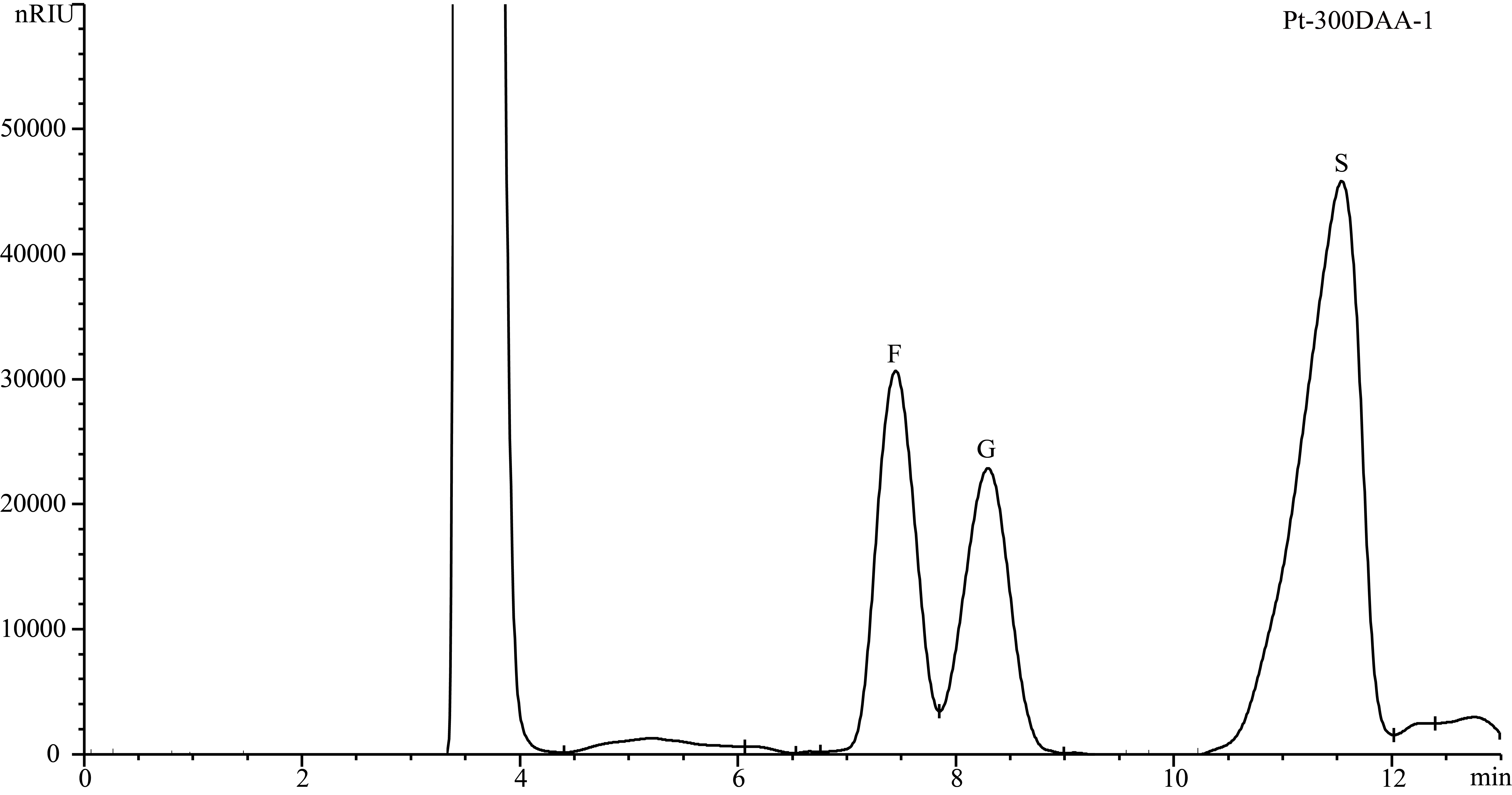

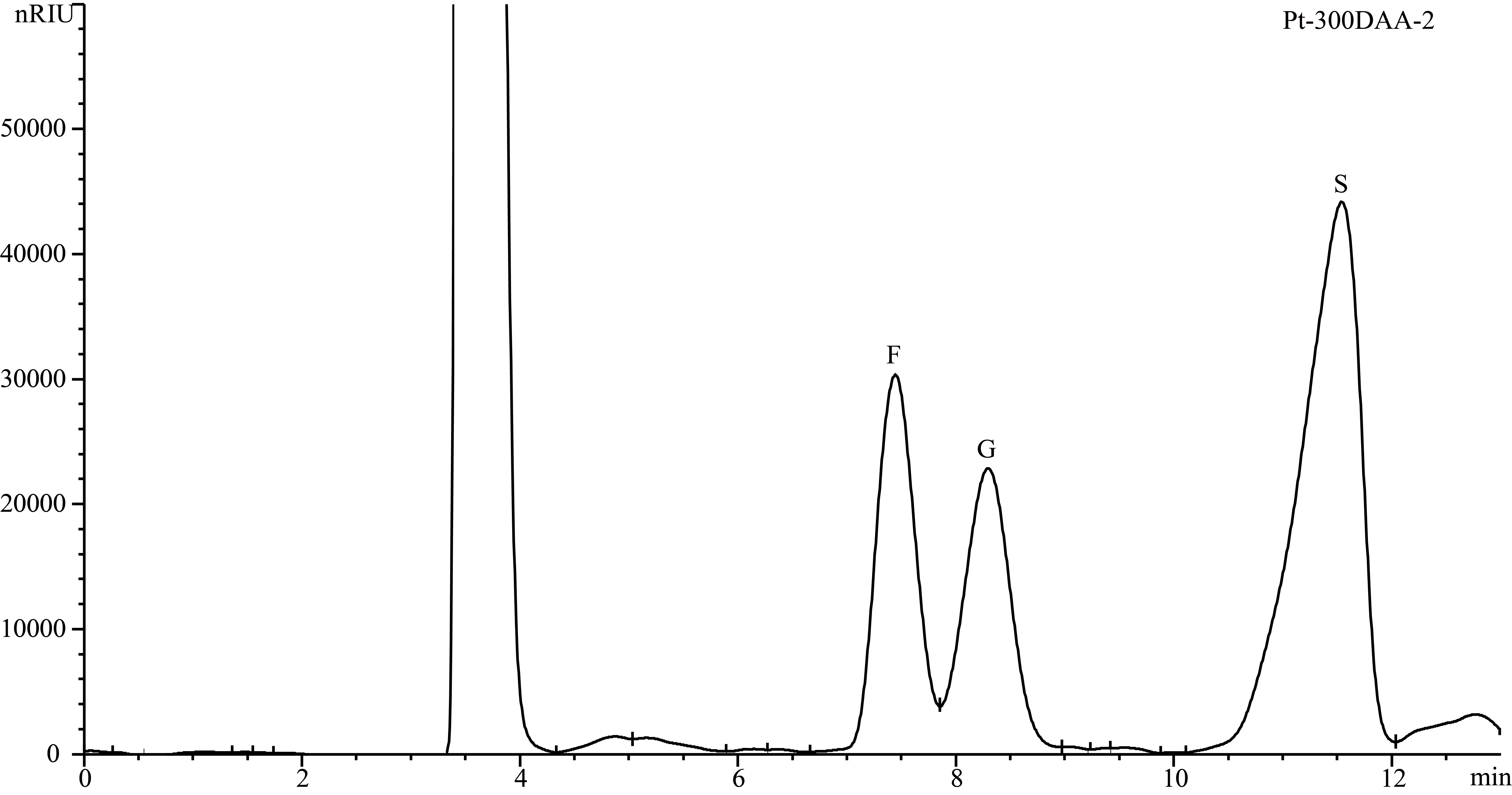

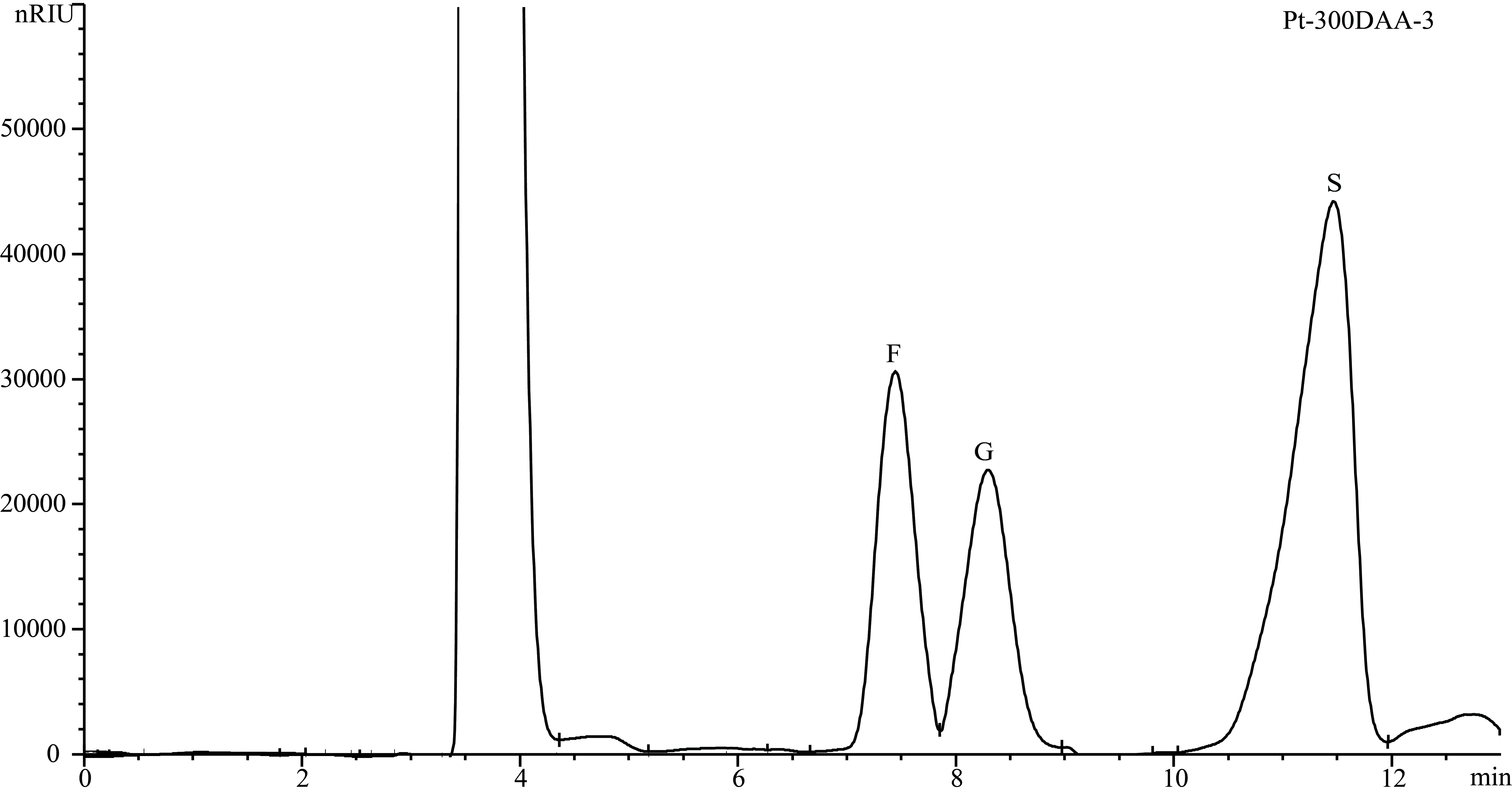


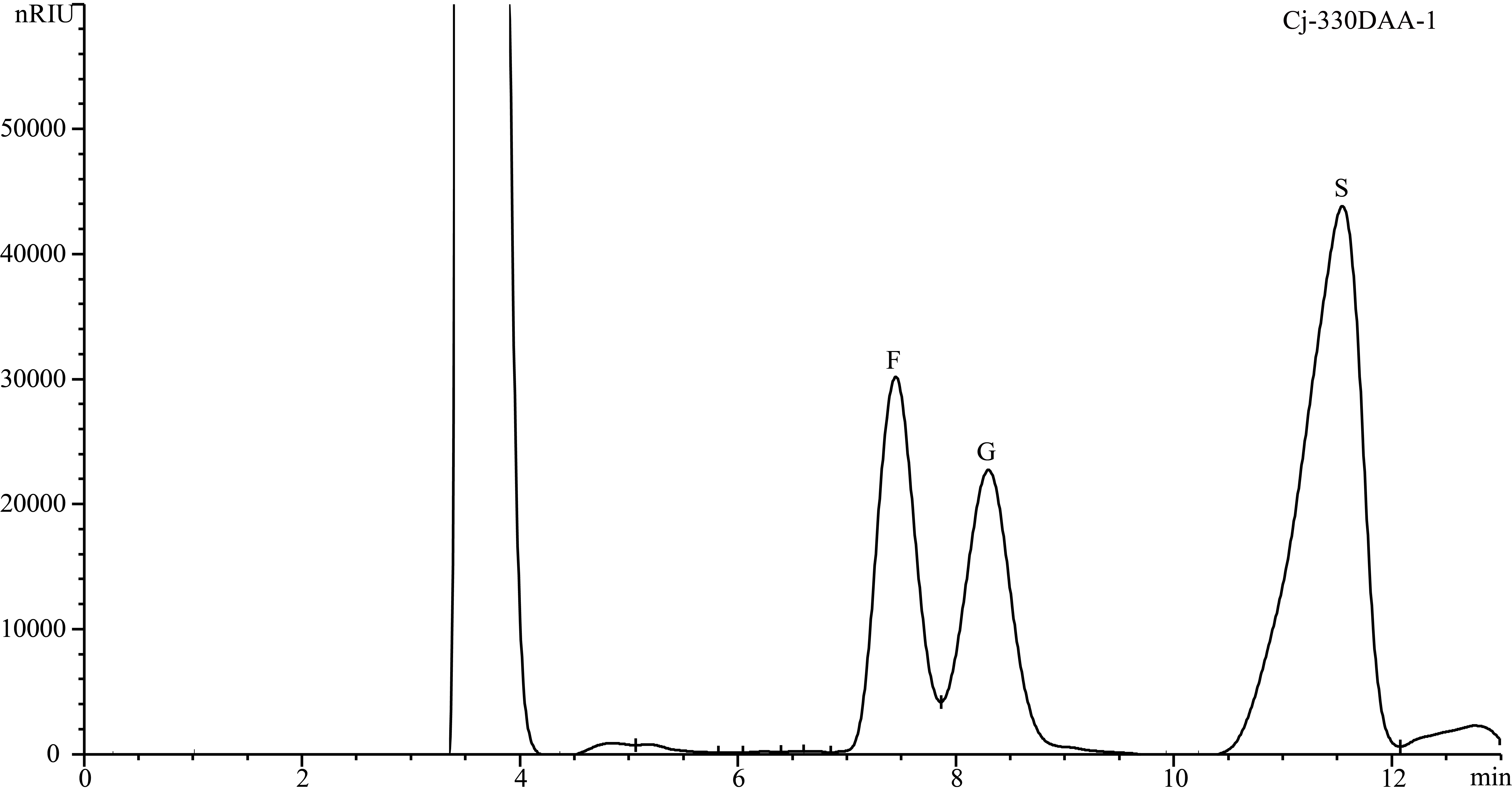

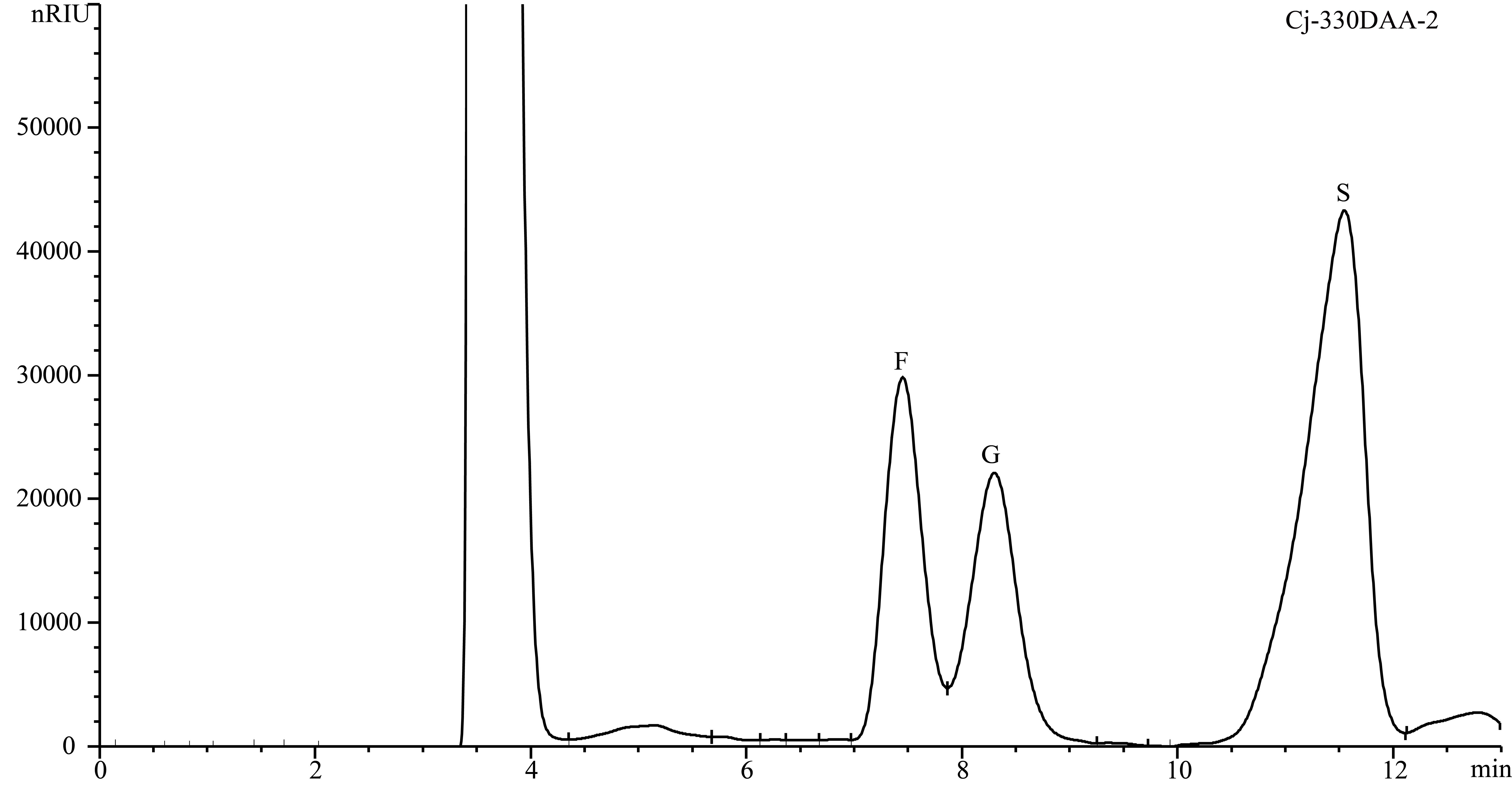

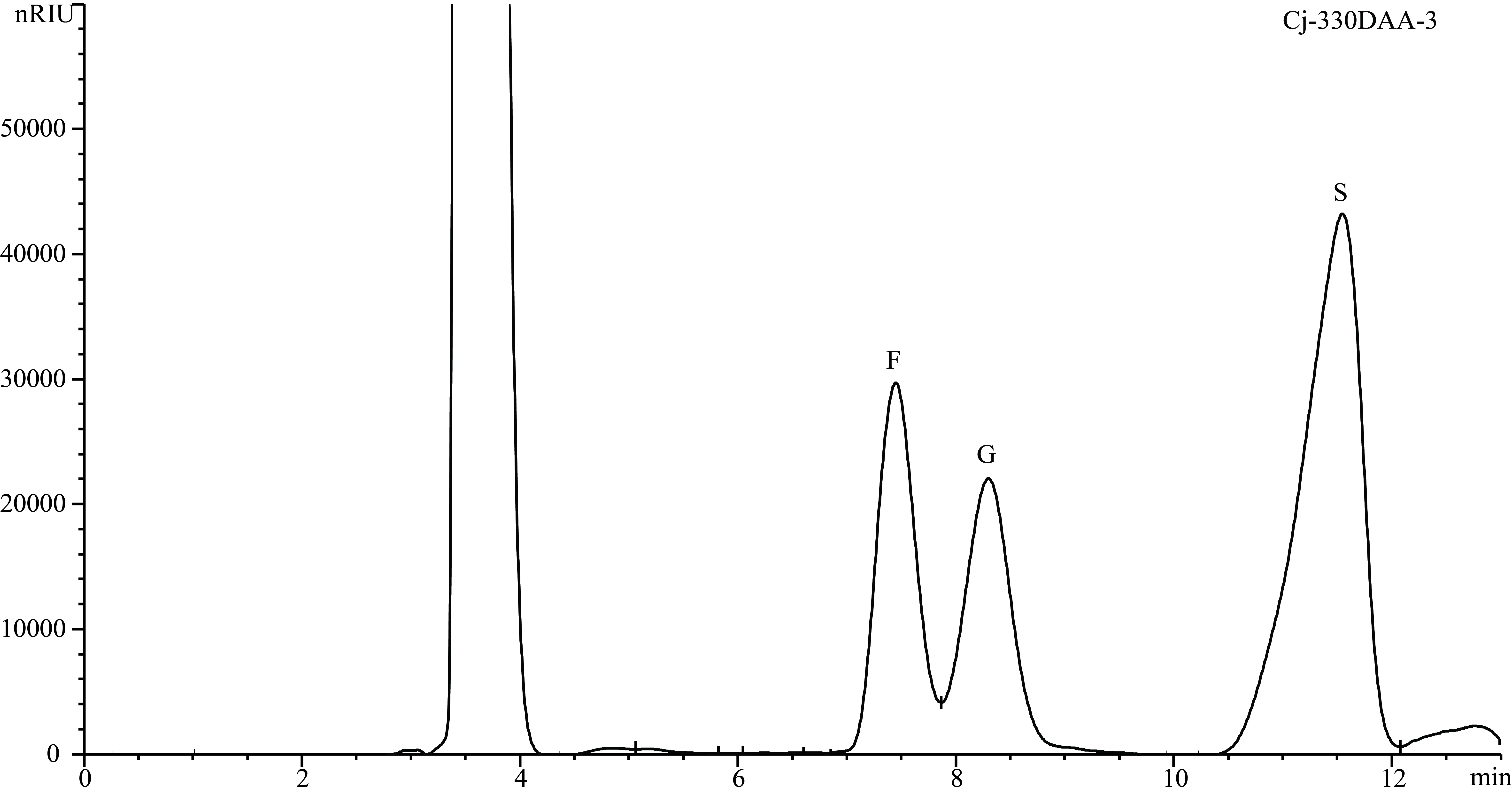


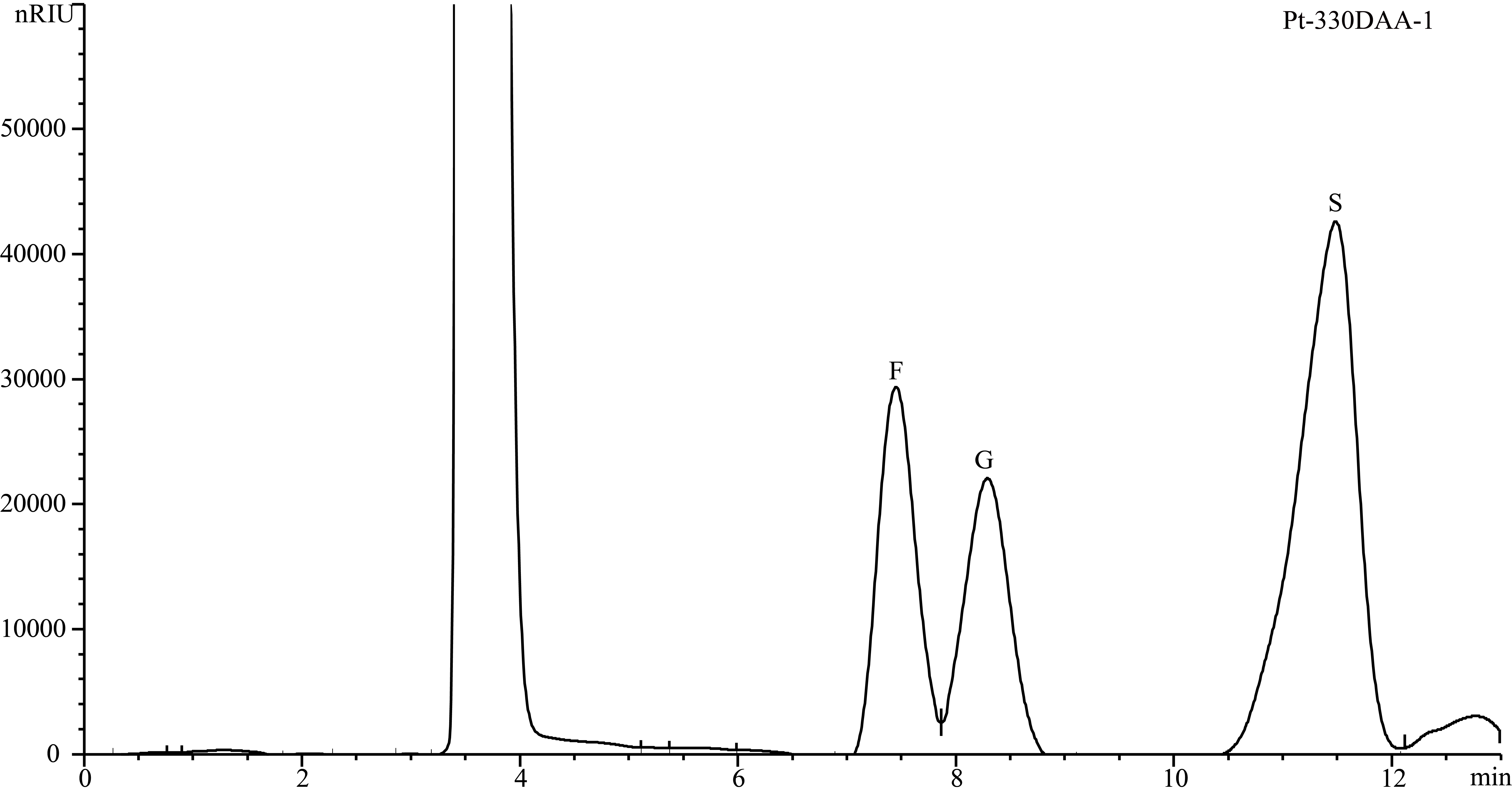

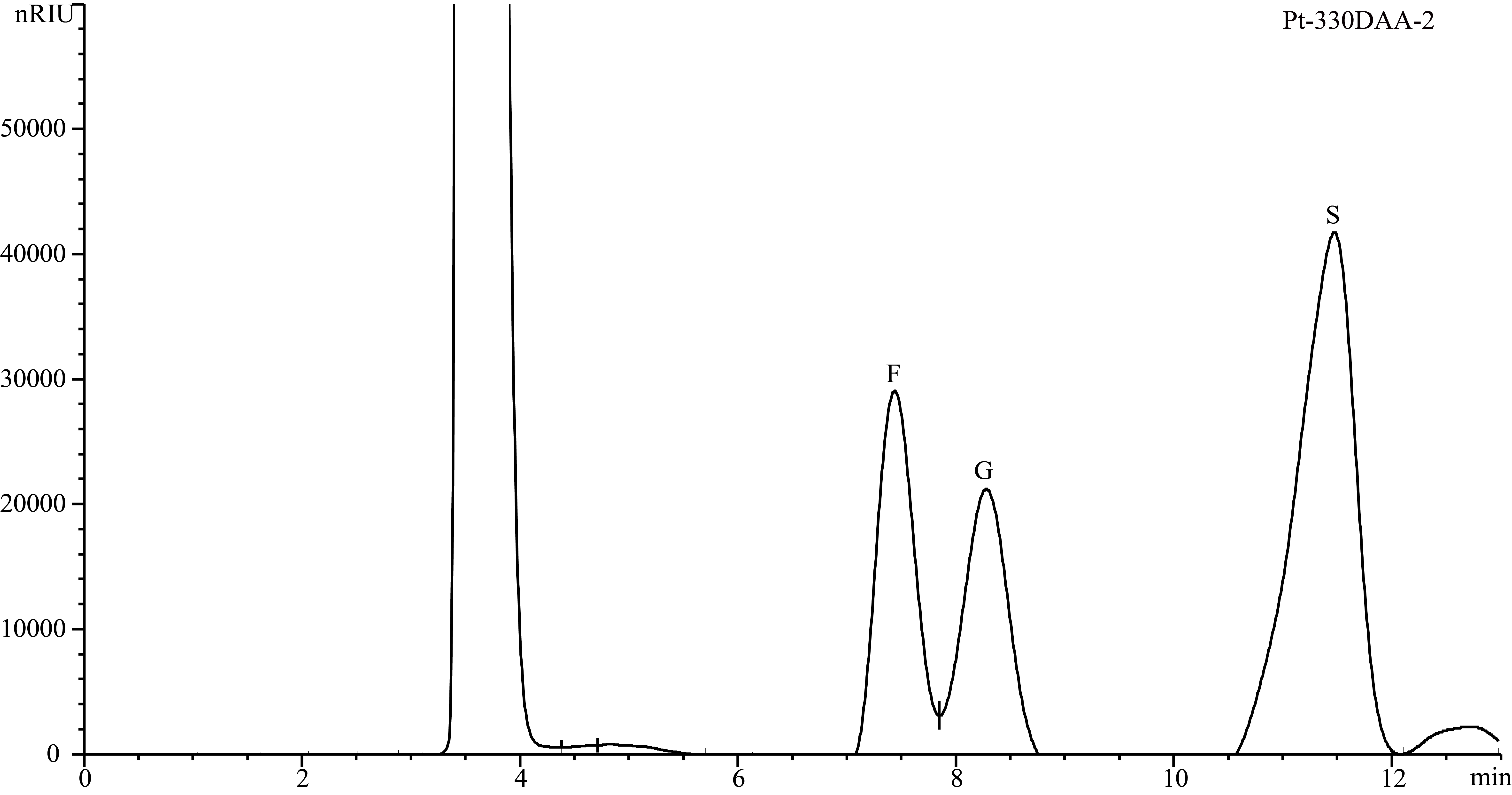

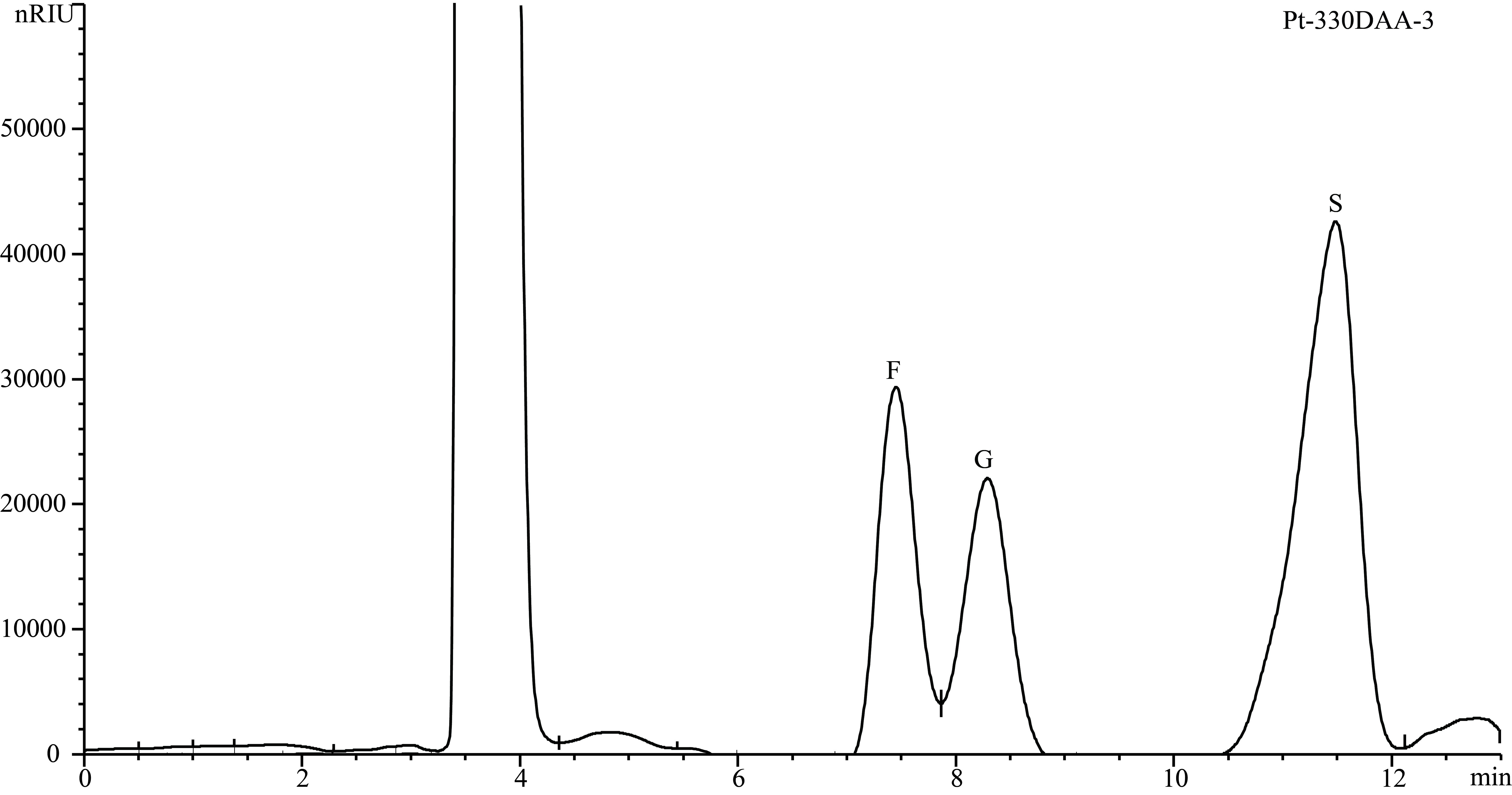


Figure S2. The liquid chromatogram of sample are displayed total of 22 samples with 3 biological replicates. F presents fructose, G is glucose, and S is sucrose.

Figure S3.


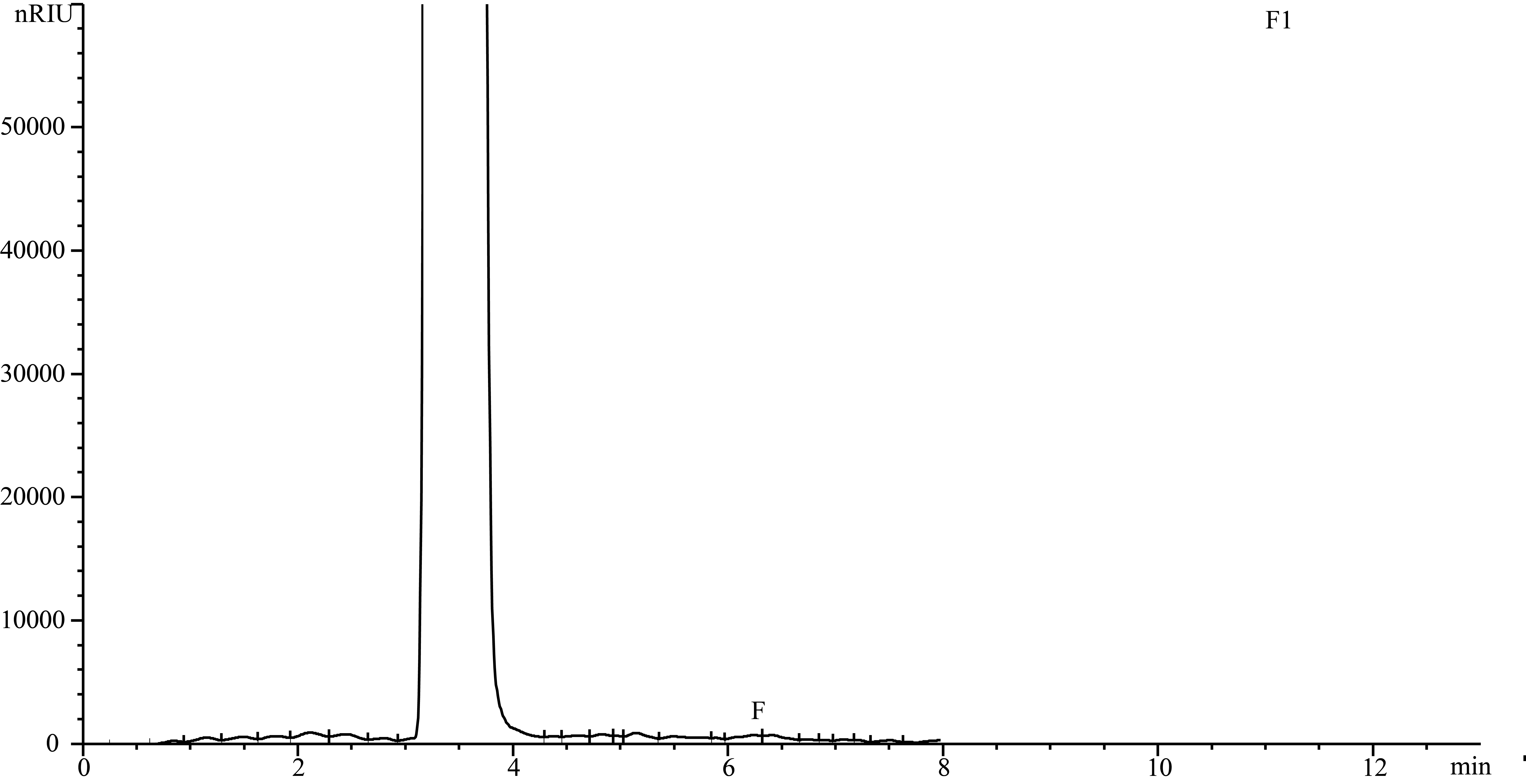

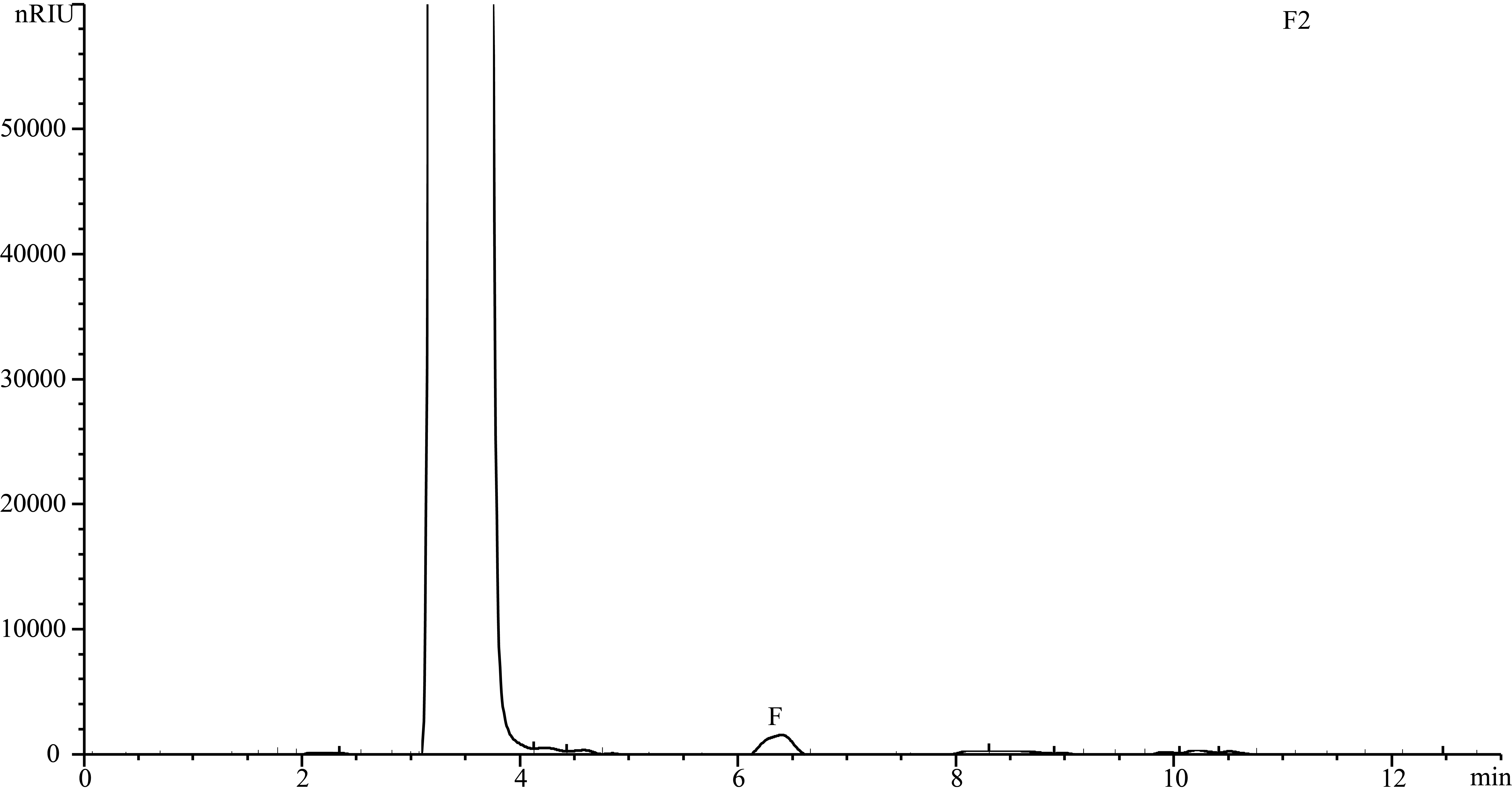

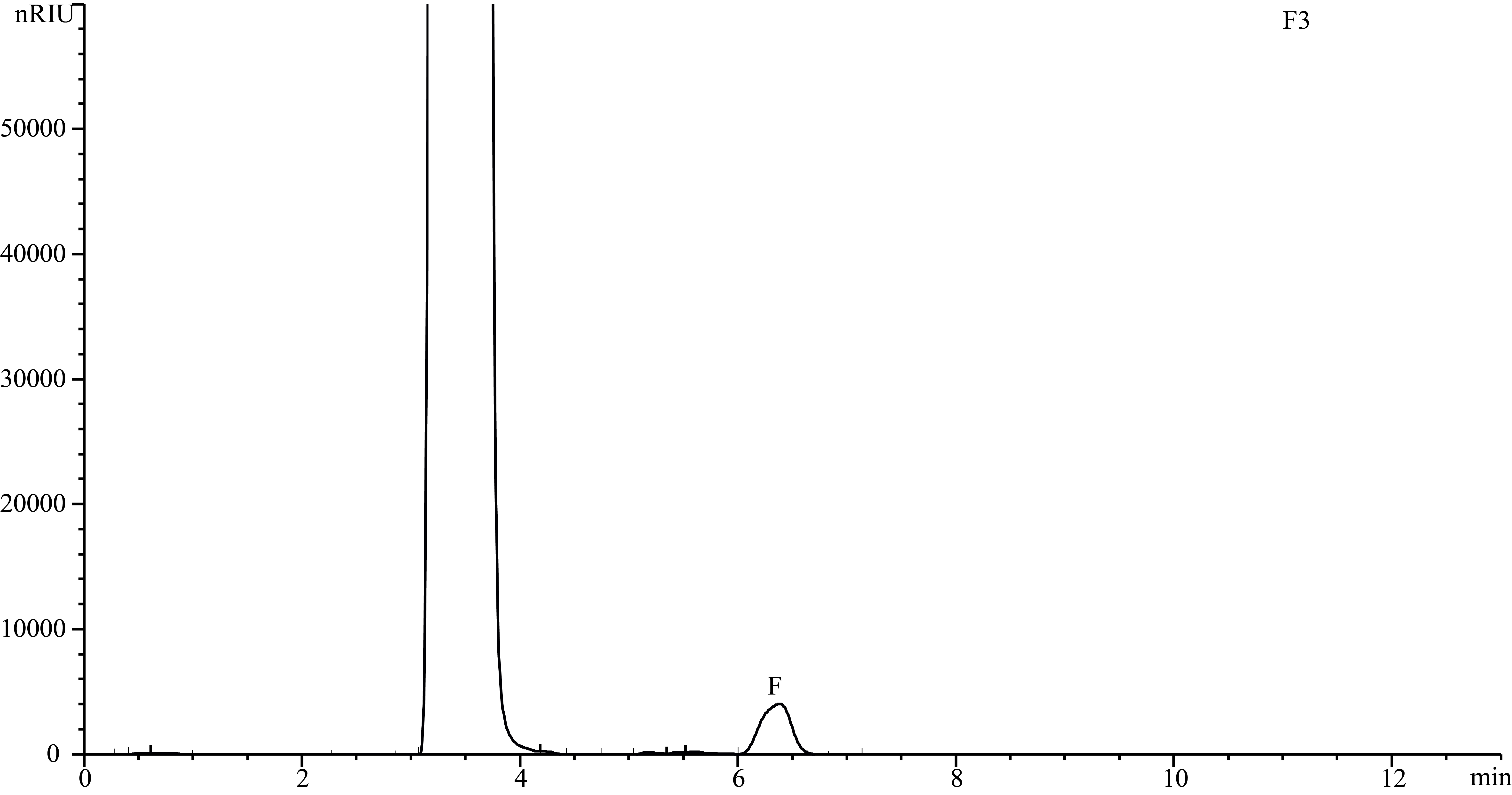


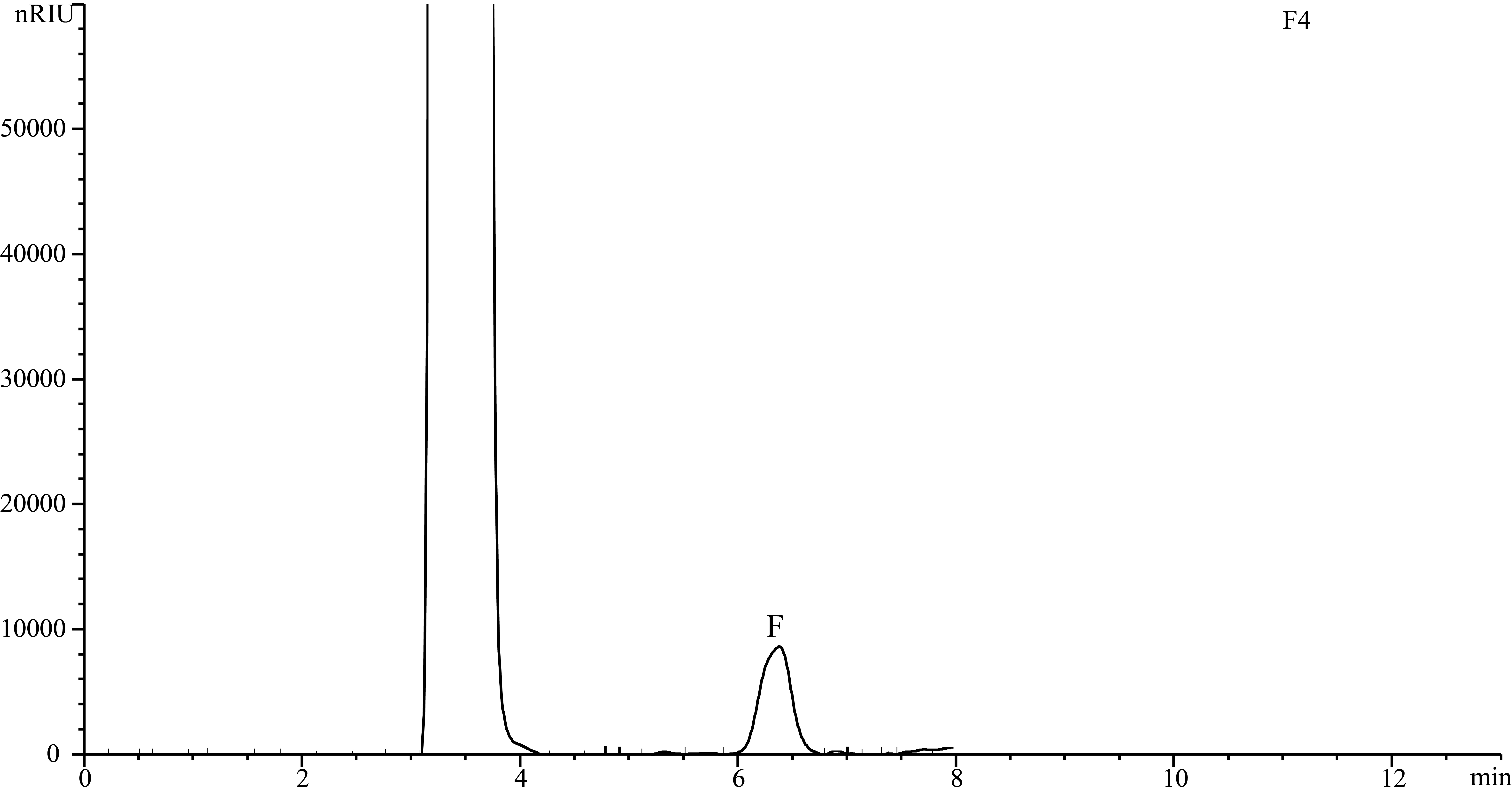

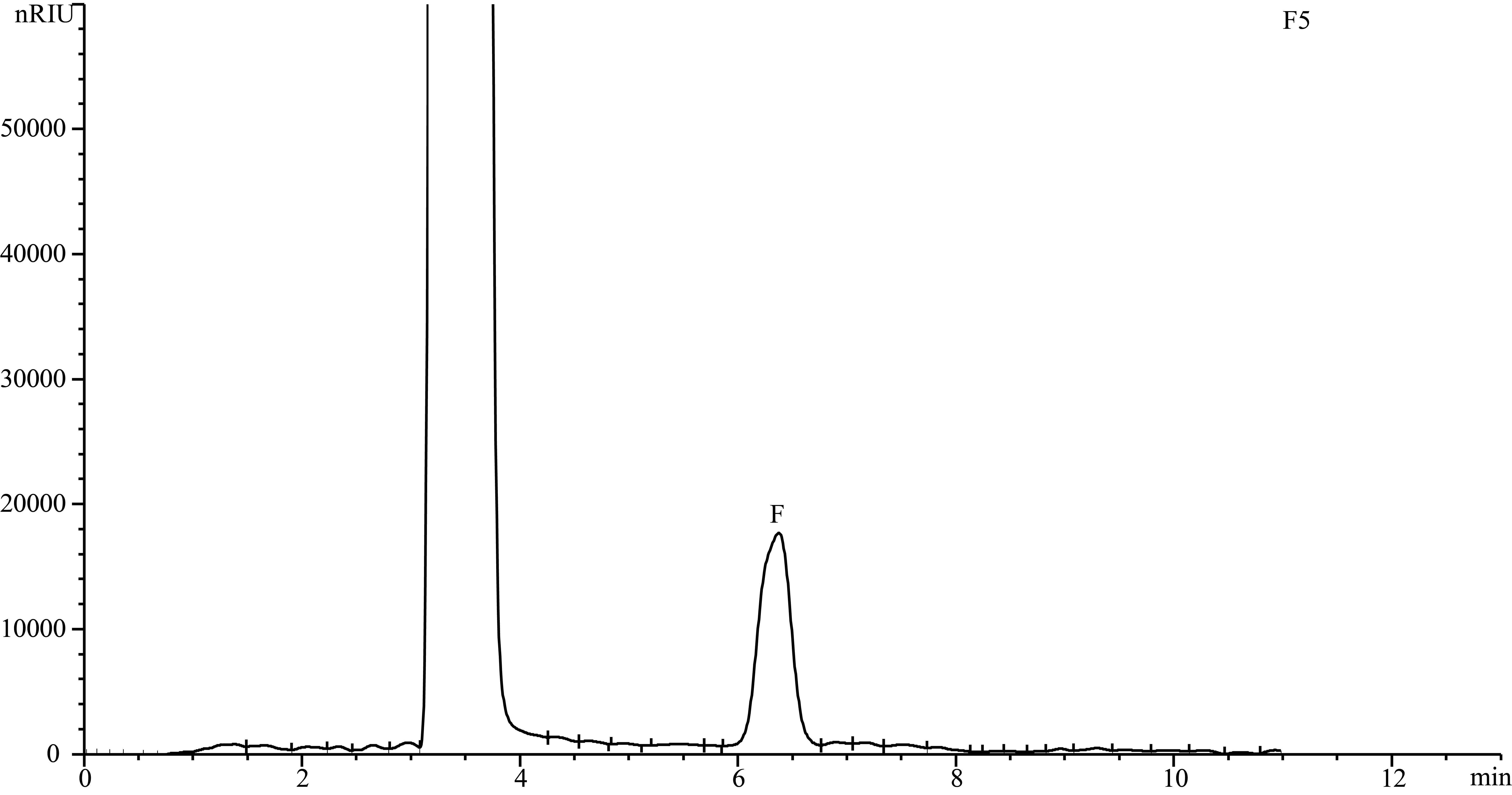

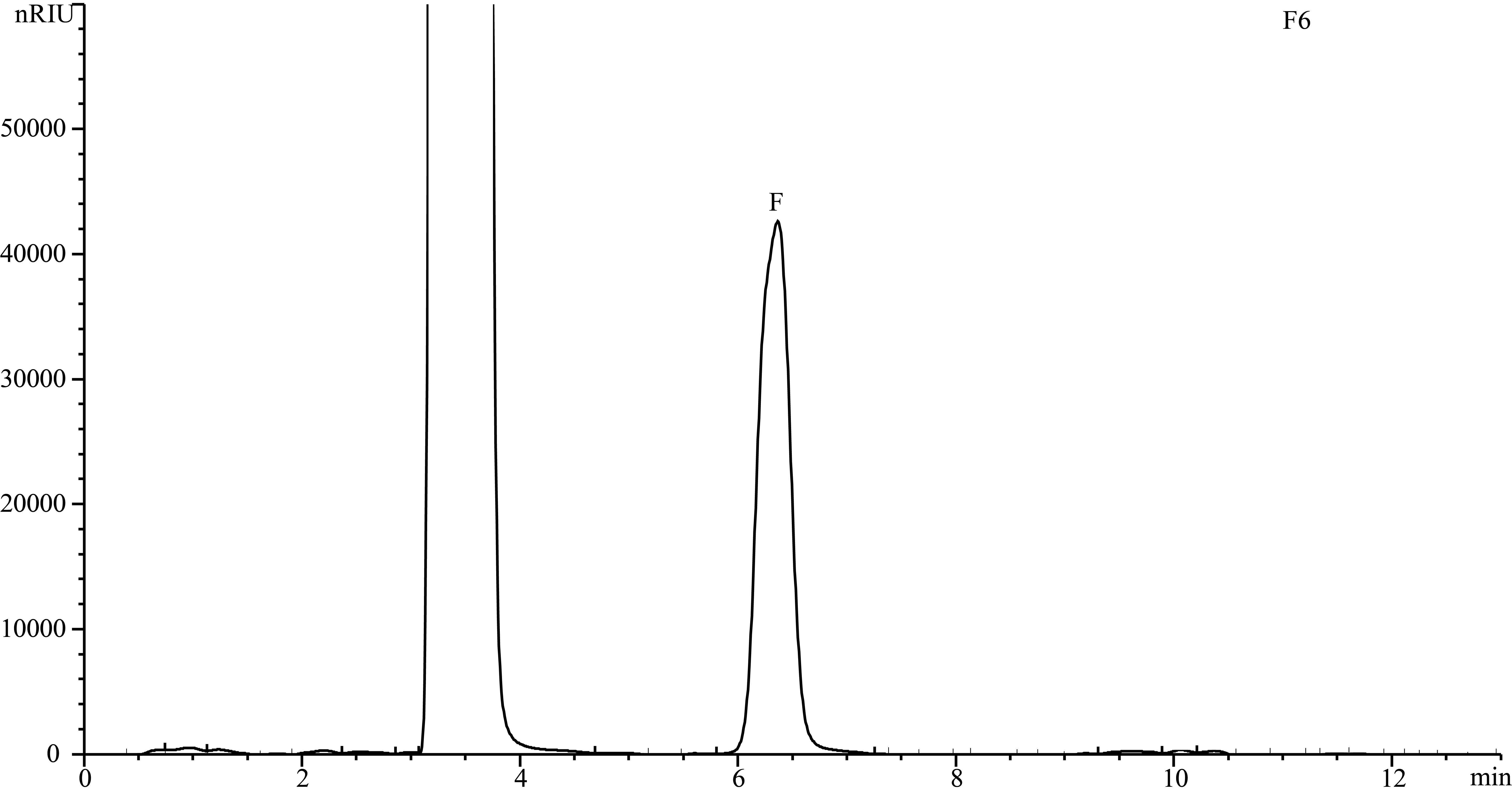


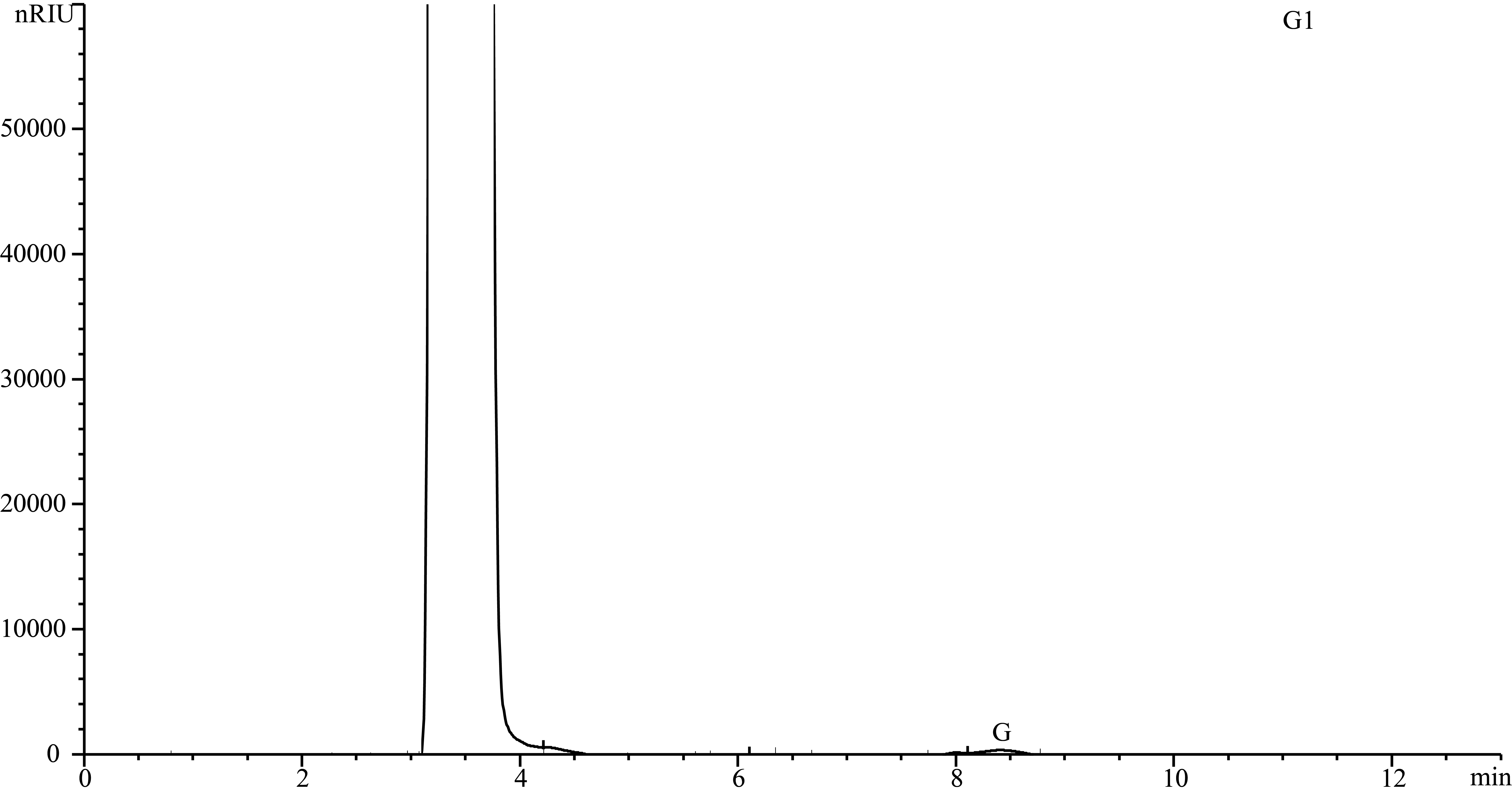

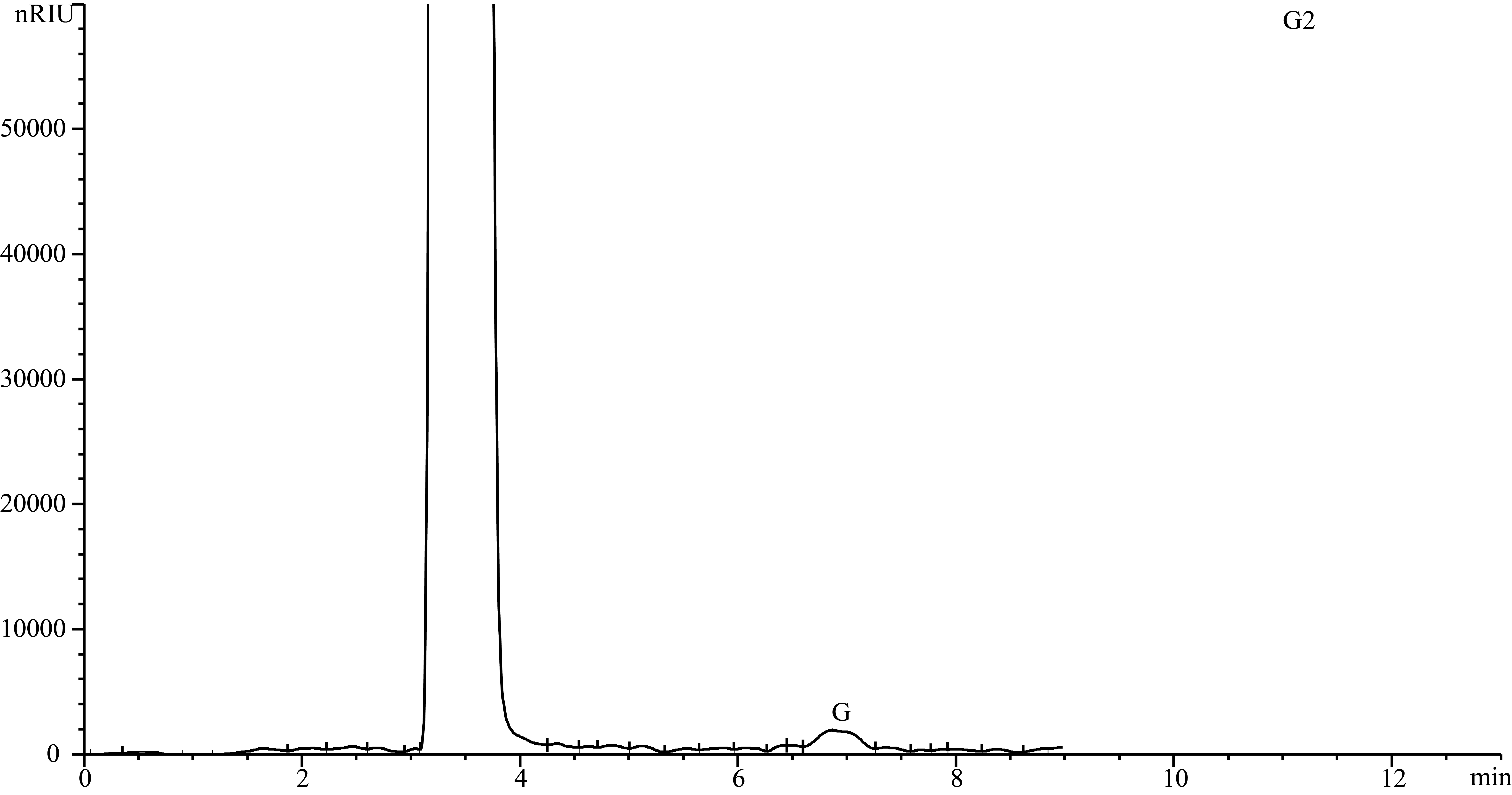

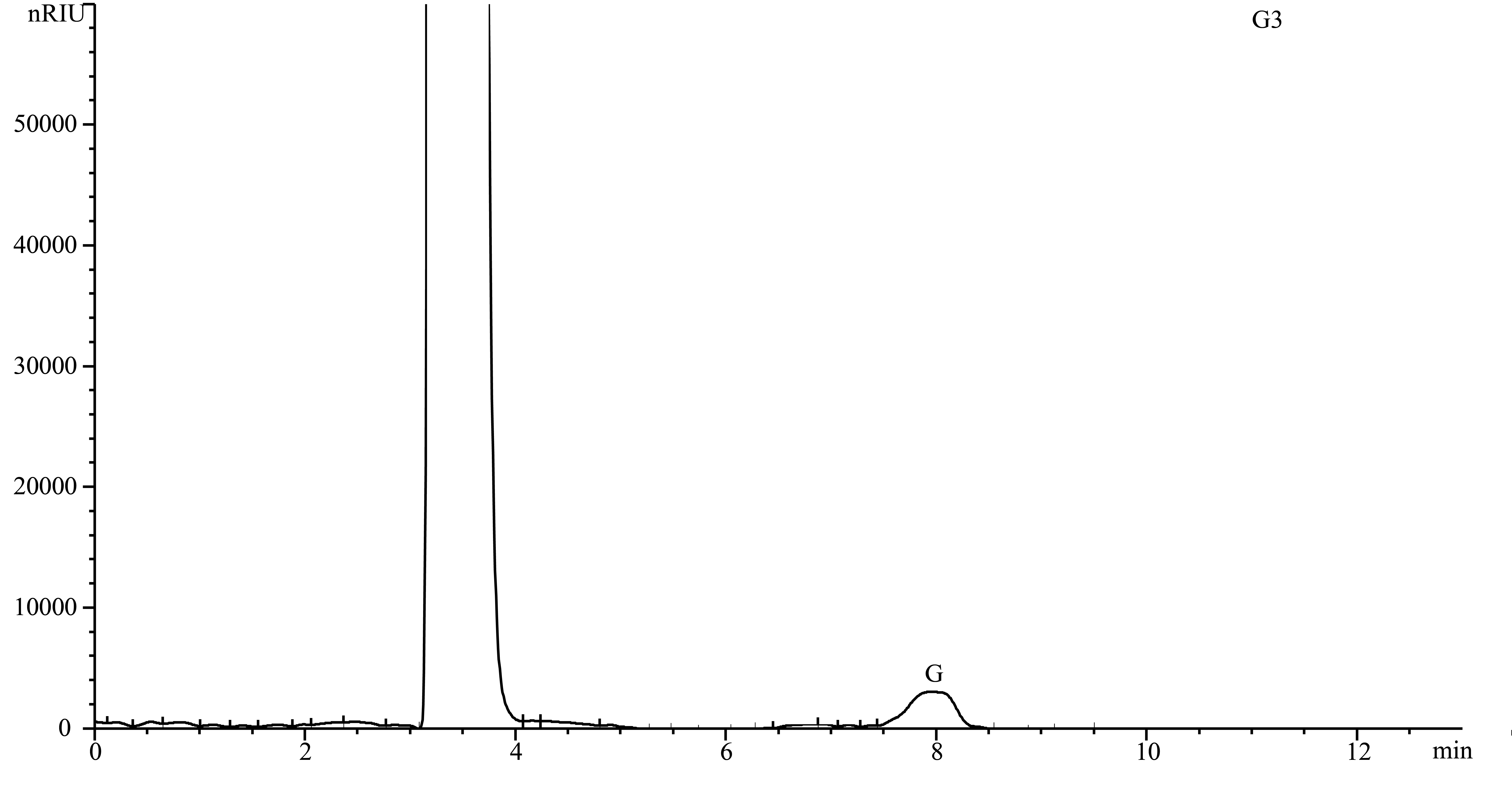


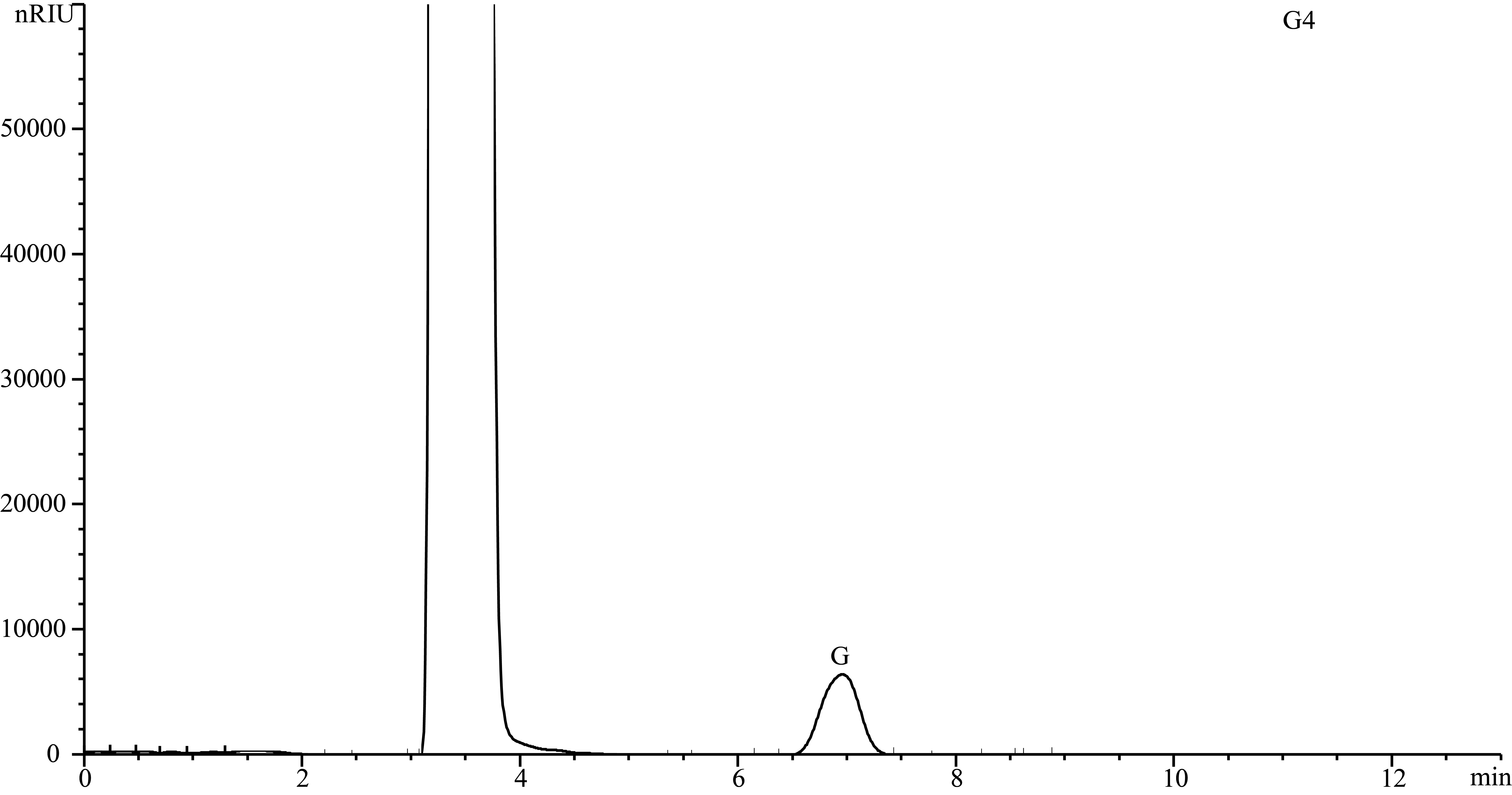

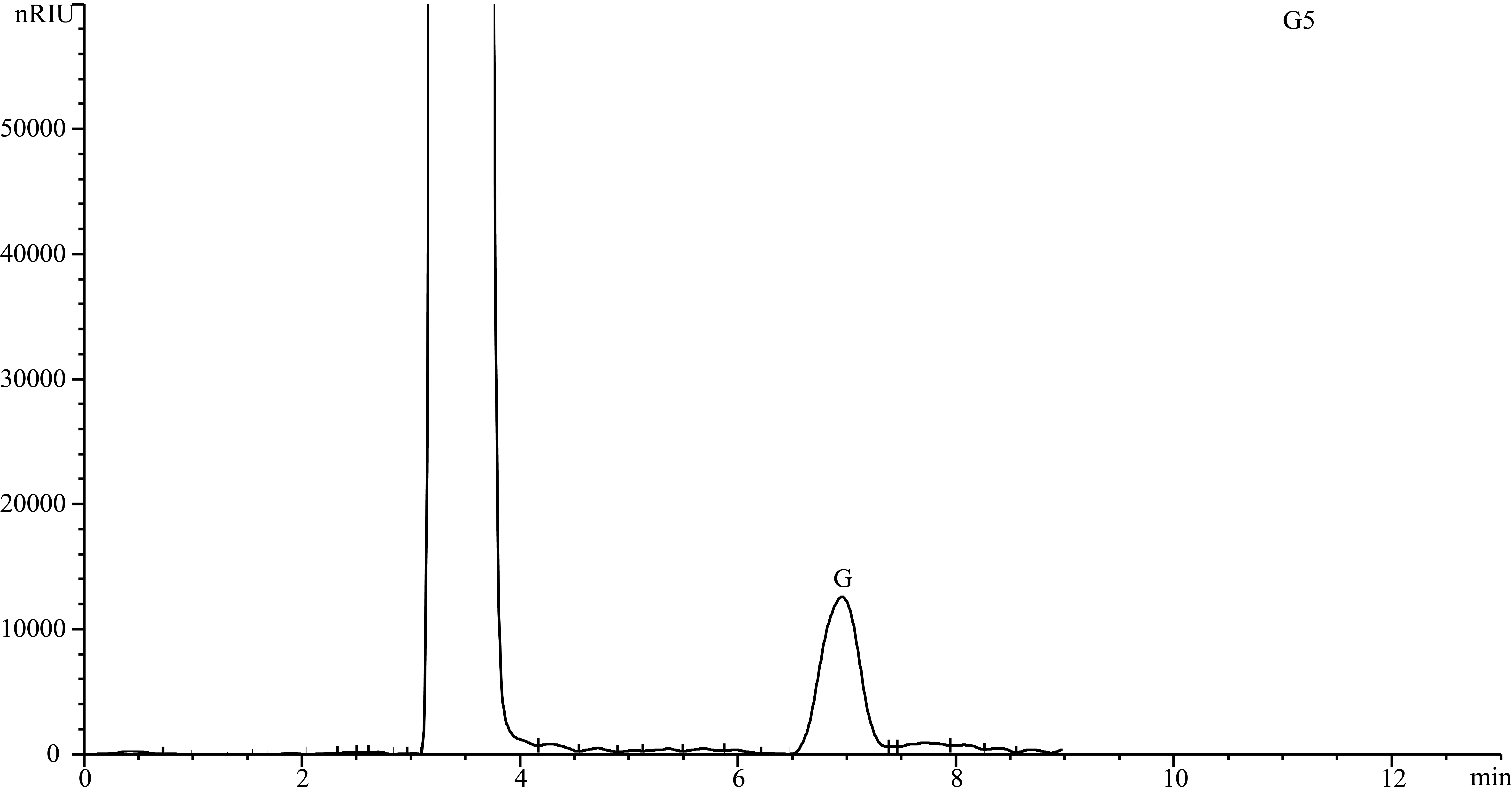

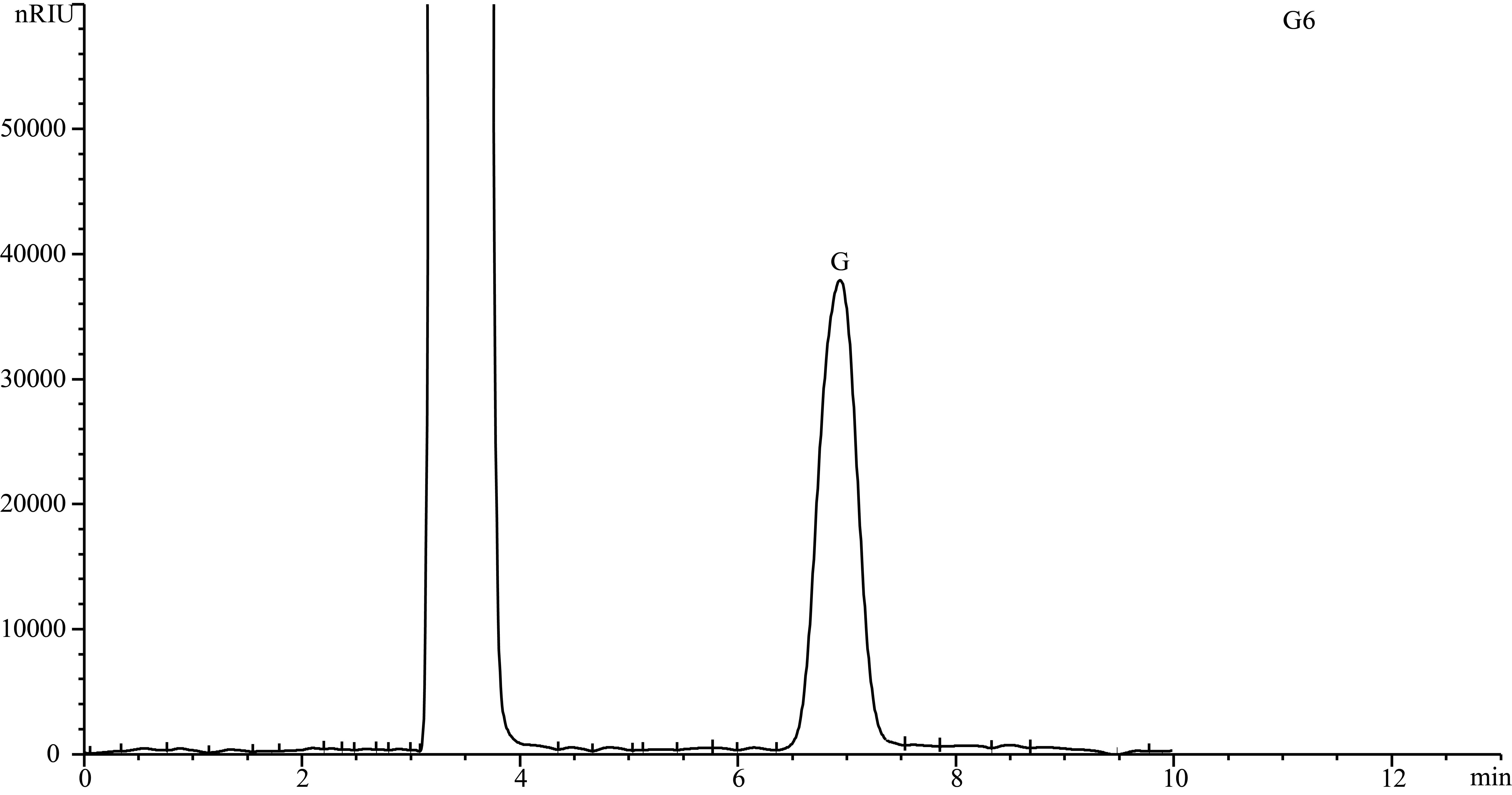


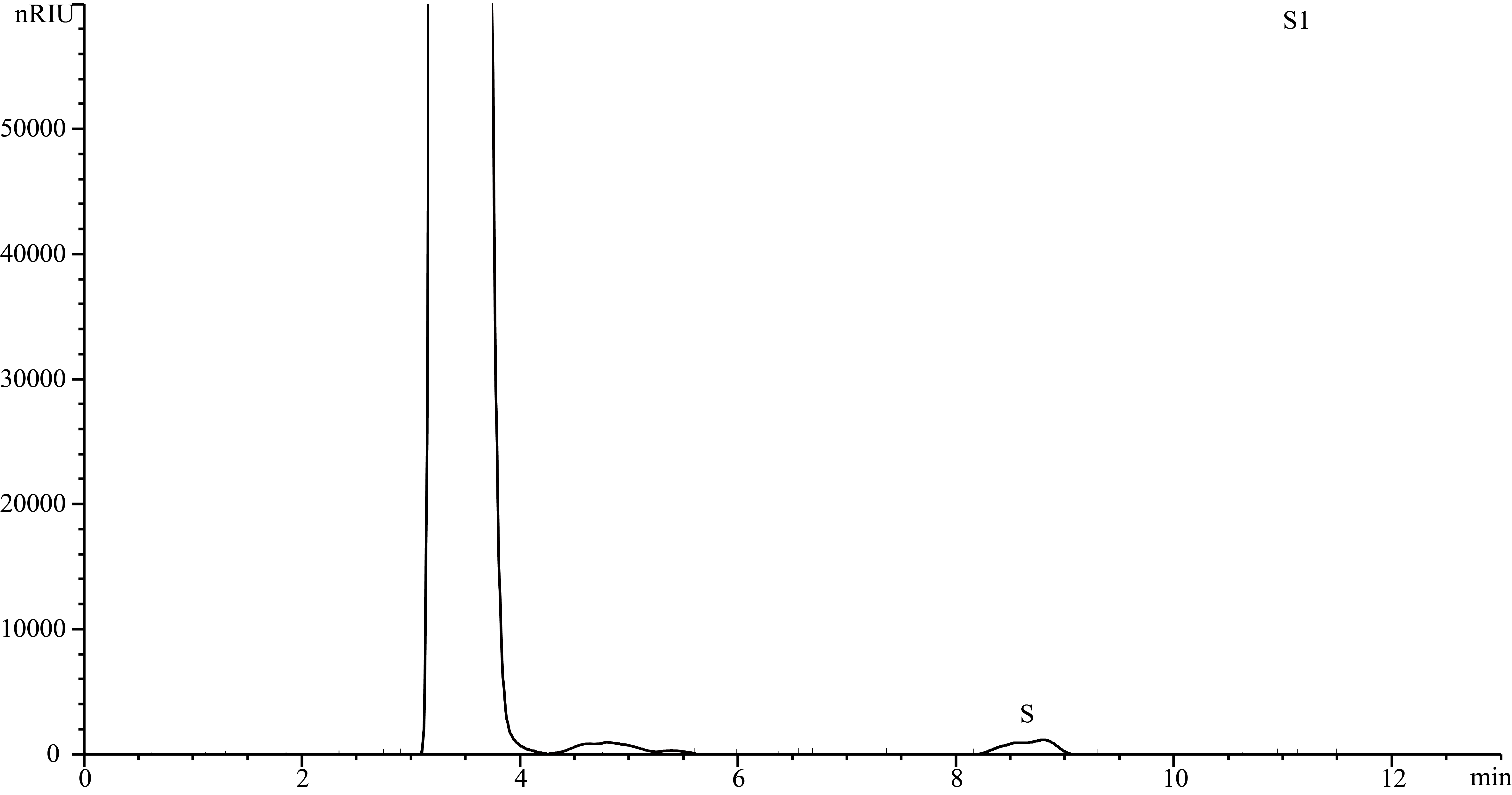

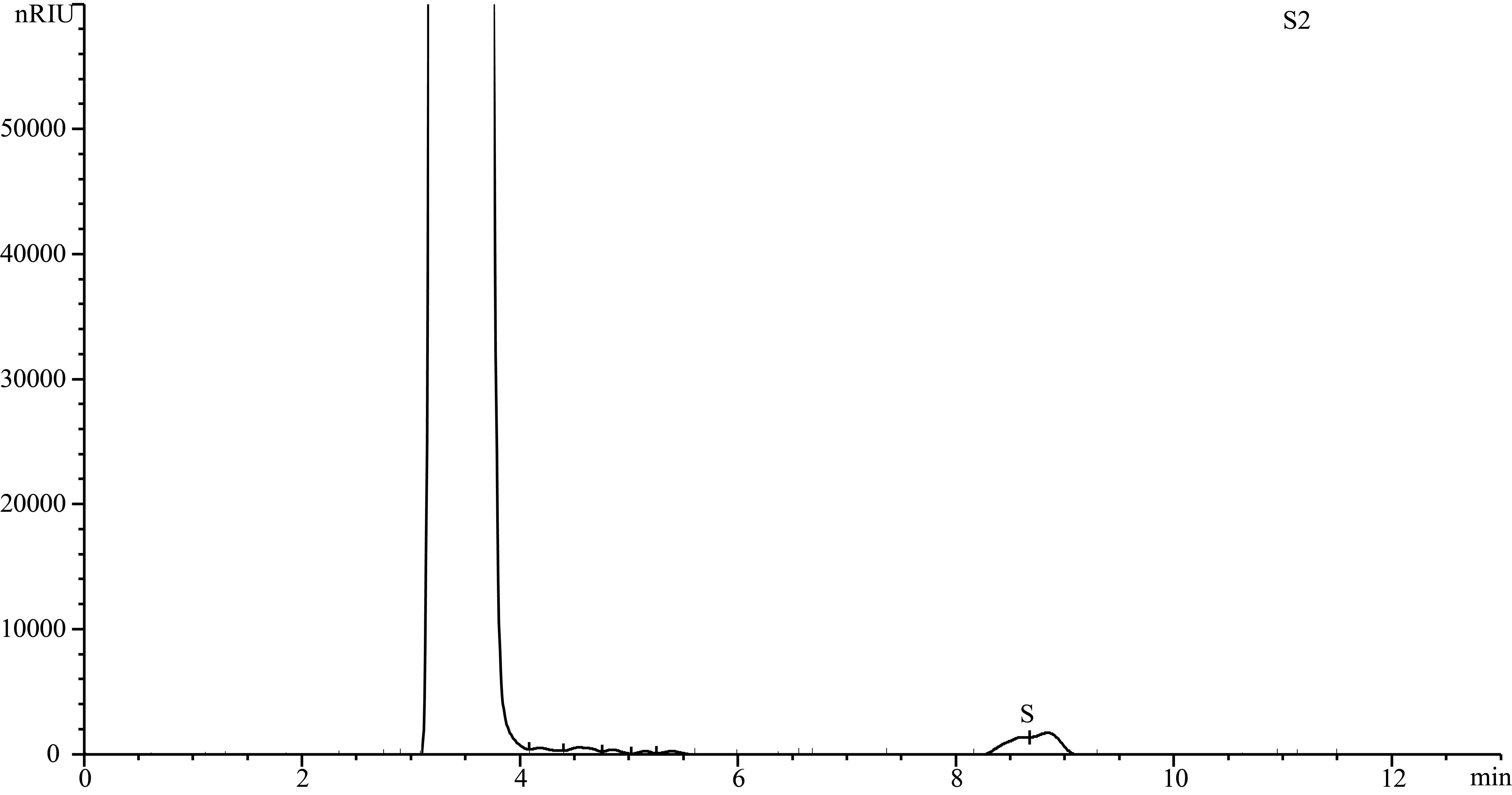

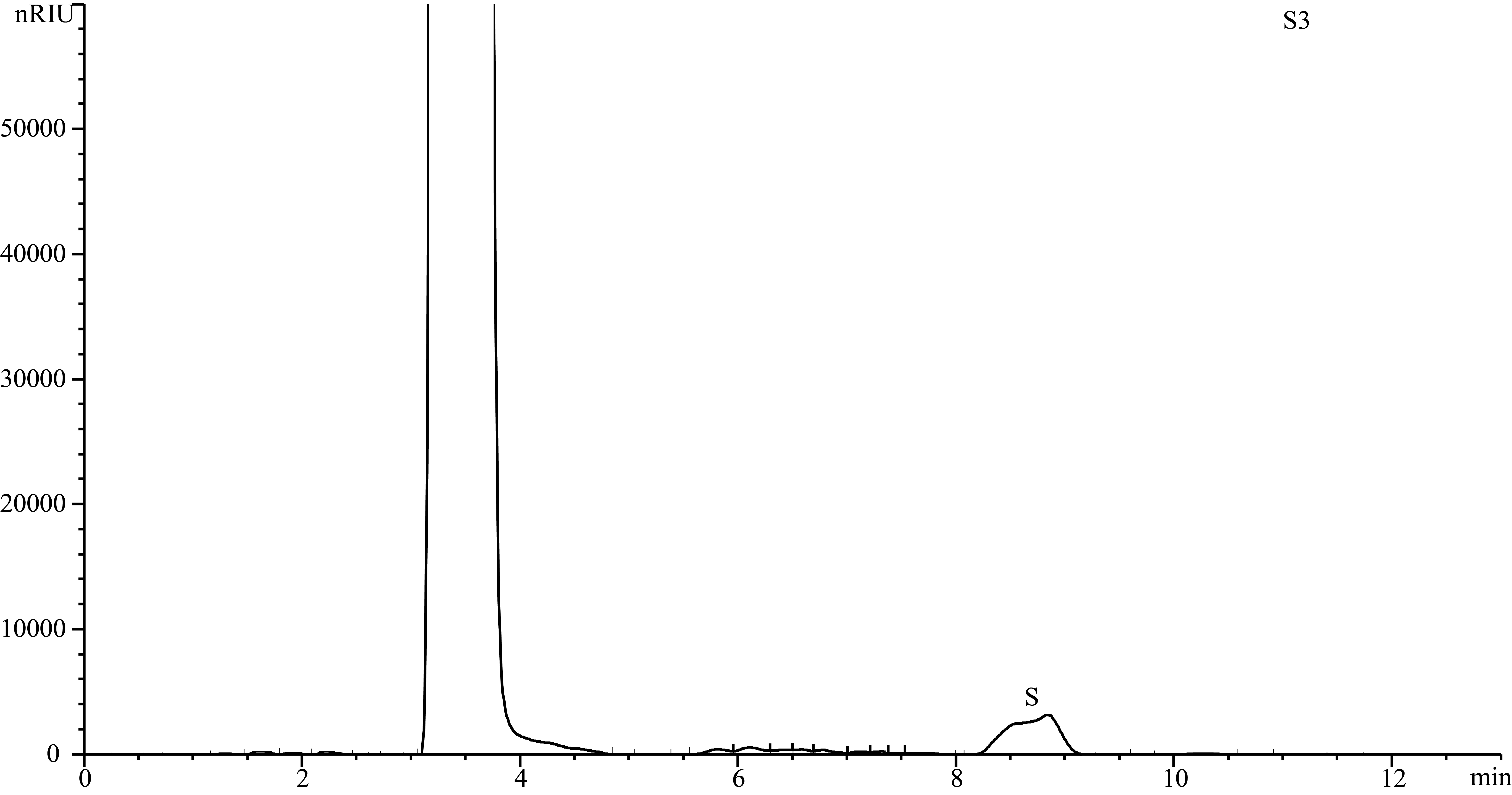


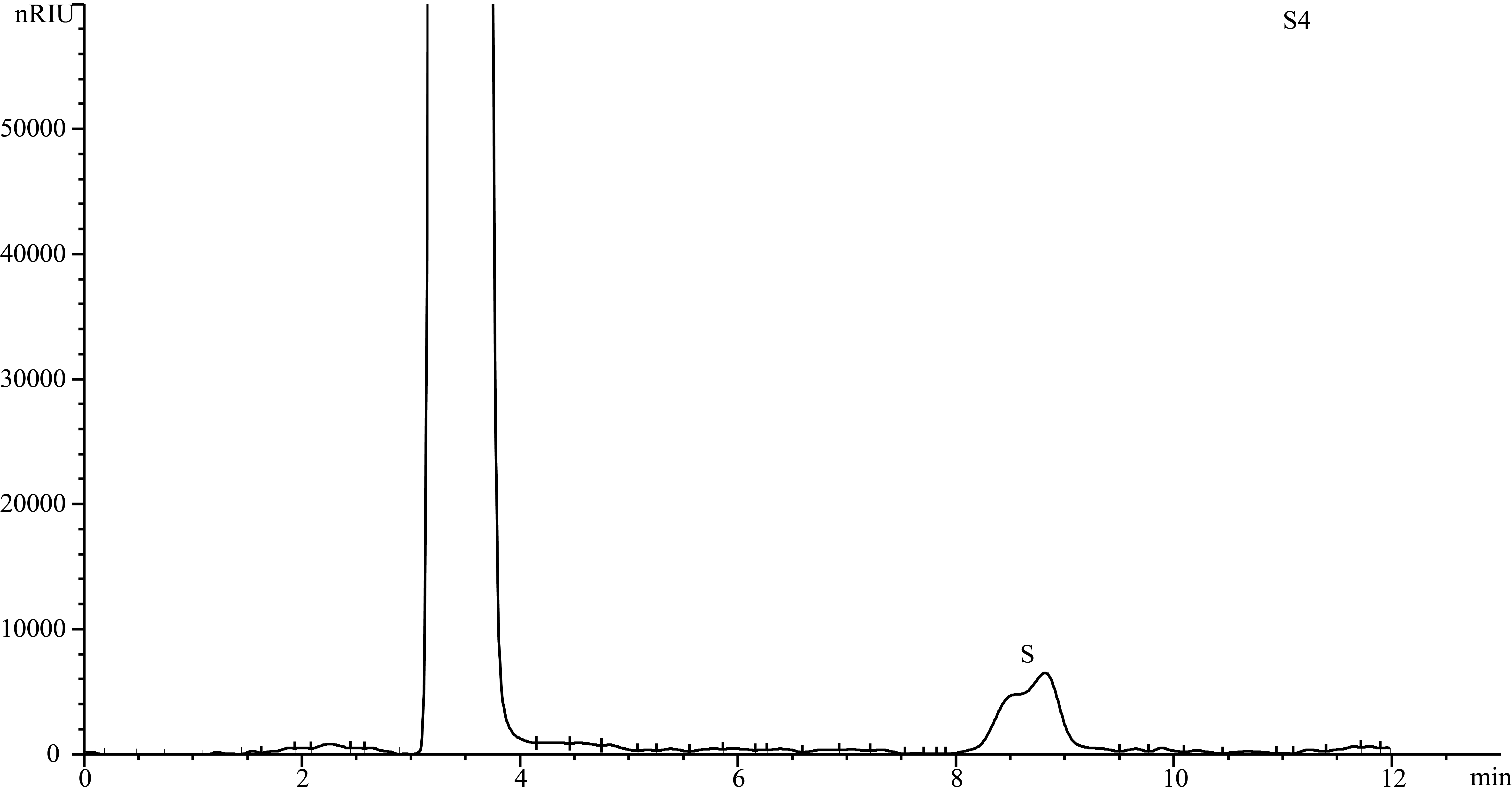

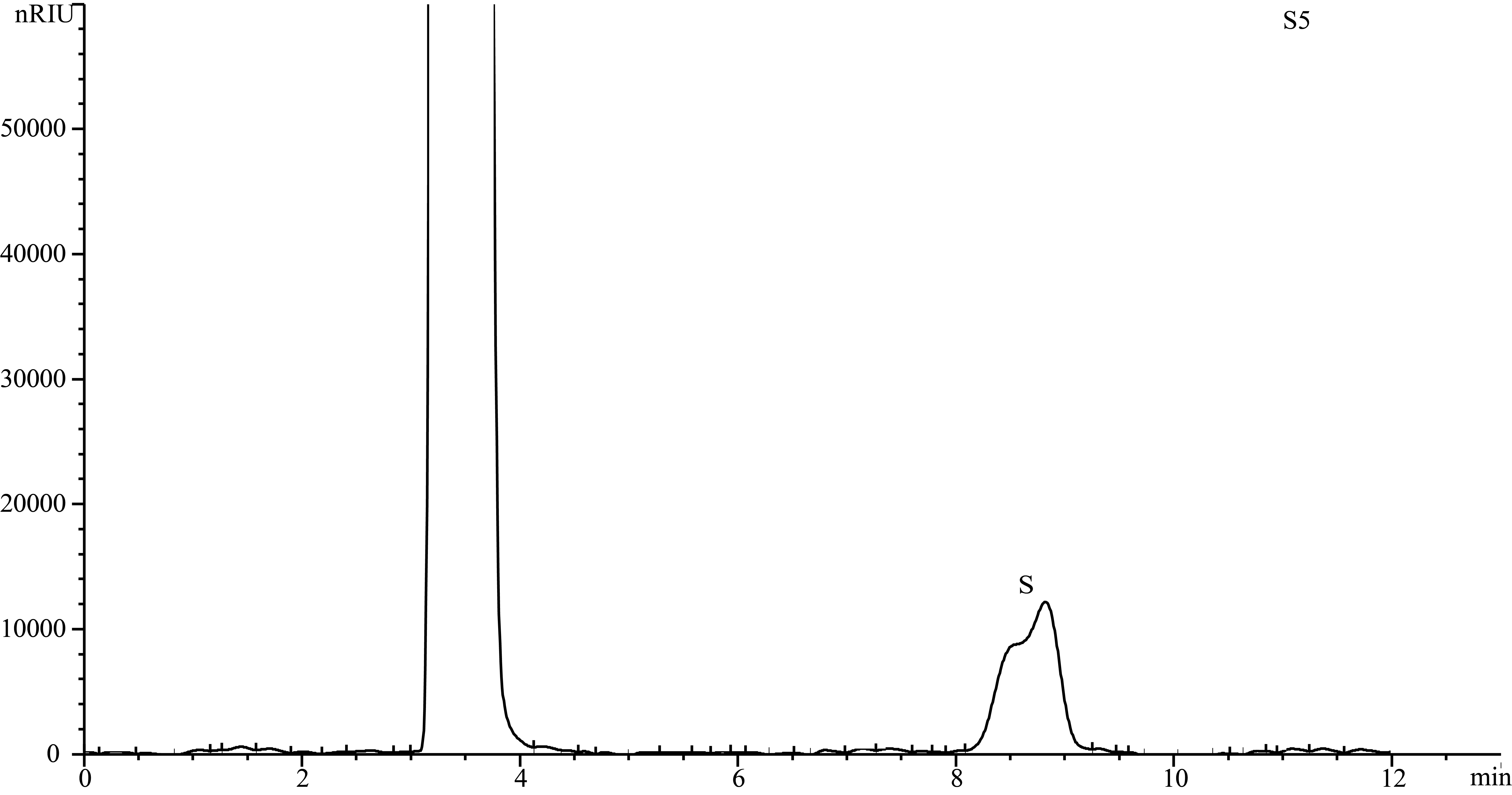

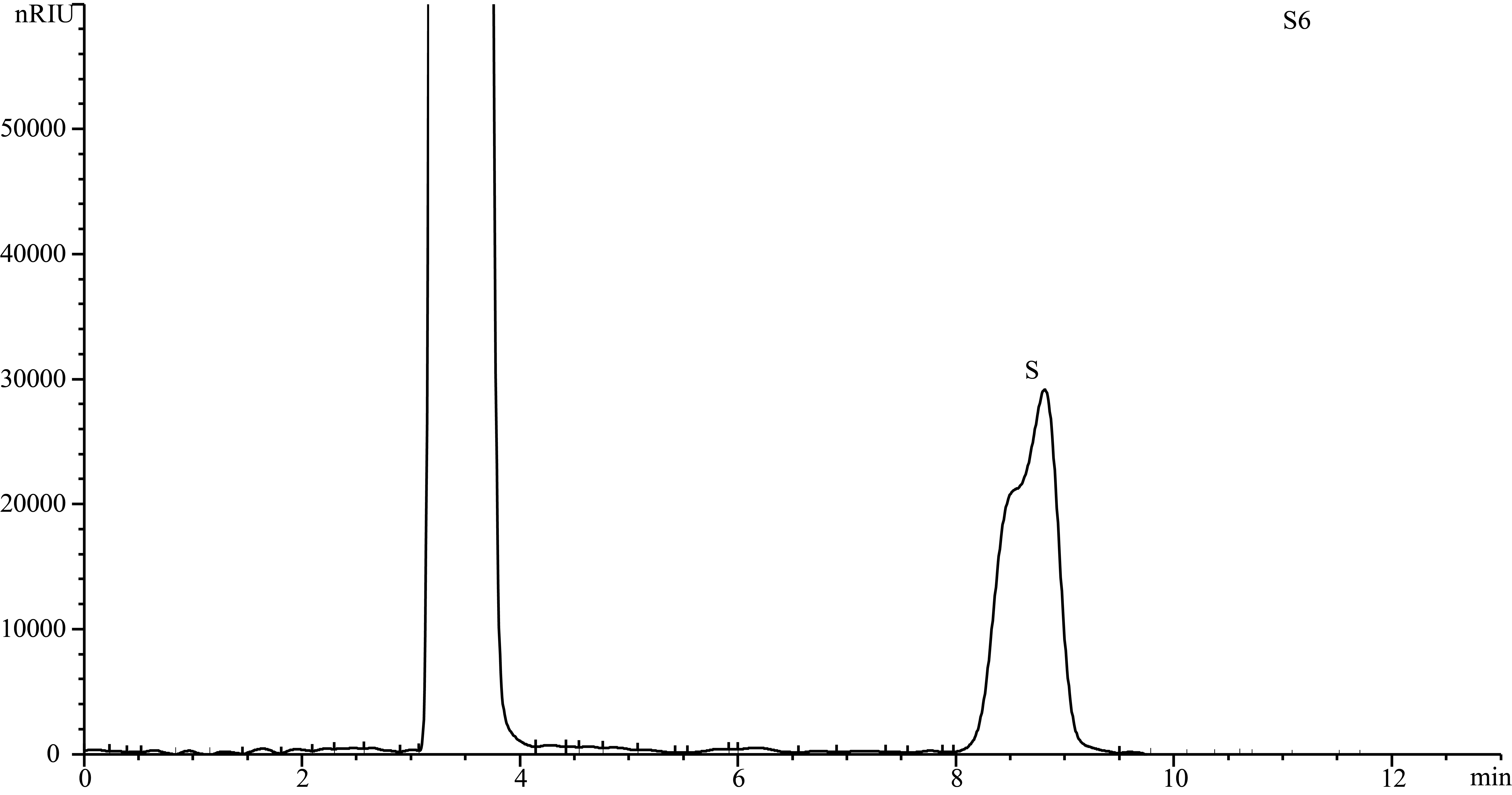


Figure S3. The liquid chromatography of fructose, glucose and sucrose standards was as shown in the figure. 6 concentrations were determined, 1, 2, 3, 4, 5 and 6 respectively representing 0.04, 0.2, 0.5, 1, 2 and 5 mg · mL^-1^.
